# Supplementary figures and images for: Tertiary lymphoid structures-driven immune infiltration patterns and their association with survival in neuroblastoma
Source: PeerJ. 2025 Jul 22;13:e19767. doi: 10.7717/peerj.19767 (PMC12292307; doi:10.7717/peerj.19767)

A

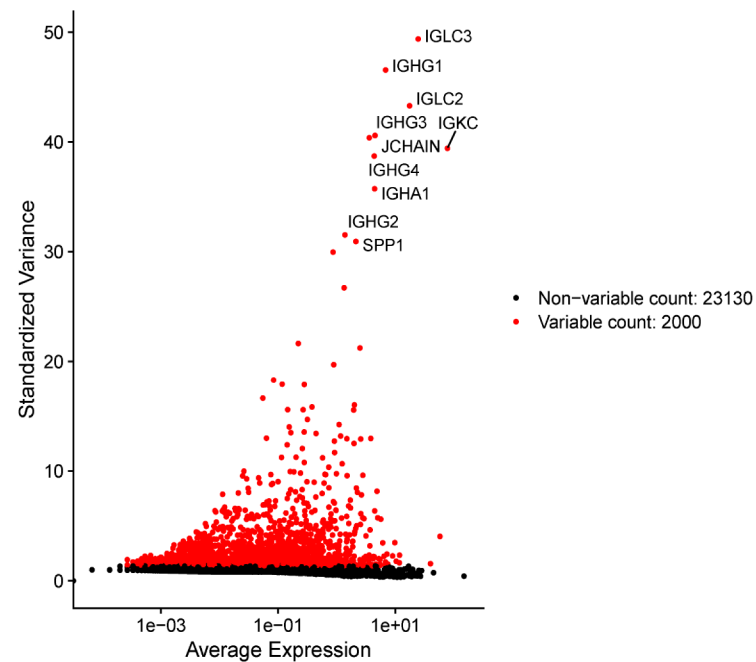

B

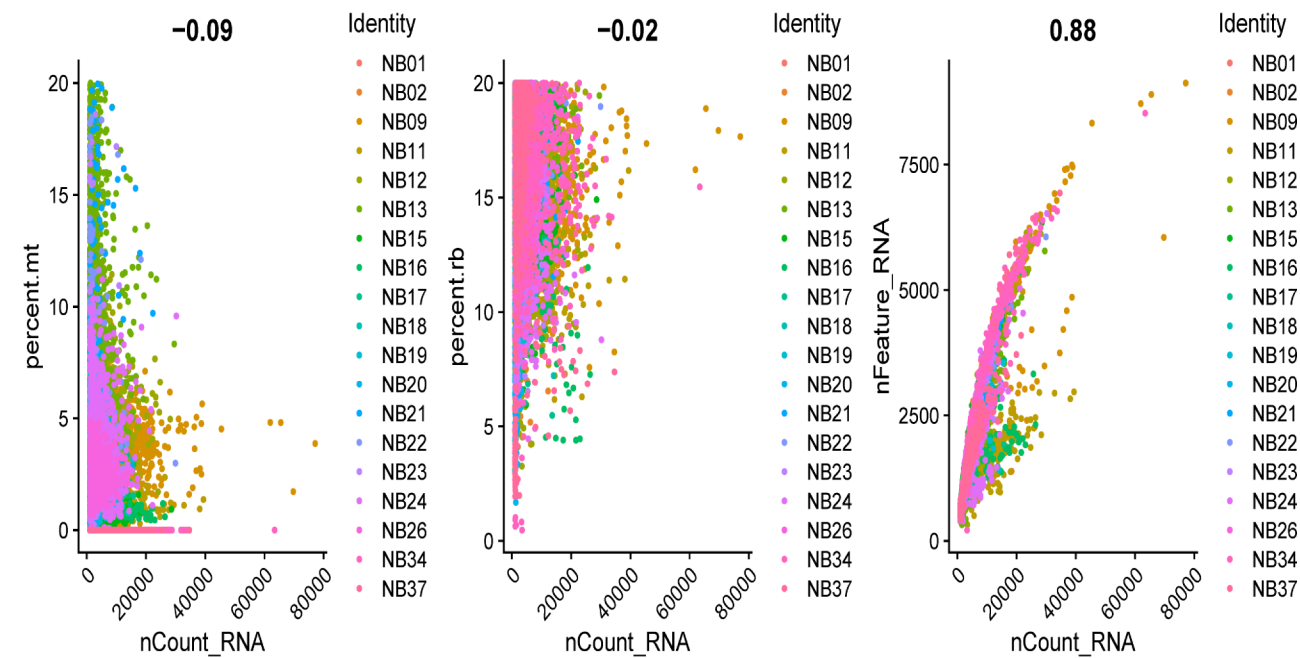

F

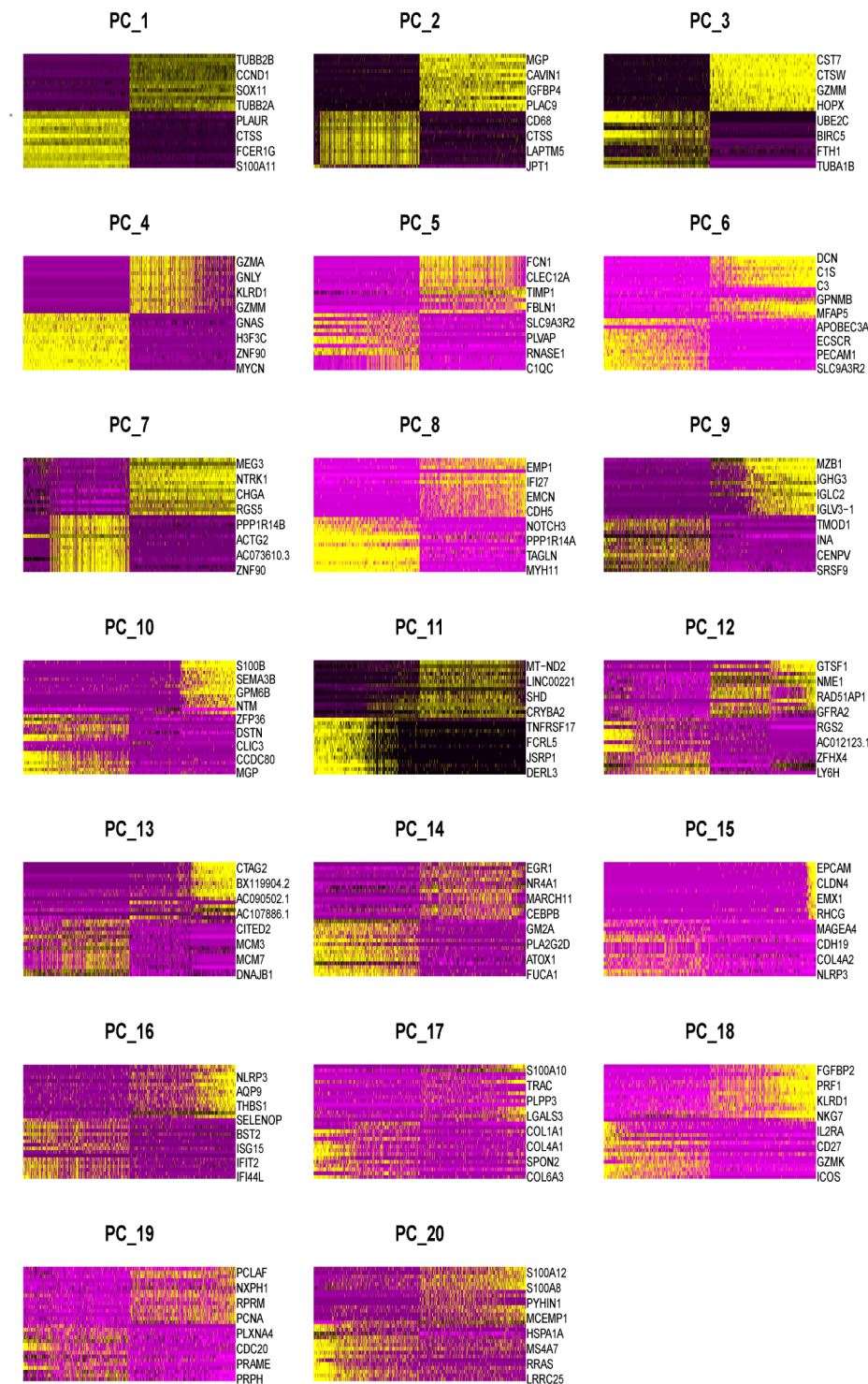

C

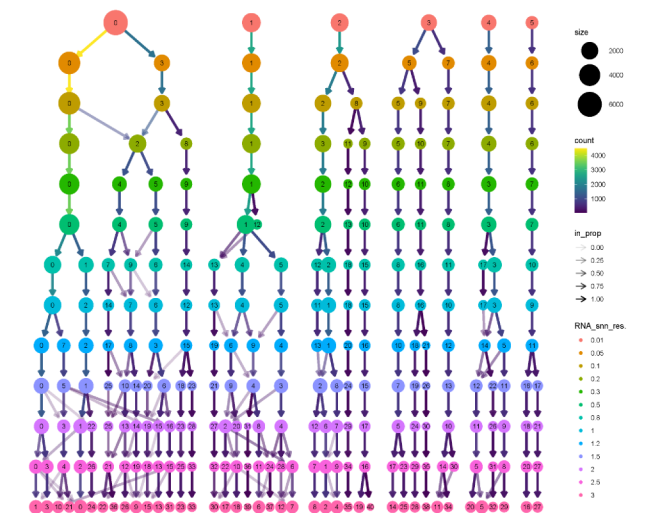

D

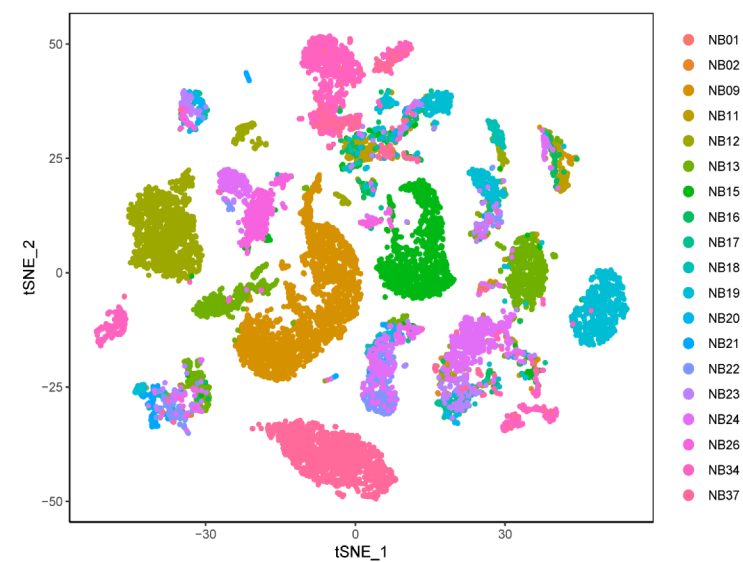

E

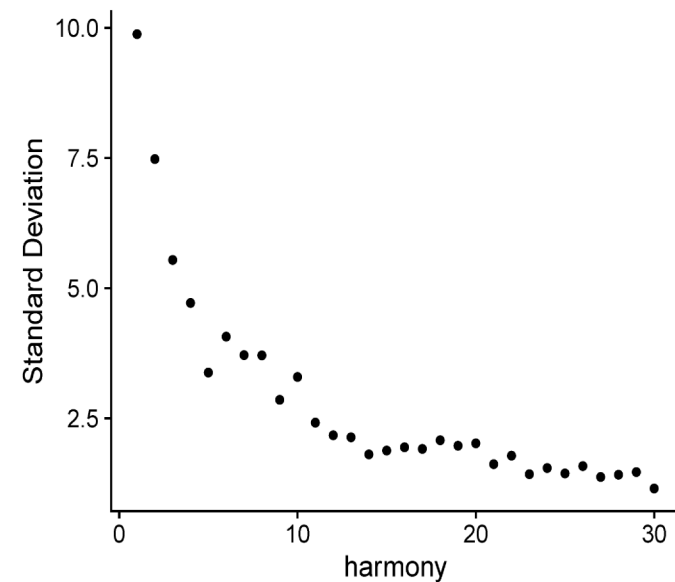

Supplement: Supplemental Information 1 [file peerj-13-19767-s001.pdf]

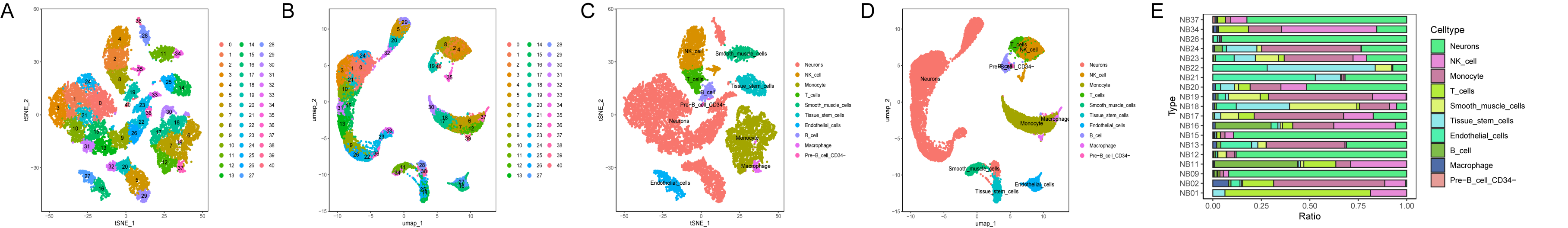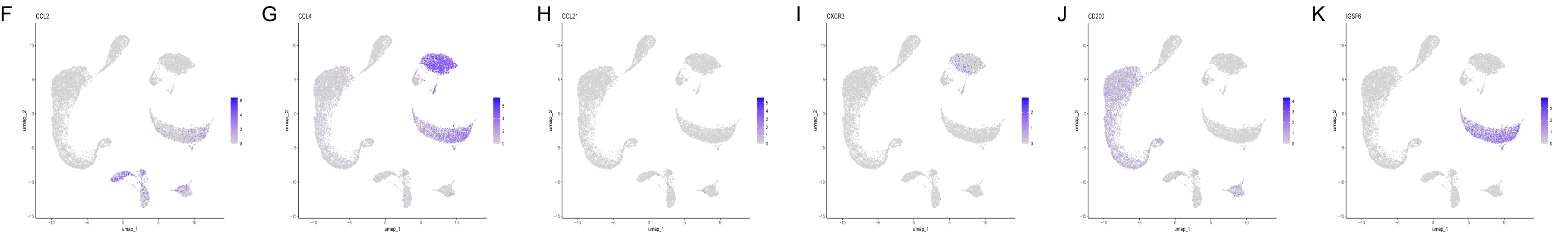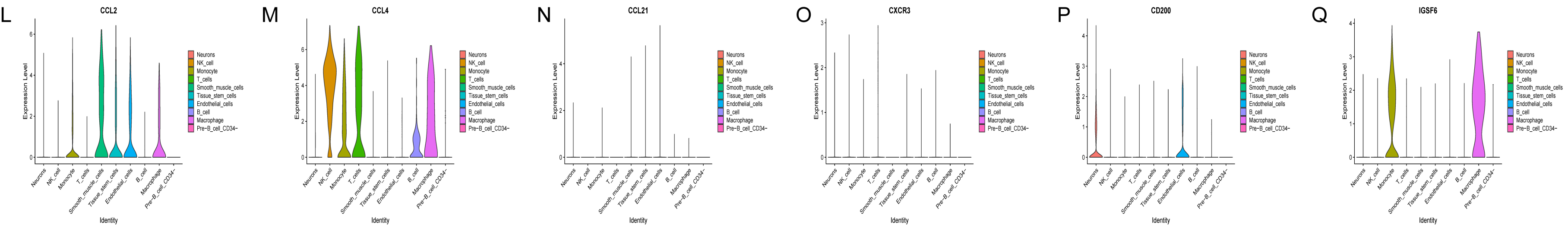

Supplement: Supplemental Information 3 — A-D, the results of tSNE and UMAP clustering following dimensionality reduction and annotation. Different colors correspond to distinct cell populations. E, the proportion bar chart of annotated cells, where each bar represents a NB sample, and different colors represent various cell groups. F-K, the distribution of six TLS genes within subpopulations in the UMAP plot, with blue dots representing highly concentrated areas. L-Q, the mRNA expression levels of the six TLS genes across different cell populations, with each color corresponding to a specific cell subset and the y-axis indicating the level of expression. [file peerj-13-19767-s003.pdf]

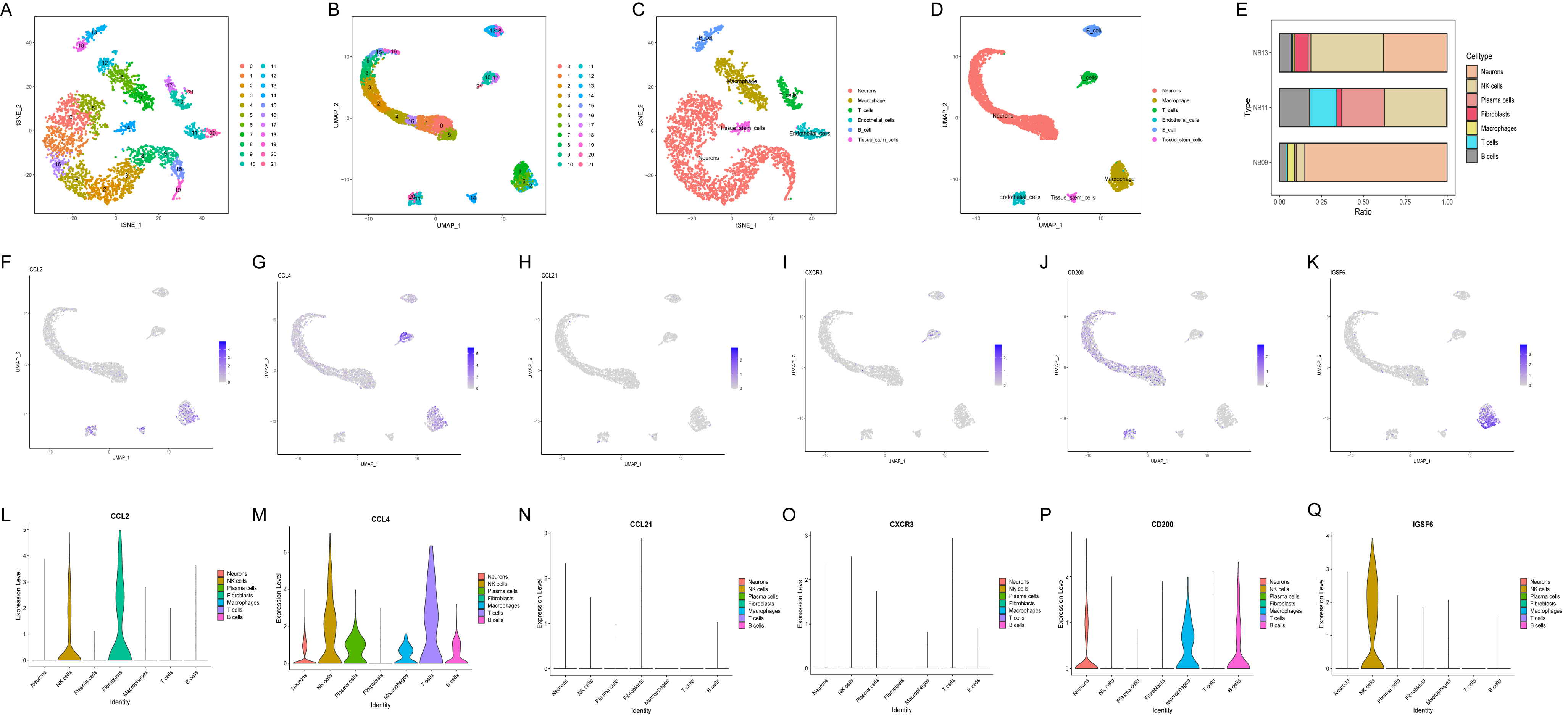

Supplement: Supplemental Information 4 — A-D, the results of tSNE and UMAP clustering following dimensionality reduction and annotation. Different colors correspond to distinct cell populations. E, the proportion bar chart of annotated cells, where each bar represents a NB sample, and different colors represent various cell groups. F-K, the distribution of six TLS genes within subpopulations in the UMAP plot, with blue dots representing highly concentrated areas. L-Q, the mRNA expression levels of the six TLS genes across different cell populations, with each color corresponding to a specific cell subset and the y-axis indicating the level of expression. [file peerj-13-19767-s004.pdf]

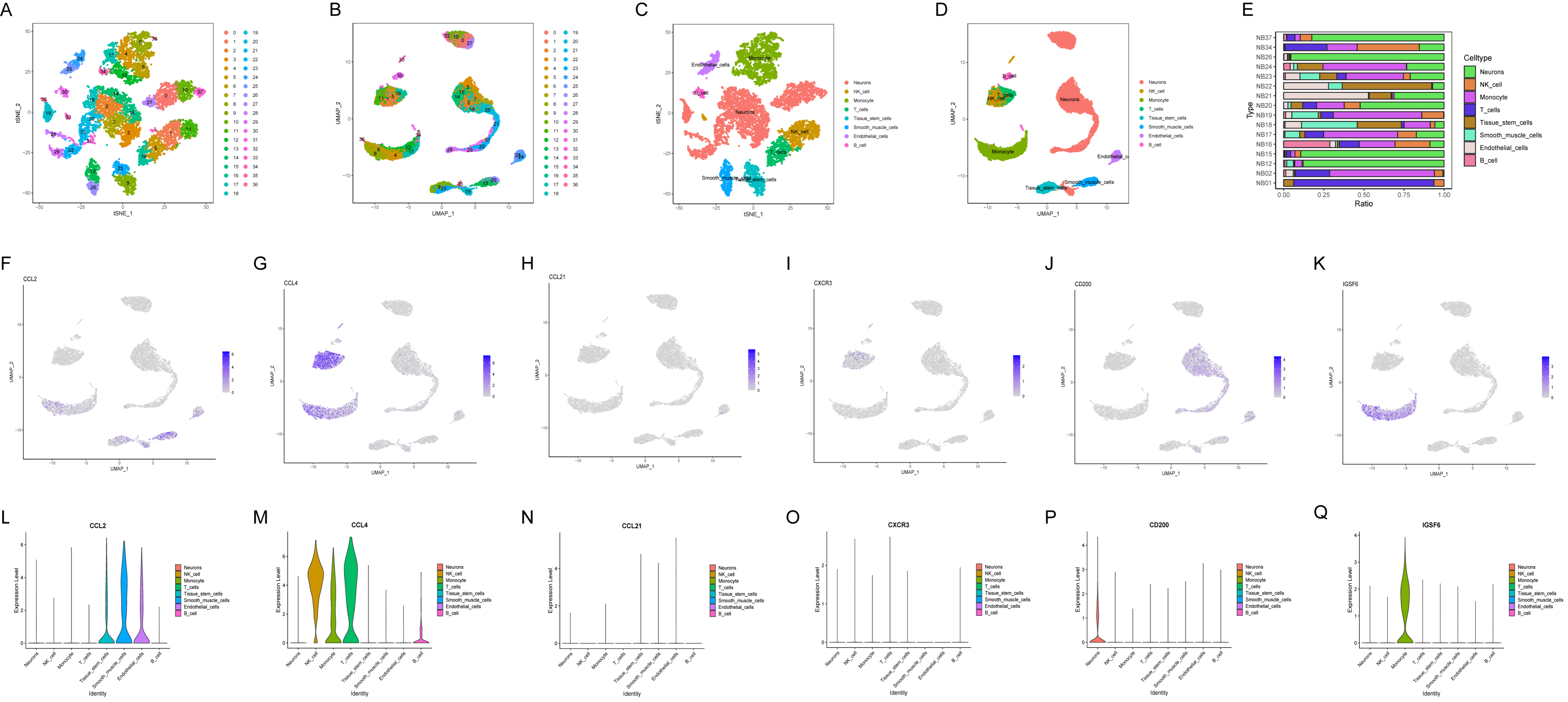

Supplement: Supplemental Information 5 — A-D display the results of tSNE and UMAP clustering following dimensionality reduction and annotation. Different colors correspond to distinct cell populations. Panel E presents the proportion bar chart of annotated cells, where each bar represents a NB sample, and different colors represent various cell groups. Panels F-K illustrate the distribution of six TLS genes within subpopulations in the UMAP plot, with blue dots representing highly concentrated areas. Panels L-Q show the mRNA expression levels of the six TLS genes across different cell populations, with each color corresponding to a specific cell subset and the y-axis indicating the level of expression. [file peerj-13-19767-s005.pdf]

## GSE49710\_EFS

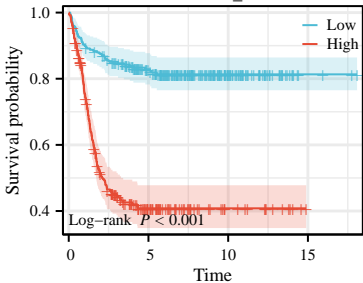

|      |     |     |    |   |
|------|-----|-----|----|---|
| Low  | 249 | 165 | 56 | 3 |
| High | 249 | 55  | 13 | 0 |

Supplement: Supplemental Information 6 [file peerj-13-19767-s006.zip › Raw Data/RNA-seq/05.KM/K-M/GSE49710/EFS/K-M.pdf]

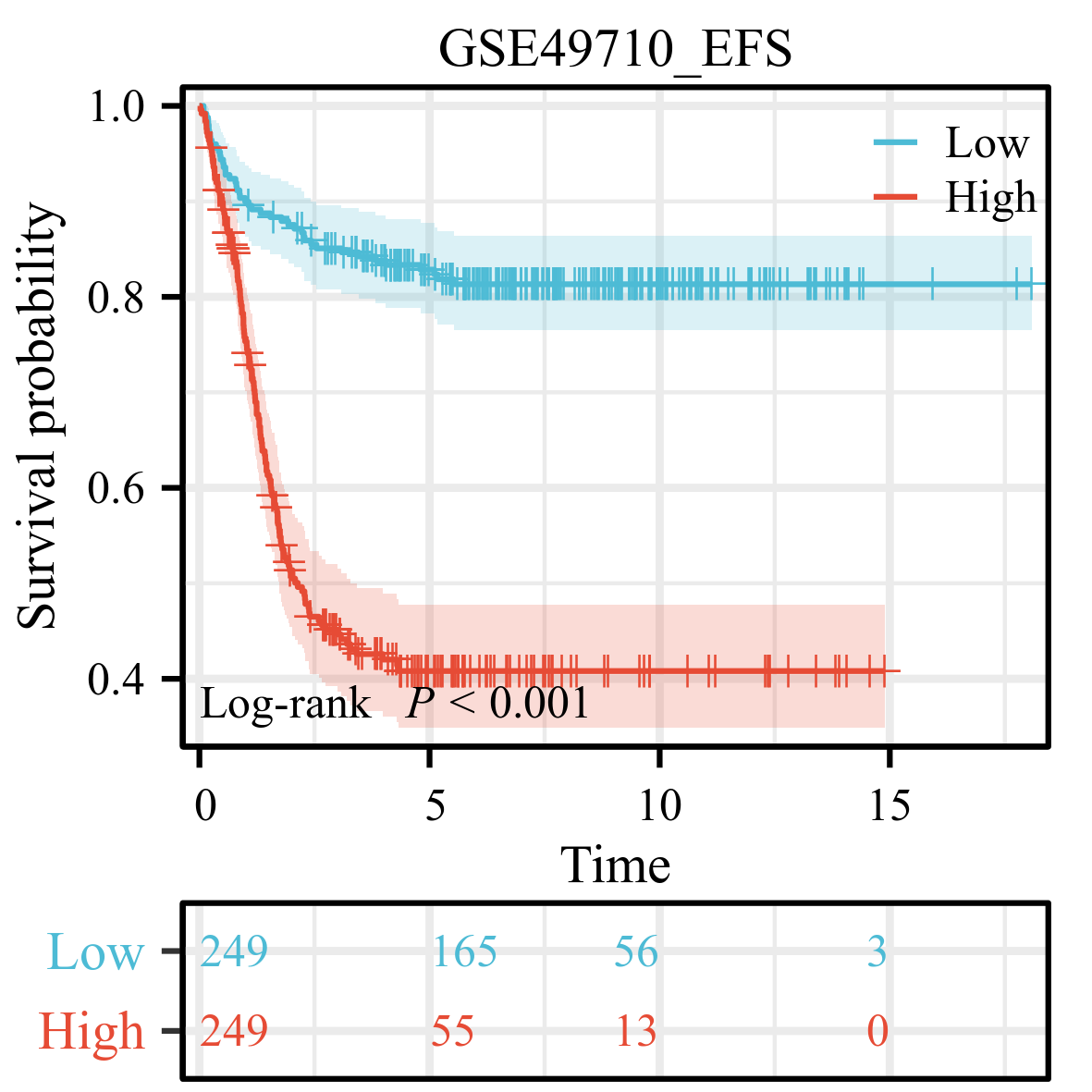

Supplement: Supplemental Information 6 [file peerj-13-19767-s006.zip › Raw Data/RNA-seq/05.KM/K-M/GSE49710/EFS/K-M.tiff]

## GSE49710\_OS

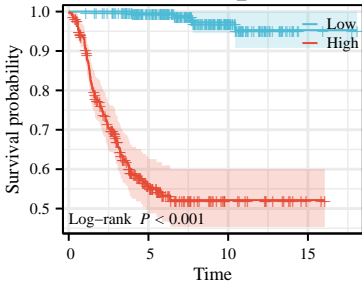

|      |     |     |    |   |
|------|-----|-----|----|---|
| Low  | 249 | 197 | 61 | 3 |
| High | 249 | 72  | 17 | 2 |

Supplement: Supplemental Information 6 [file peerj-13-19767-s006.zip › Raw Data/RNA-seq/05.KM/K-M/GSE49710/OS/K-M.pdf]

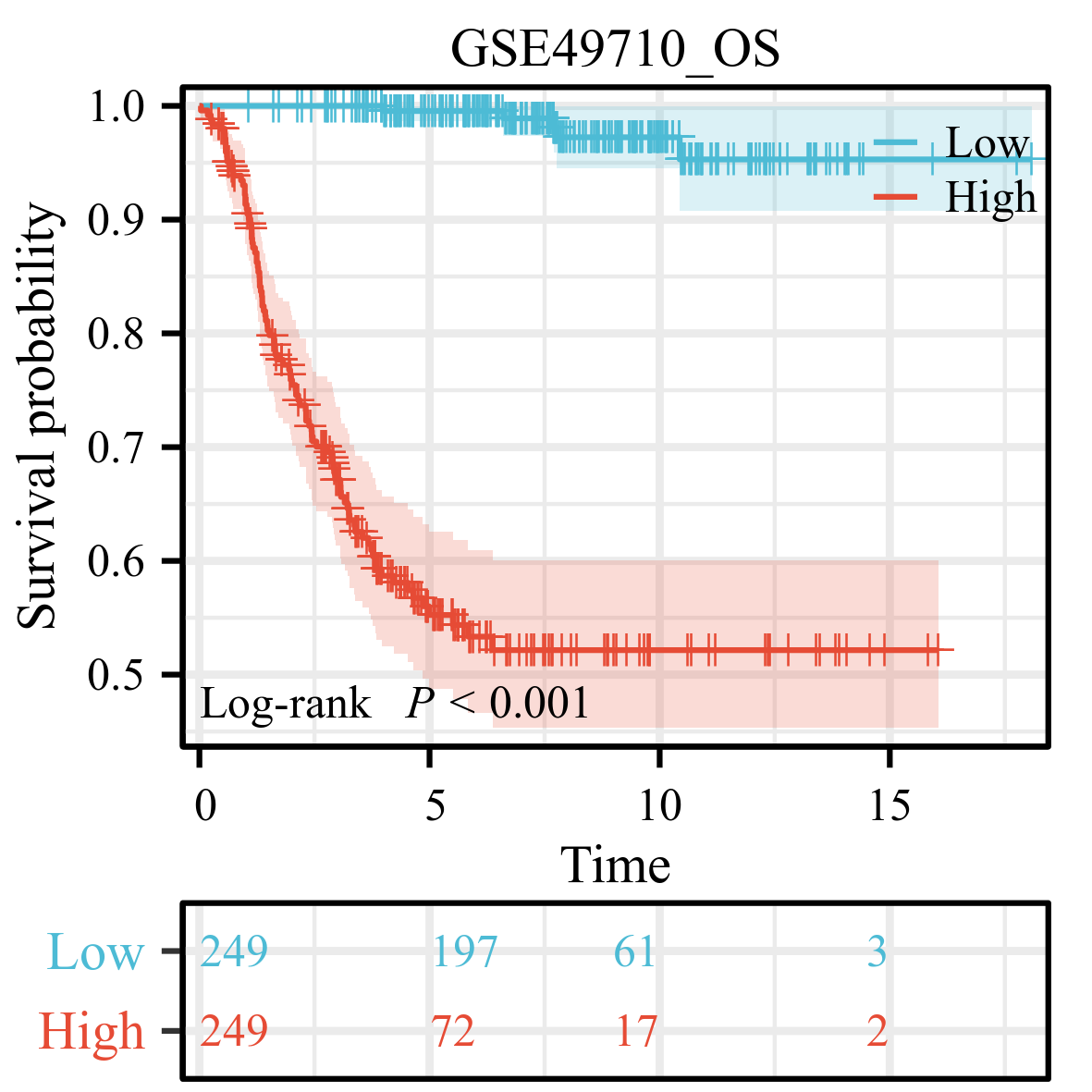

Supplement: Supplemental Information 6 [file peerj-13-19767-s006.zip › Raw Data/RNA-seq/05.KM/K-M/GSE49710/OS/K-M.tiff]

## GSE62564\_EFS

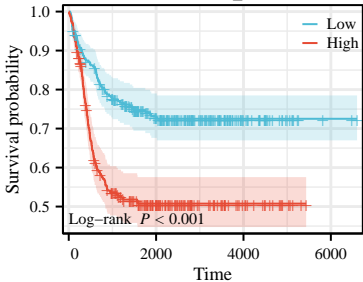

Low

249

128

30

2

High

249

78

18

0

Supplement: Supplemental Information 6 [file peerj-13-19767-s006.zip › Raw Data/RNA-seq/05.KM/K-M/GSE62564/EFS/K-M.pdf]

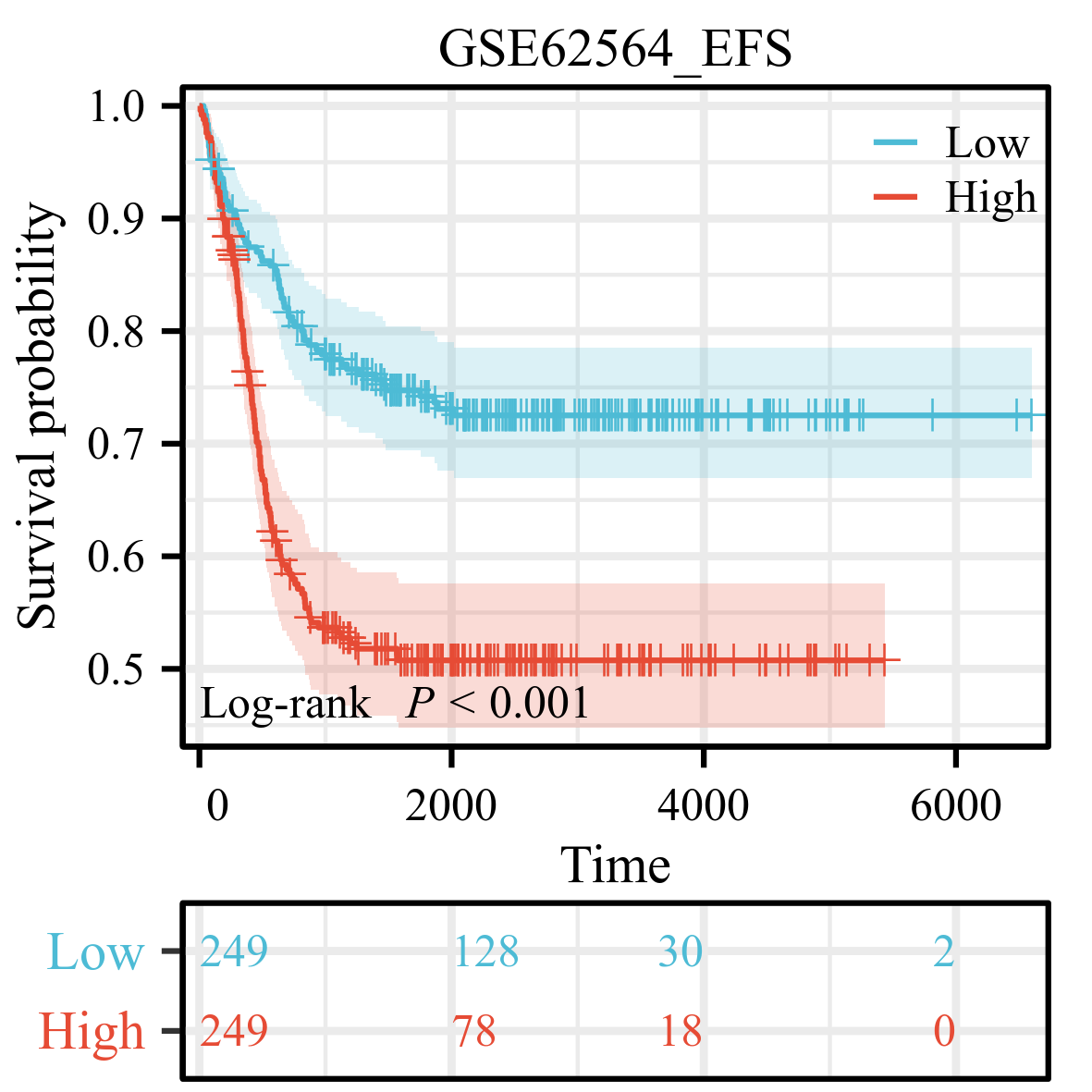

Supplement: Supplemental Information 6 [file peerj-13-19767-s006.zip › Raw Data/RNA-seq/05.KM/K-M/GSE62564/EFS/K-M.tiff]

## GSE62564\_OS

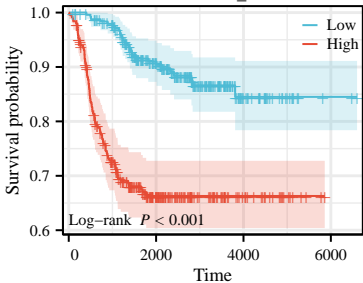

Low

249

156

30

2

High

249

94

22

0

Supplement: Supplemental Information 6 [file peerj-13-19767-s006.zip › Raw Data/RNA-seq/05.KM/K-M/GSE62564/OS/K-M.pdf]

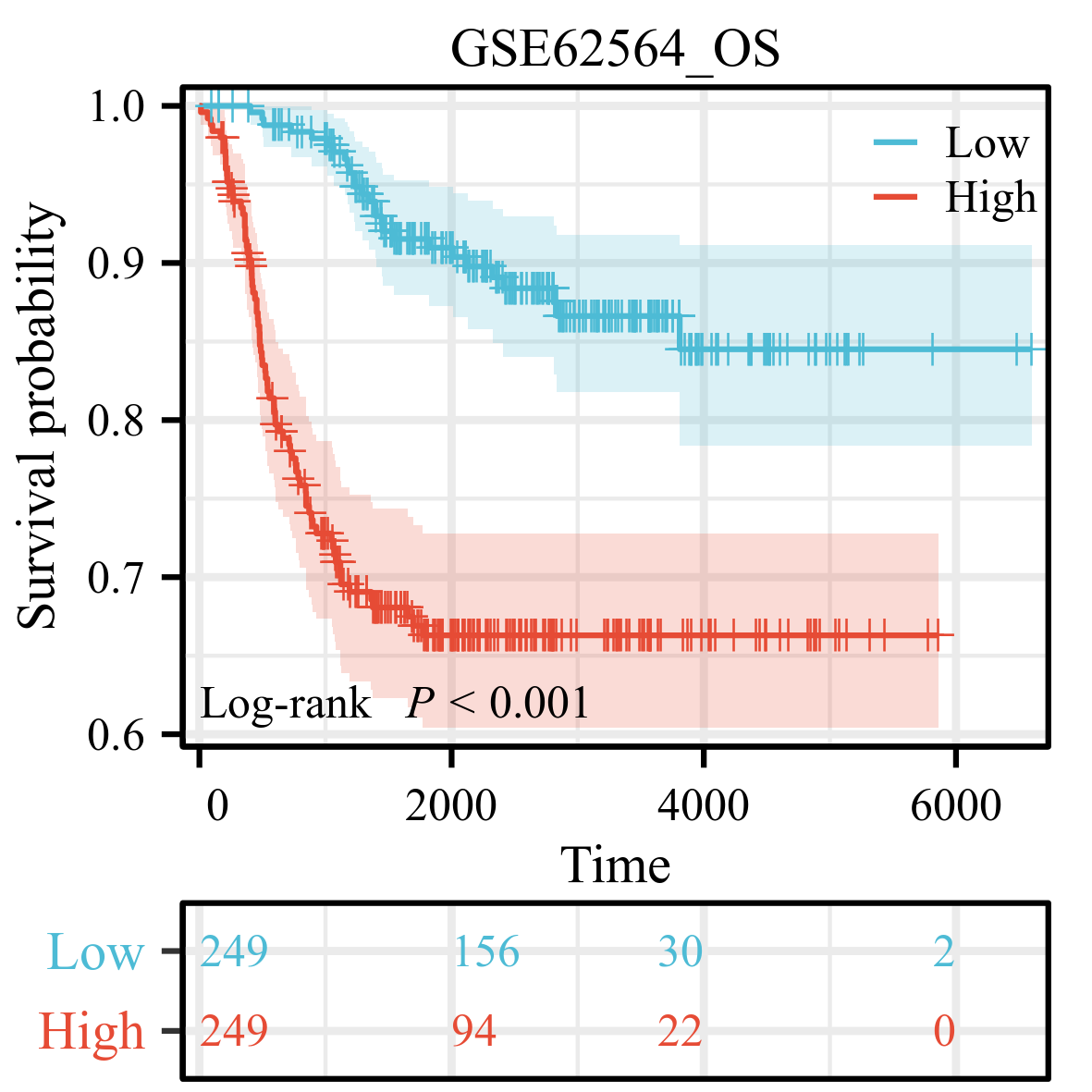

Supplement: Supplemental Information 6 [file peerj-13-19767-s006.zip › Raw Data/RNA-seq/05.KM/K-M/GSE62564/OS/K-M.tiff]

GSE49710\_EFS

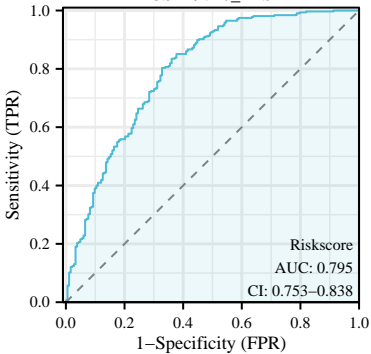

Supplement: Supplemental Information 6 [file peerj-13-19767-s006.zip › Raw Data/RNA-seq/08.ROC/GSE49710/EFS/ROC.pdf]

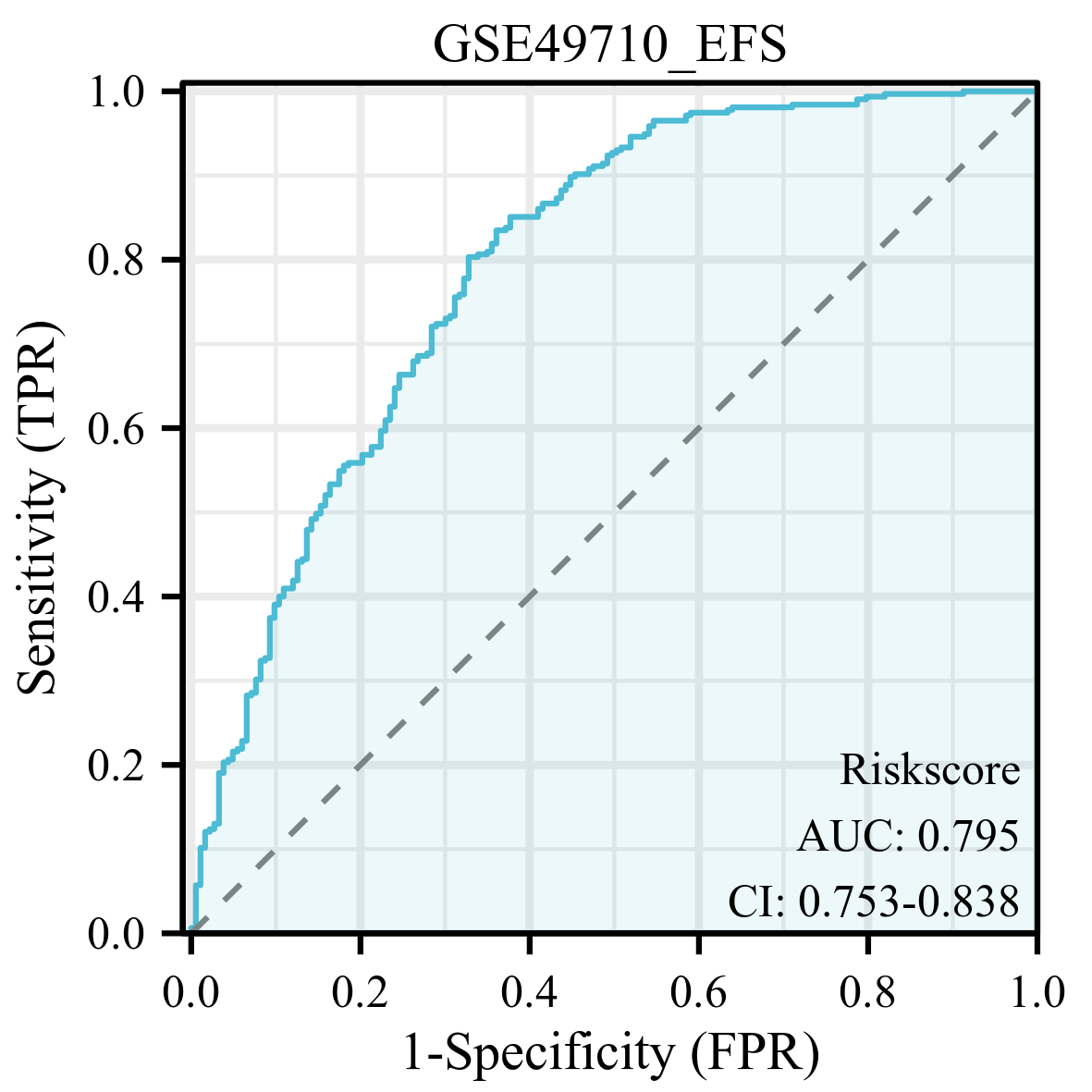

Supplement: Supplemental Information 6 [file peerj-13-19767-s006.zip › Raw Data/RNA-seq/08.ROC/GSE49710/EFS/ROC.tiff]

GSE49710\_OS

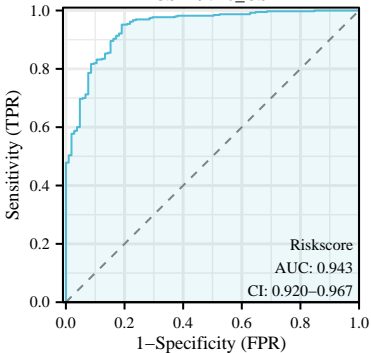

Supplement: Supplemental Information 6 [file peerj-13-19767-s006.zip › Raw Data/RNA-seq/08.ROC/GSE49710/OS/ROC.pdf]

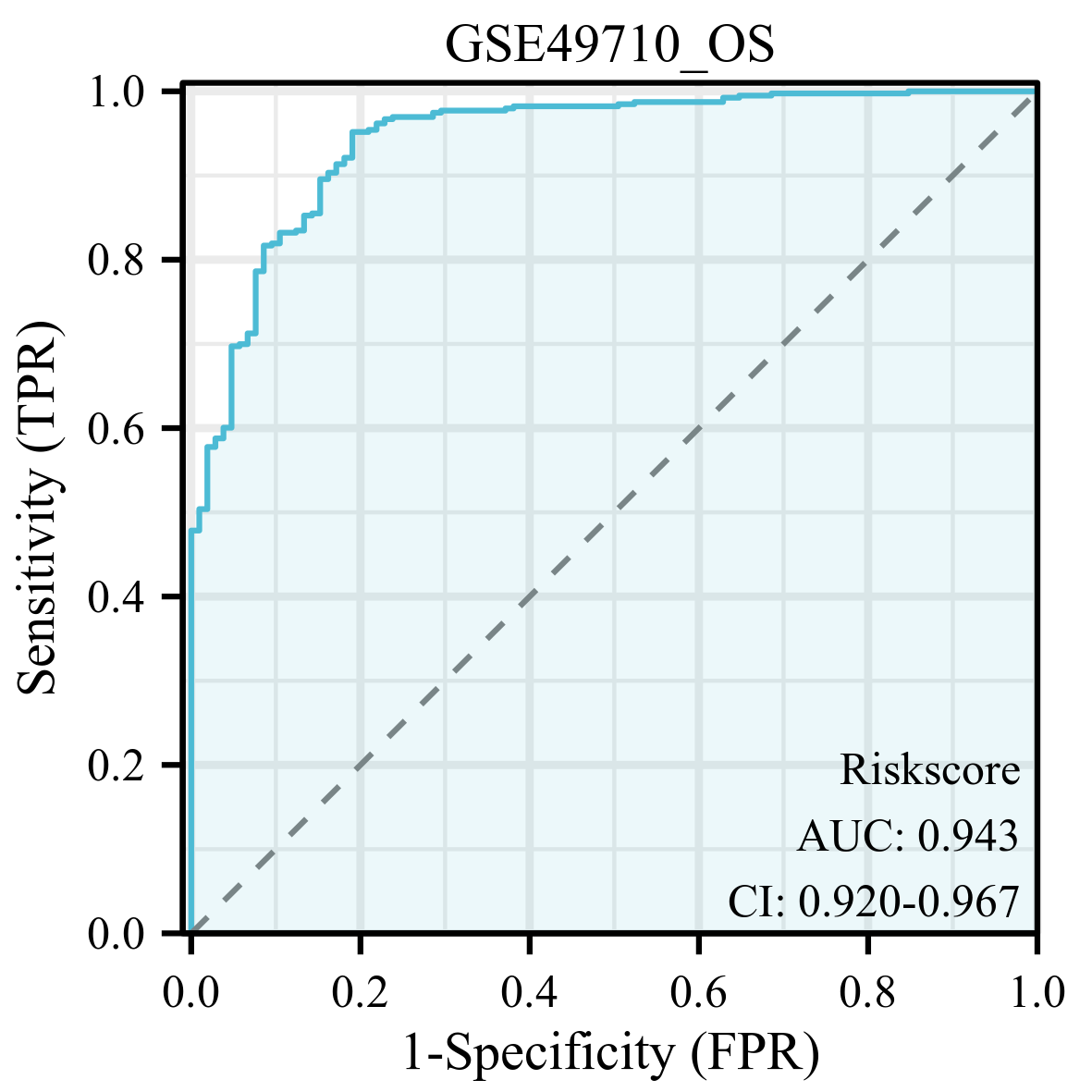

Supplement: Supplemental Information 6 [file peerj-13-19767-s006.zip › Raw Data/RNA-seq/08.ROC/GSE49710/OS/ROC.tiff]

GSE62564\_EFS

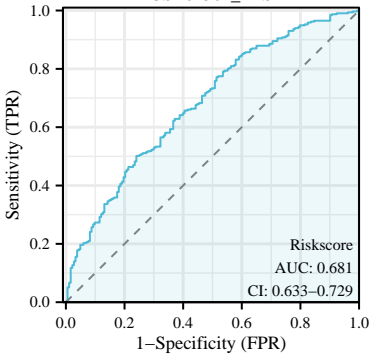

Supplement: Supplemental Information 6 [file peerj-13-19767-s006.zip › Raw Data/RNA-seq/08.ROC/GSE62564/EFS/ROC.pdf]

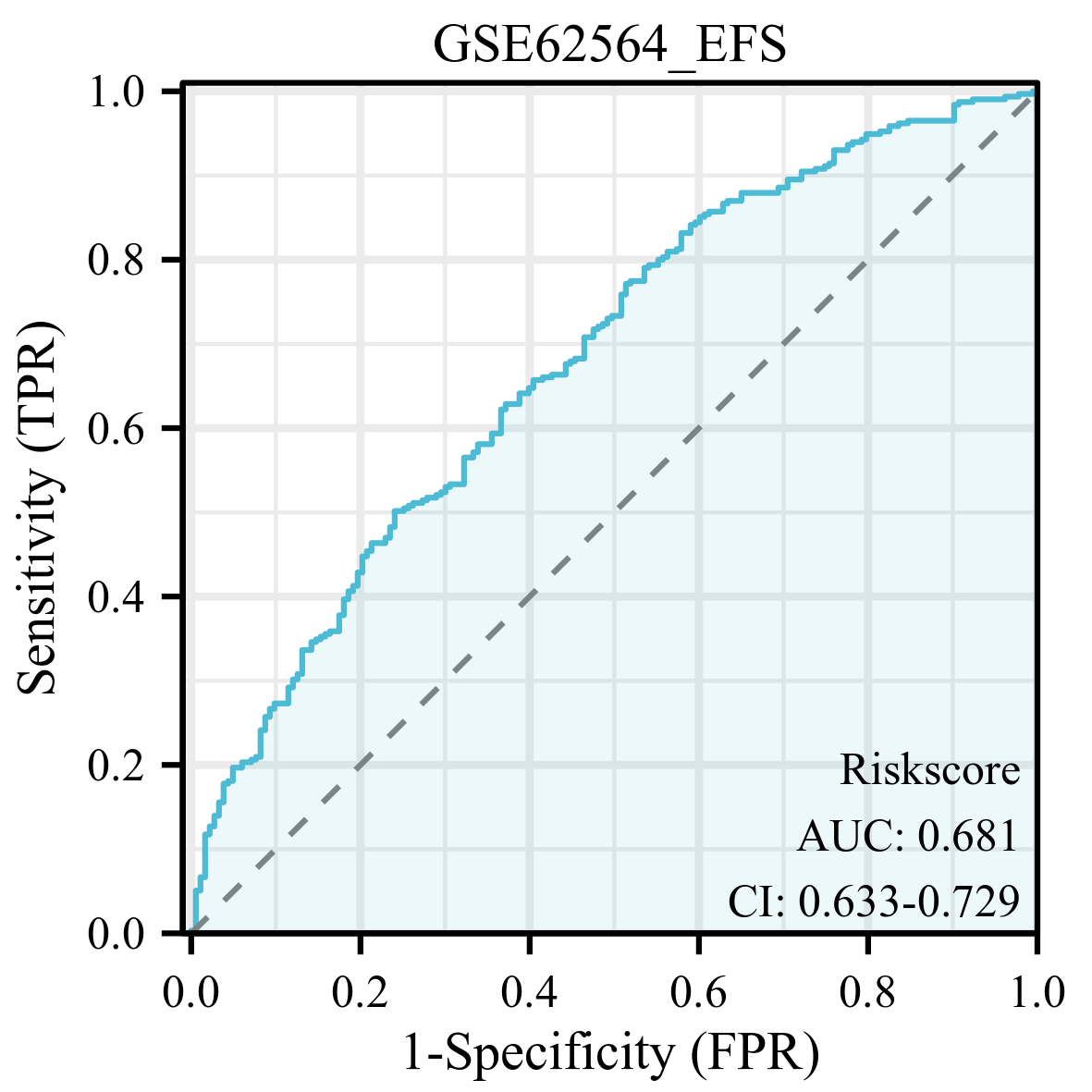

Supplement: Supplemental Information 6 [file peerj-13-19767-s006.zip › Raw Data/RNA-seq/08.ROC/GSE62564/EFS/ROC.tiff]

GSE62564\_OS

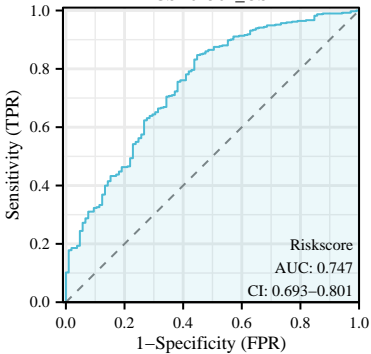

Supplement: Supplemental Information 6 [file peerj-13-19767-s006.zip › Raw Data/RNA-seq/08.ROC/GSE62564/OS/ROC.pdf]

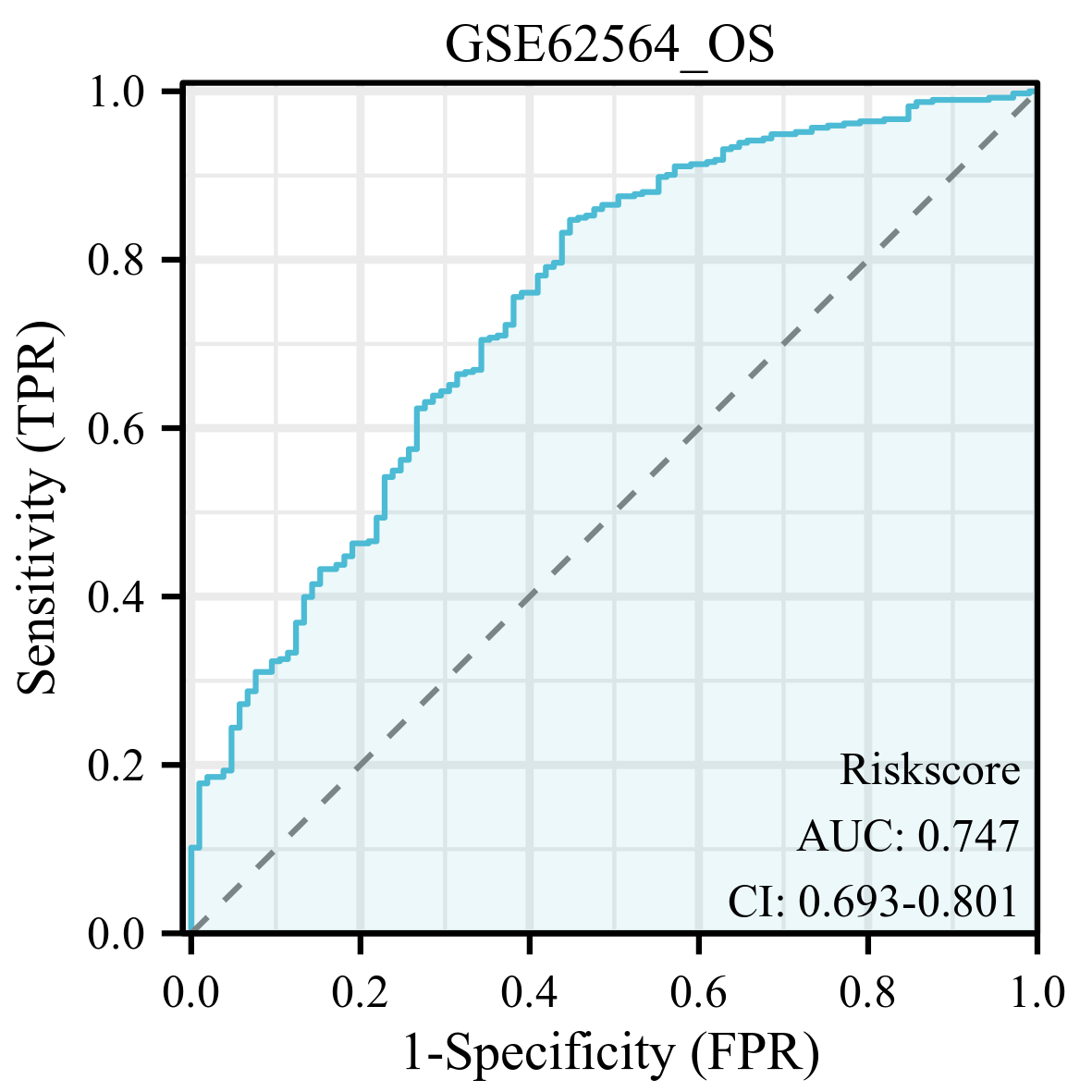

Supplement: Supplemental Information 6 [file peerj-13-19767-s006.zip › Raw Data/RNA-seq/08.ROC/GSE62564/OS/ROC.tiff]

GSE49710\_EFS

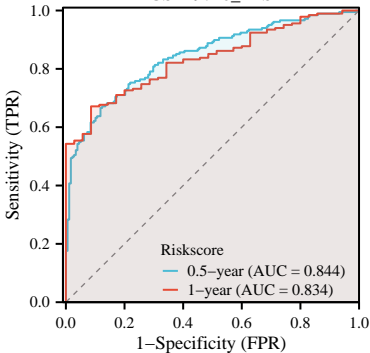

Supplement: Supplemental Information 6 [file peerj-13-19767-s006.zip › Raw Data/RNA-seq/09.time-indepROC/GSE49710/EFS/Time dependenceROC.pdf]

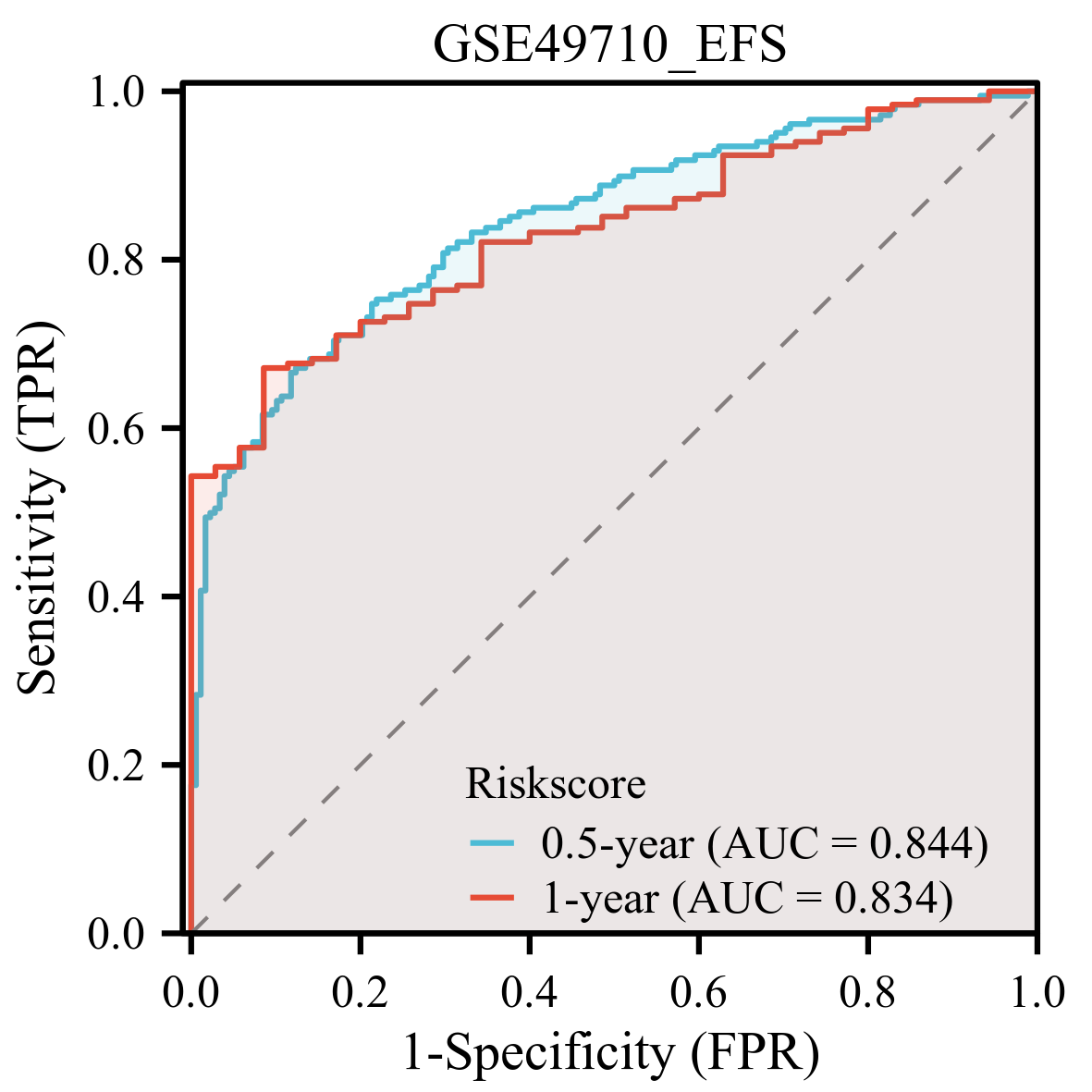

Supplement: Supplemental Information 6 [file peerj-13-19767-s006.zip › Raw Data/RNA-seq/09.time-indepROC/GSE49710/EFS/Time dependenceROC.tiff]

## GSE49710\_OS

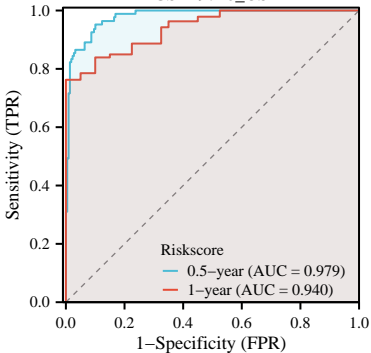

Supplement: Supplemental Information 6 [file peerj-13-19767-s006.zip › Raw Data/RNA-seq/09.time-indepROC/GSE49710/OS/Time dependenceROC.pdf]

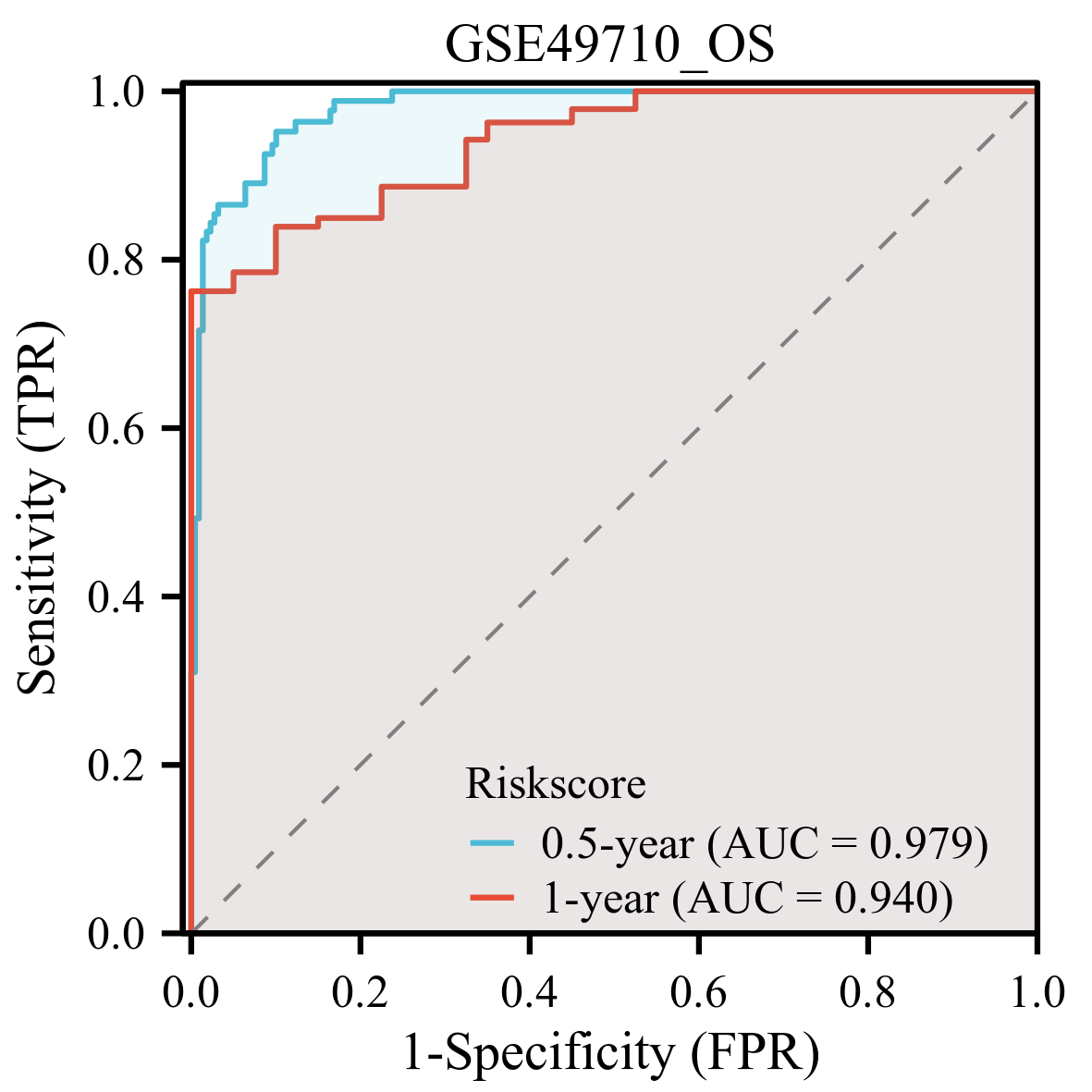

Supplement: Supplemental Information 6 [file peerj-13-19767-s006.zip › Raw Data/RNA-seq/09.time-indepROC/GSE49710/OS/Time dependenceROC.tiff]

GSE62564\_EFS

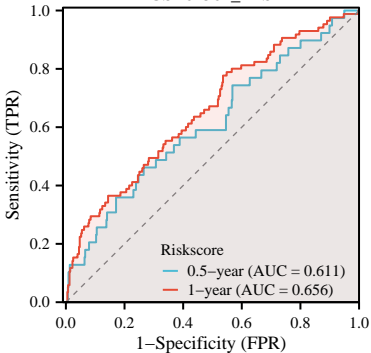

Supplement: Supplemental Information 6 [file peerj-13-19767-s006.zip › Raw Data/RNA-seq/09.time-indepROC/GSE62564/EFS/Time dependenceROC.pdf]

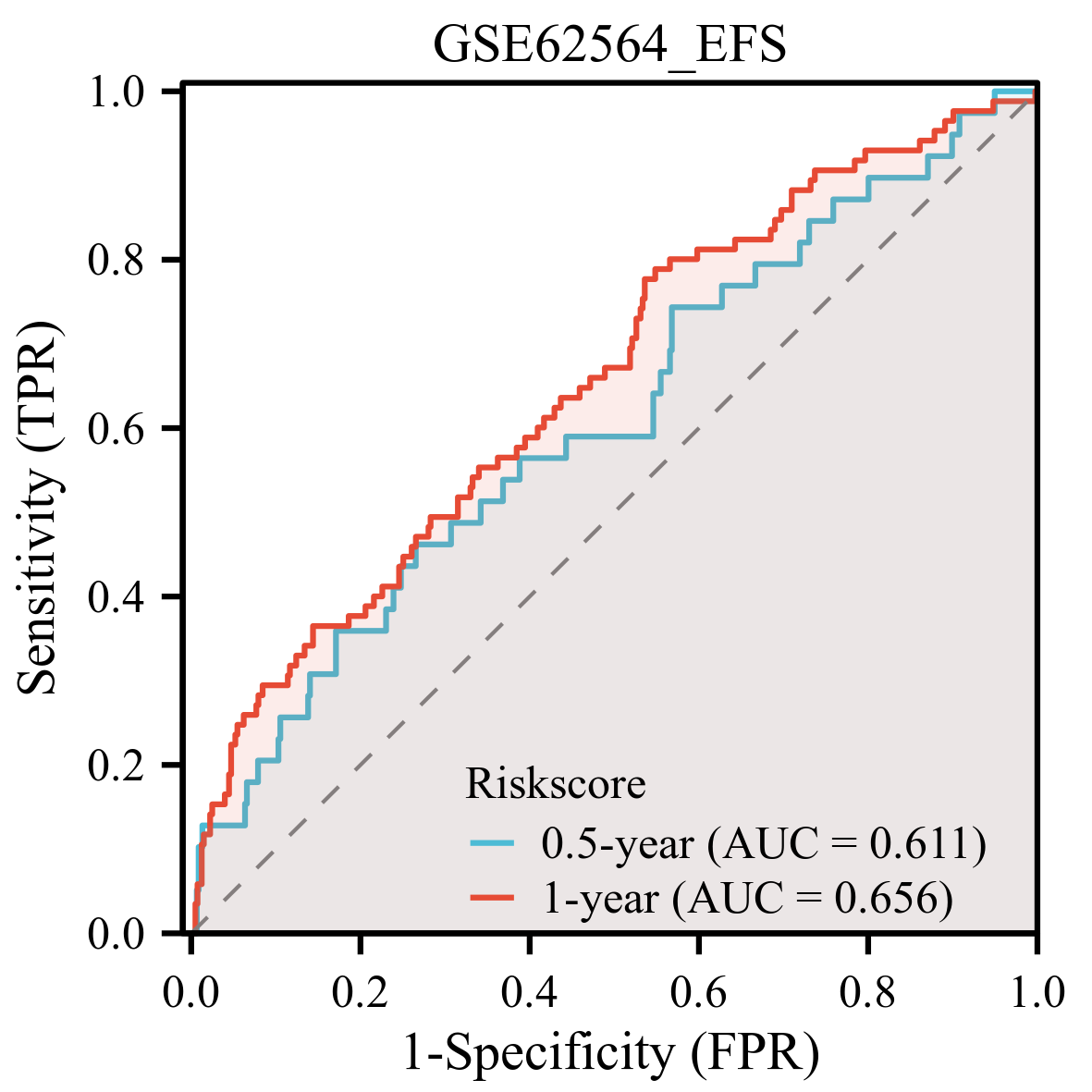

Supplement: Supplemental Information 6 [file peerj-13-19767-s006.zip › Raw Data/RNA-seq/09.time-indepROC/GSE62564/EFS/Time dependenceROC.tiff]

GSE62564\_OS

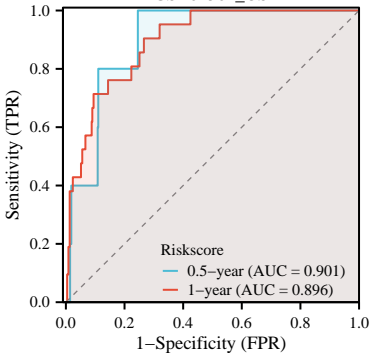

Supplement: Supplemental Information 6 [file peerj-13-19767-s006.zip › Raw Data/RNA-seq/09.time-indepROC/GSE62564/OS/Time dependenceOC.pdf]

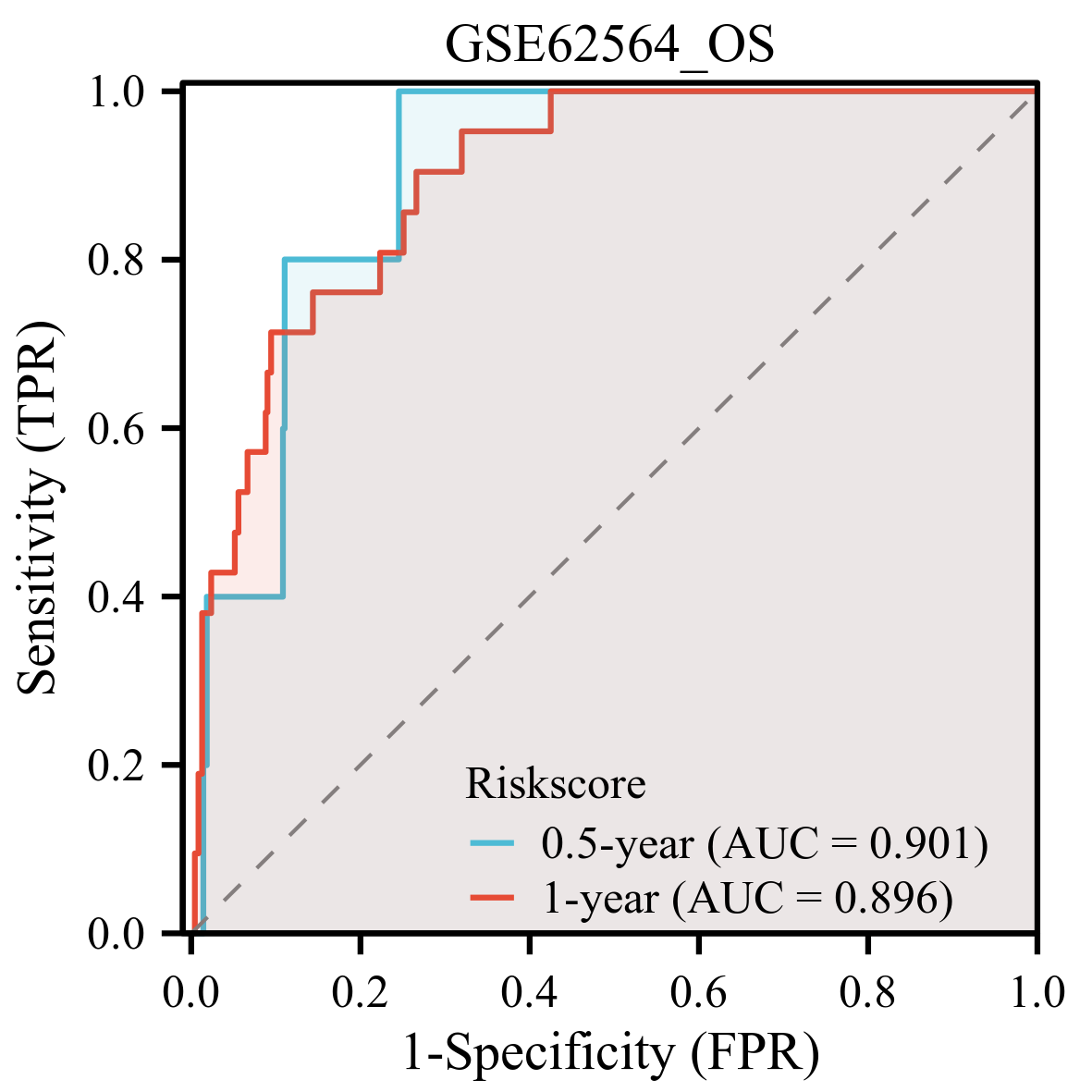

Supplement: Supplemental Information 6 [file peerj-13-19767-s006.zip › Raw Data/RNA-seq/09.time-indepROC/GSE62564/OS/Time dependenceROC.tiff]

GSE49710

Angiogenesis score

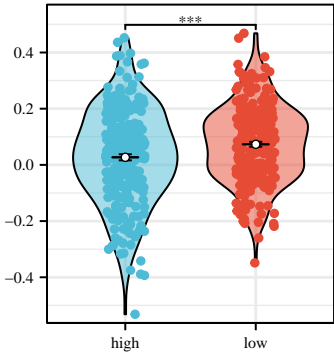

Supplement: Supplemental Information 6 [file peerj-13-19767-s006.zip › Raw Data/RNA-seq/12.score/GSE49710/Angiogenesis score/Group comparison chart.pdf]

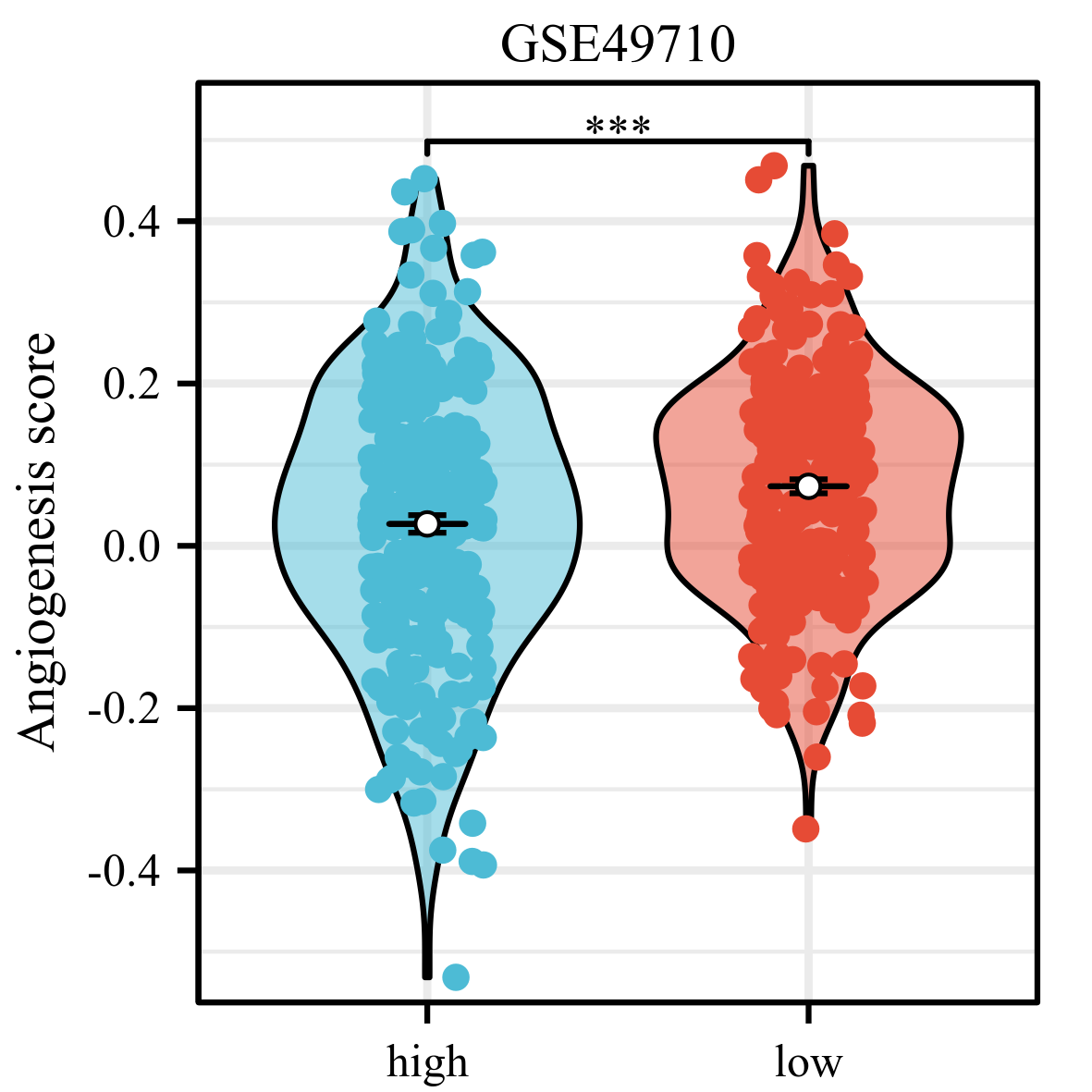

Supplement: Supplemental Information 6 [file peerj-13-19767-s006.zip › Raw Data/RNA-seq/12.score/GSE49710/Angiogenesis score/Group comparison chart.tiff]

GSE49710

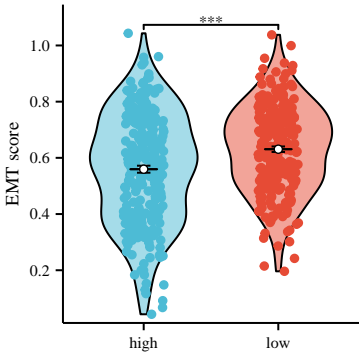

Supplement: Supplemental Information 6 [file peerj-13-19767-s006.zip › Raw Data/RNA-seq/12.score/GSE49710/EMT-score/Group comparison chart.pdf]

GSE49710

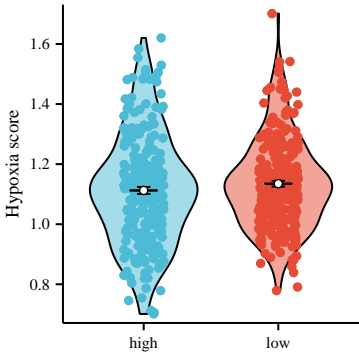

Supplement: Supplemental Information 6 [file peerj-13-19767-s006.zip › Raw Data/RNA-seq/12.score/GSE49710/Hypoxia score/Group comparison chart.pdf]

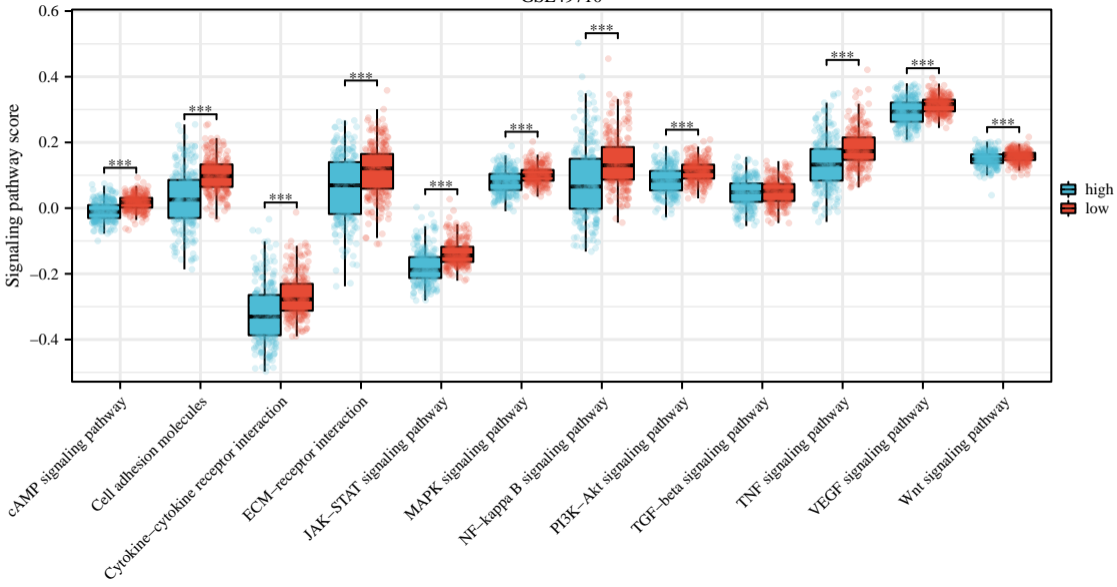

Supplement: Supplemental Information 6 [file peerj-13-19767-s006.zip › Raw Data/RNA-seq/12.score/GSE49710/Scores related to signal transduction pathways/Group comparison chart.pdf]

## GSE49710

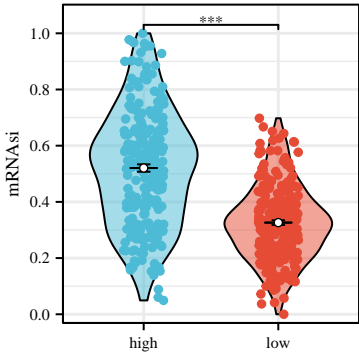

Supplement: Supplemental Information 6 [file peerj-13-19767-s006.zip › Raw Data/RNA-seq/12.score/GSE49710/Tumor stemness score/Group comparison chart.pdf]

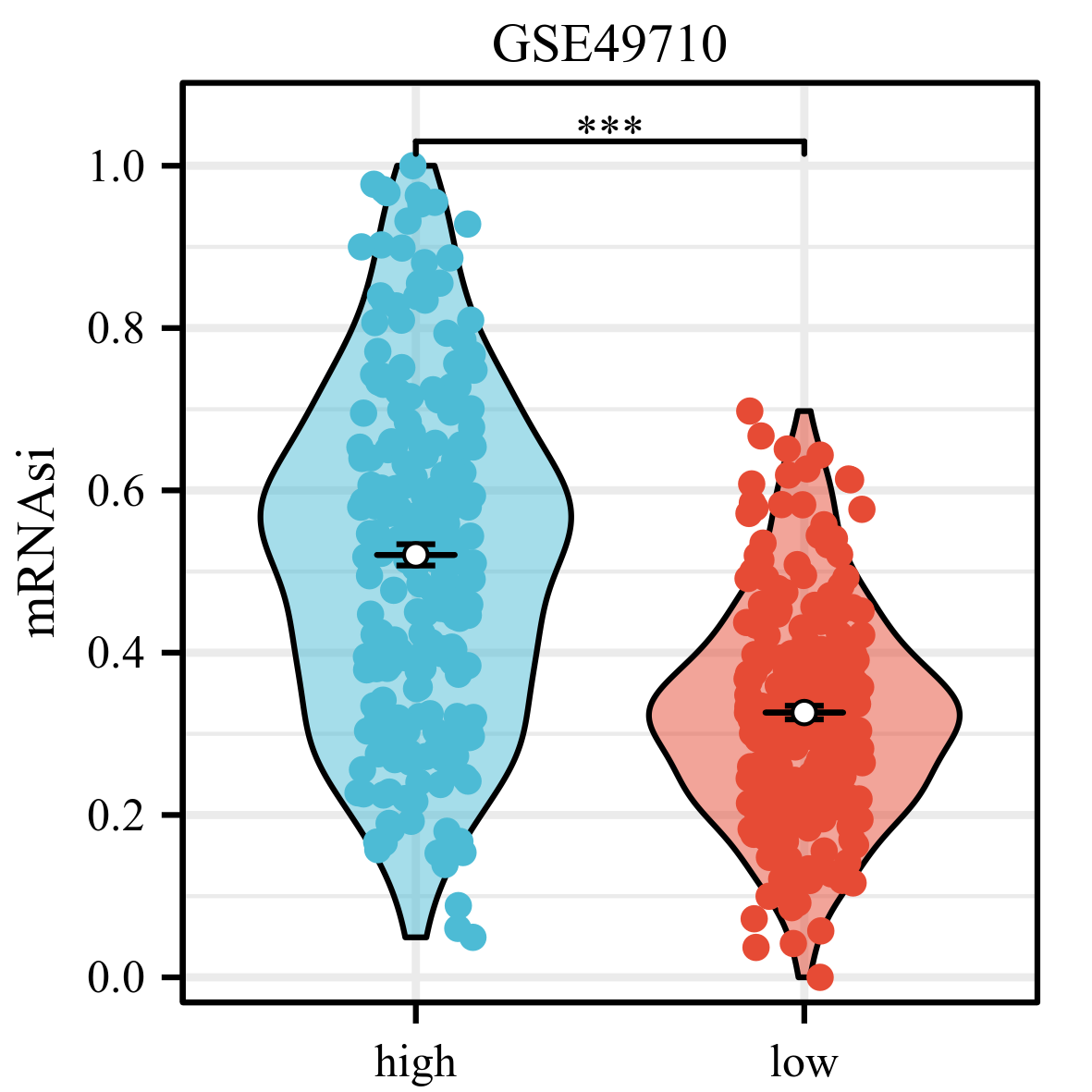

Supplement: Supplemental Information 6 [file peerj-13-19767-s006.zip › Raw Data/RNA-seq/12.score/GSE49710/Tumor stemness score/Group comparison chart.tiff]

## GSE62564

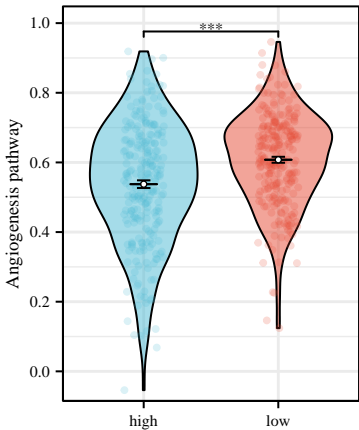

Supplement: Supplemental Information 6 [file peerj-13-19767-s006.zip › Raw Data/RNA-seq/12.score/GSE62564/Angiogenesis score/Group comparison chart.pdf]

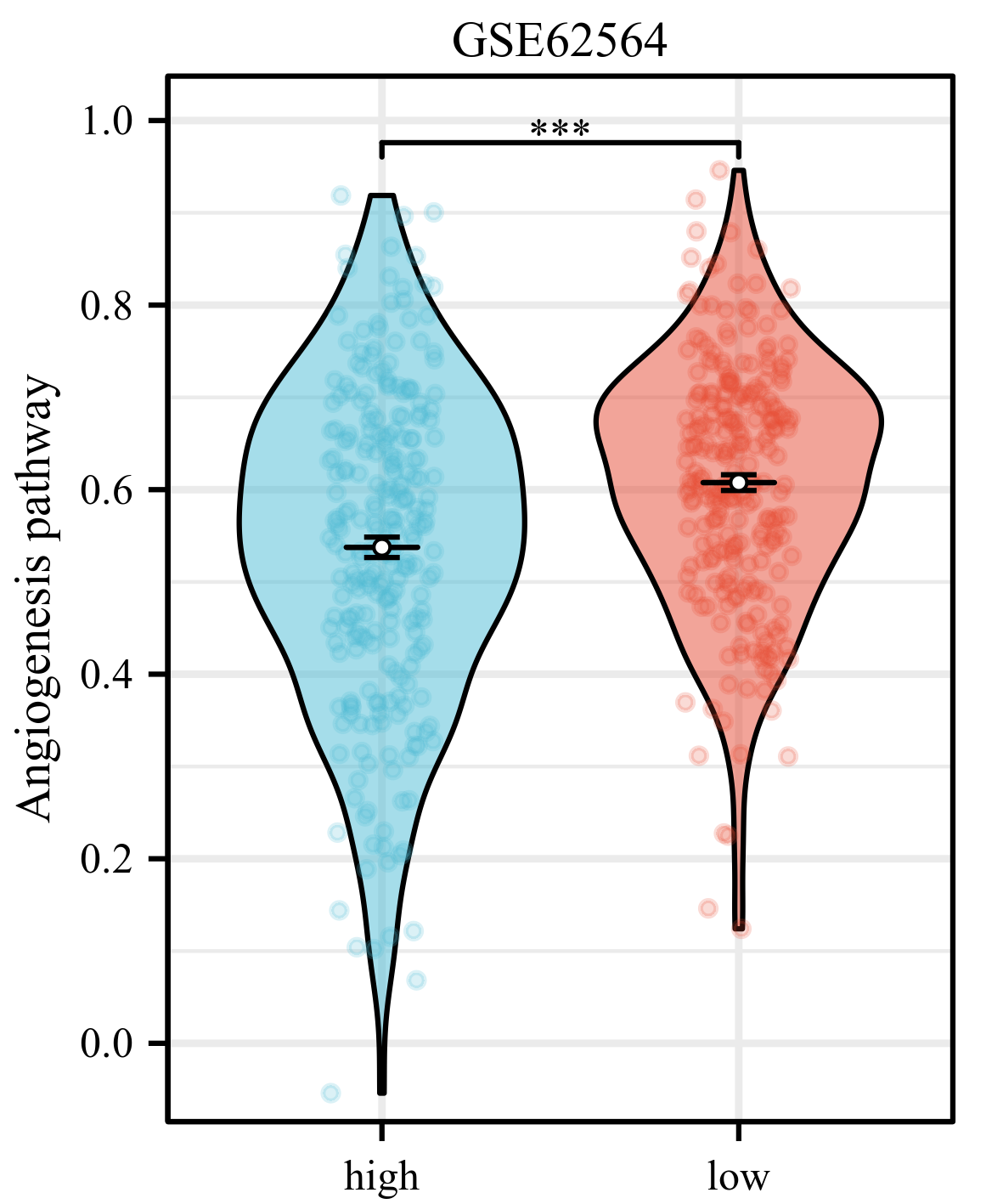

Supplement: Supplemental Information 6 [file peerj-13-19767-s006.zip › Raw Data/RNA-seq/12.score/GSE62564/Angiogenesis score/Group comparison chart.tiff]

# GSE62564

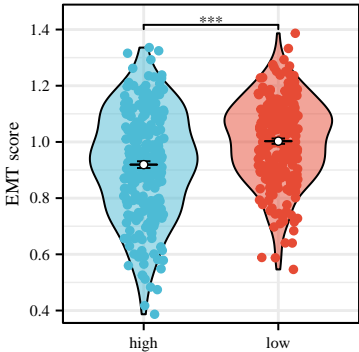

Supplement: Supplemental Information 6 [file peerj-13-19767-s006.zip › Raw Data/RNA-seq/12.score/GSE62564/EMT score/Group comparison chart.pdf]

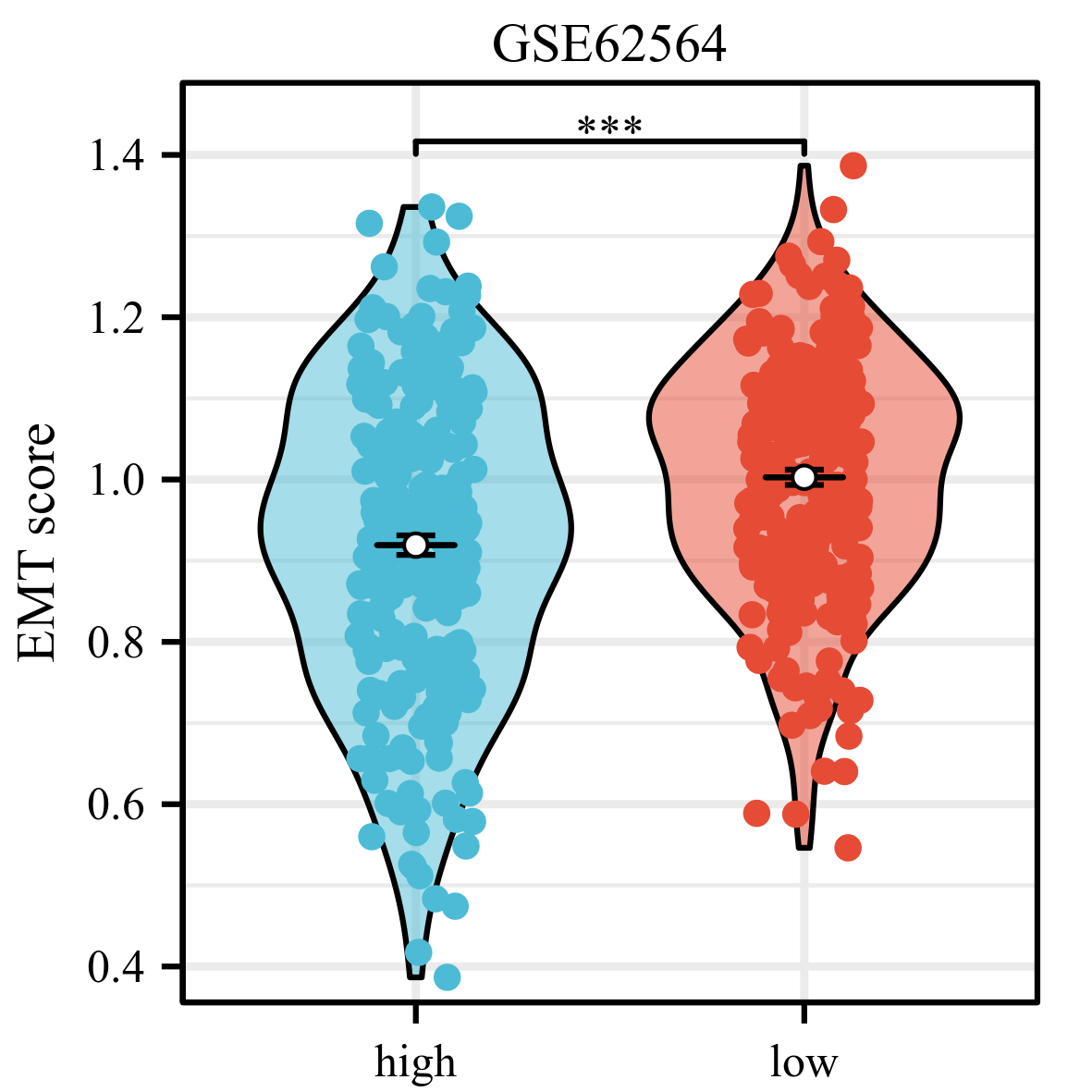

Supplement: Supplemental Information 6 [file peerj-13-19767-s006.zip › Raw Data/RNA-seq/12.score/GSE62564/EMT score/Group comparison chart.tiff]

GSE62564

Hypoxia score

2.2

2.0

1.8

1.6

1.4

high

low

\*\*\*

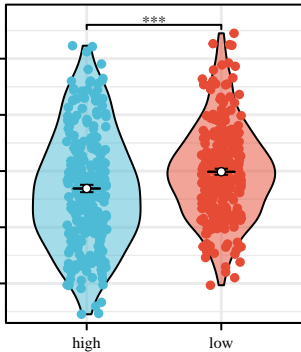

Supplement: Supplemental Information 6 [file peerj-13-19767-s006.zip › Raw Data/RNA-seq/12.score/GSE62564/Hypoxia score/Group comparison chart.pdf]

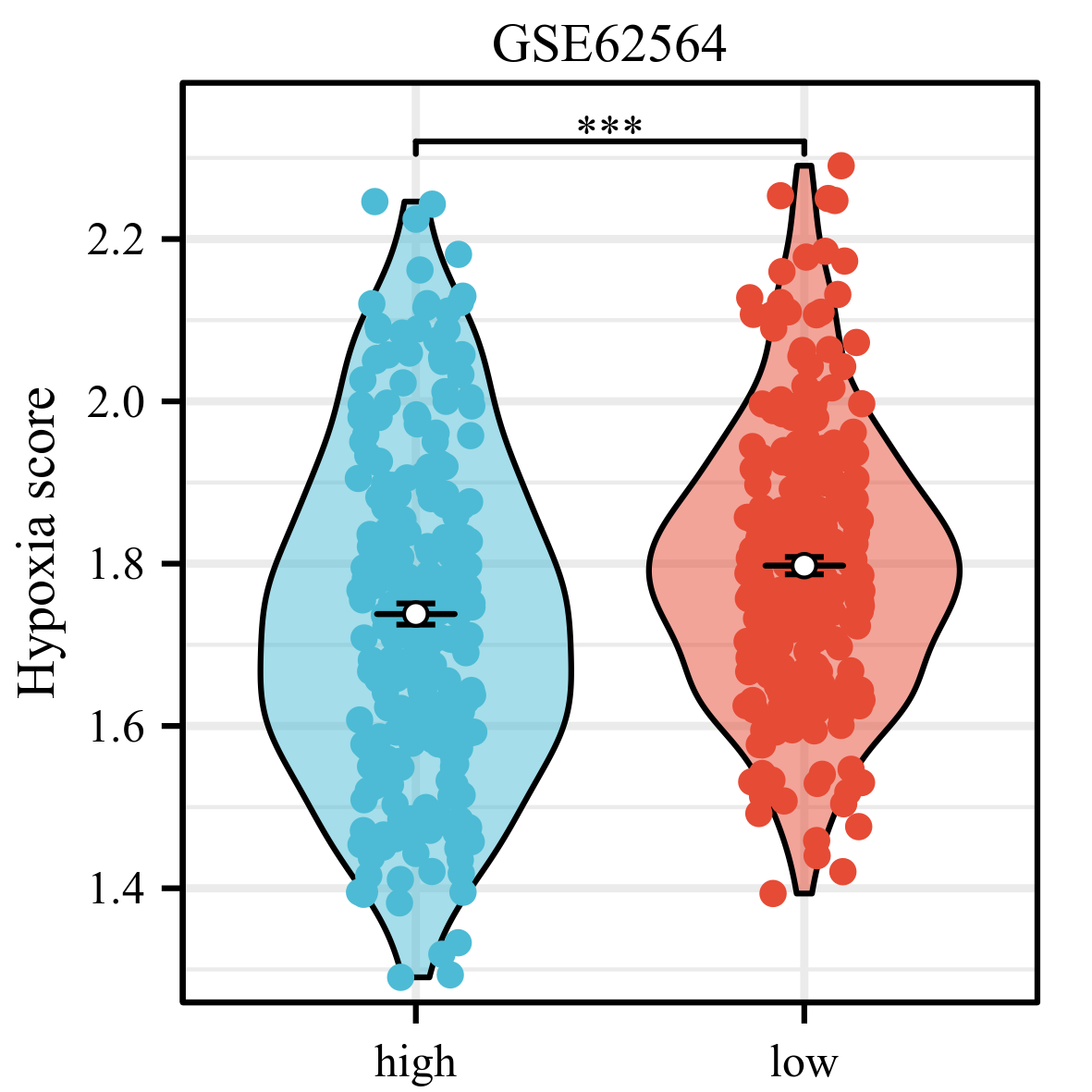

Supplement: Supplemental Information 6 [file peerj-13-19767-s006.zip › Raw Data/RNA-seq/12.score/GSE62564/Hypoxia score/Group comparison chart.tiff]

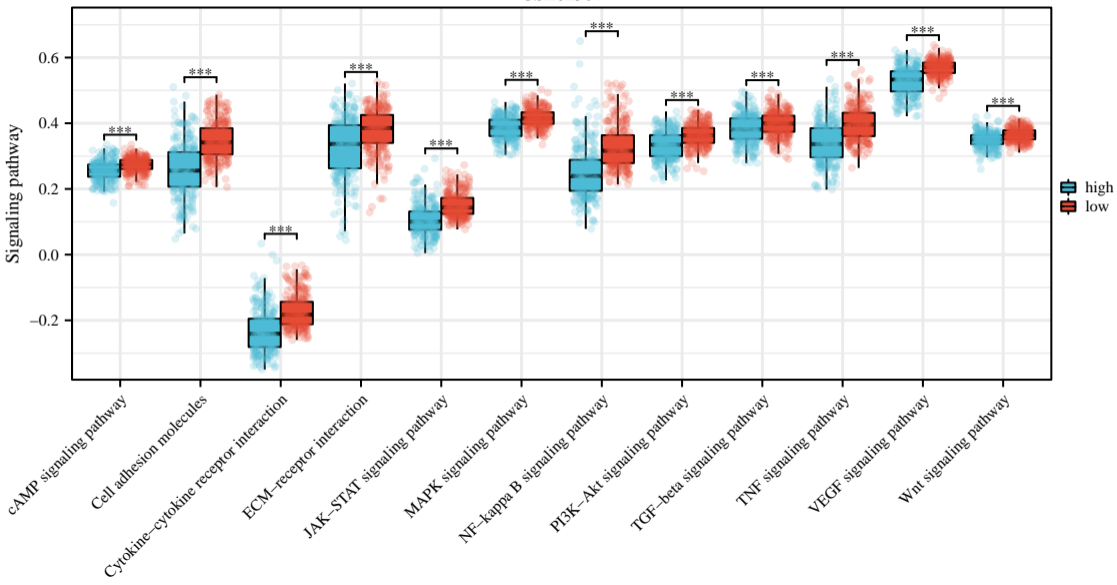

Supplement: Supplemental Information 6 [file peerj-13-19767-s006.zip › Raw Data/RNA-seq/12.score/GSE62564/Scores related to signal transduction pathways/Group comparison chart.pdf]

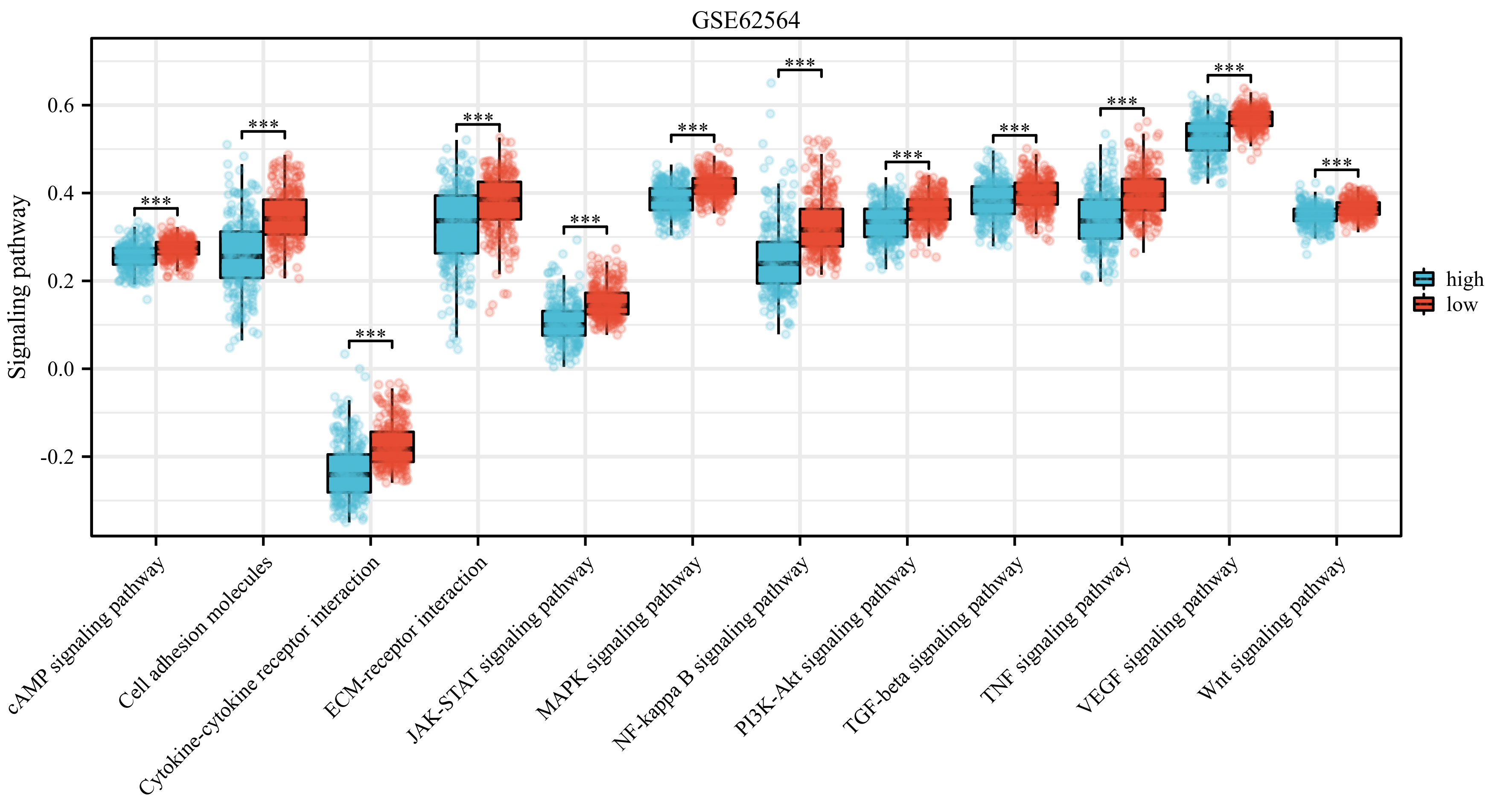

Supplement: Supplemental Information 6 [file peerj-13-19767-s006.zip › Raw Data/RNA-seq/12.score/GSE62564/Scores related to signal transduction pathways/Group comparison chart.tiff]

# GSE62564

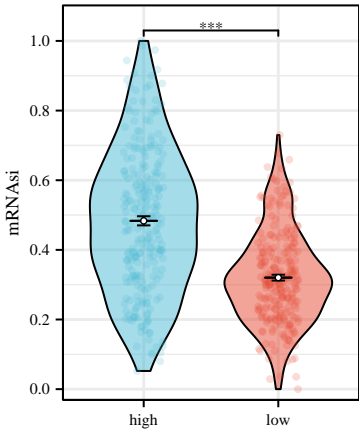

Supplement: Supplemental Information 6 [file peerj-13-19767-s006.zip › Raw Data/RNA-seq/12.score/GSE62564/Tumor stemness score/Group comparison chart.pdf]

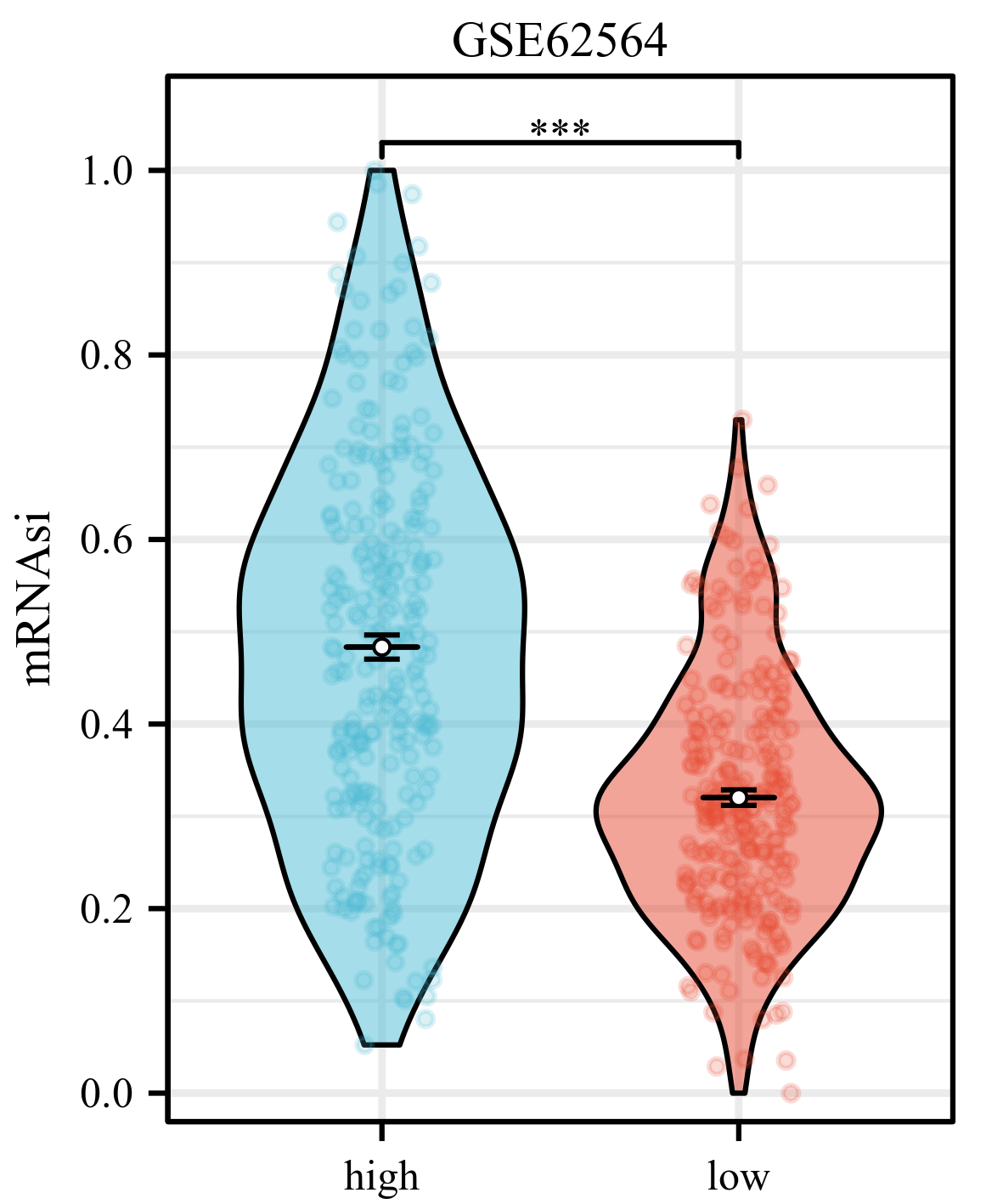

Supplement: Supplemental Information 6 [file peerj-13-19767-s006.zip › Raw Data/RNA-seq/12.score/GSE62564/Tumor stemness score/Group comparison chart.tiff]

## GSE49710

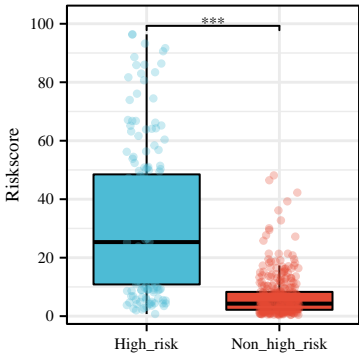

Supplement: Supplemental Information 6 [file peerj-13-19767-s006.zip › Raw Data/RNA-seq/15.Clinical-correlation/Clinical_risk.pdf]

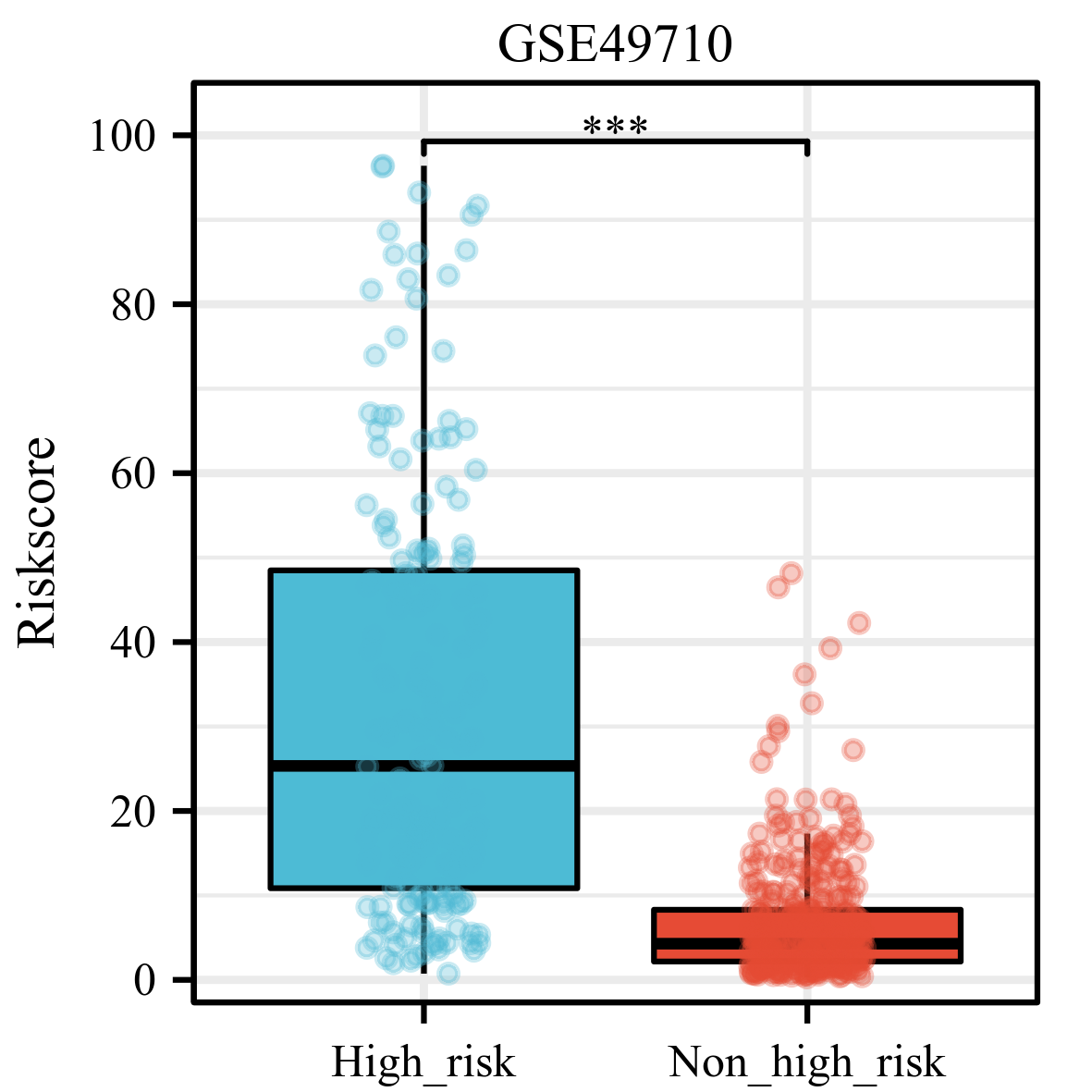

Supplement: Supplemental Information 6 [file peerj-13-19767-s006.zip › Raw Data/RNA-seq/15.Clinical-correlation/Clinical_risk.tiff]

## GSE49710

Risk score

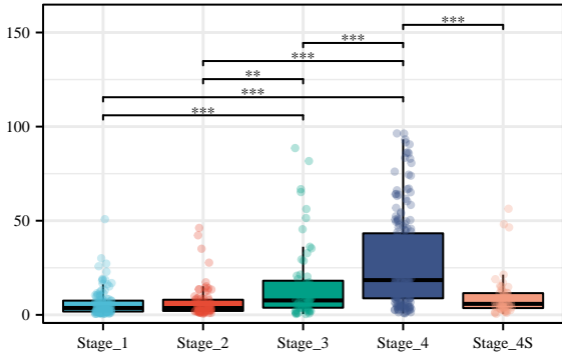

Supplement: Supplemental Information 6 [file peerj-13-19767-s006.zip › Raw Data/RNA-seq/15.Clinical-correlation/INSS_stage.pdf]

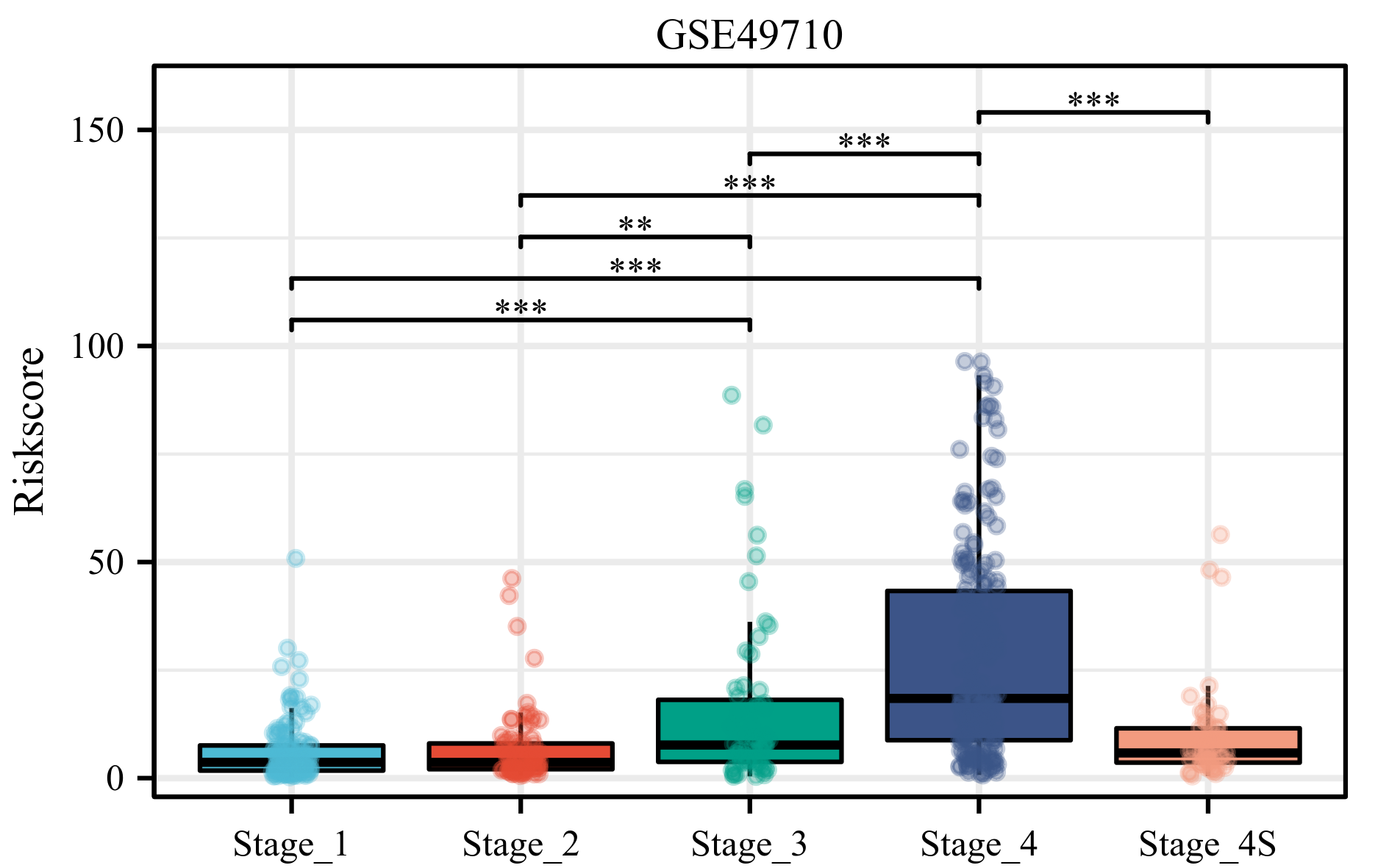

Supplement: Supplemental Information 6 [file peerj-13-19767-s006.zip › Raw Data/RNA-seq/15.Clinical-correlation/INSS_stage.tiff]

## GSE49710

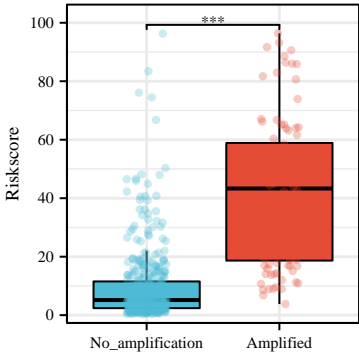

Supplement: Supplemental Information 6 [file peerj-13-19767-s006.zip › Raw Data/RNA-seq/15.Clinical-correlation/Mycn_status.pdf]

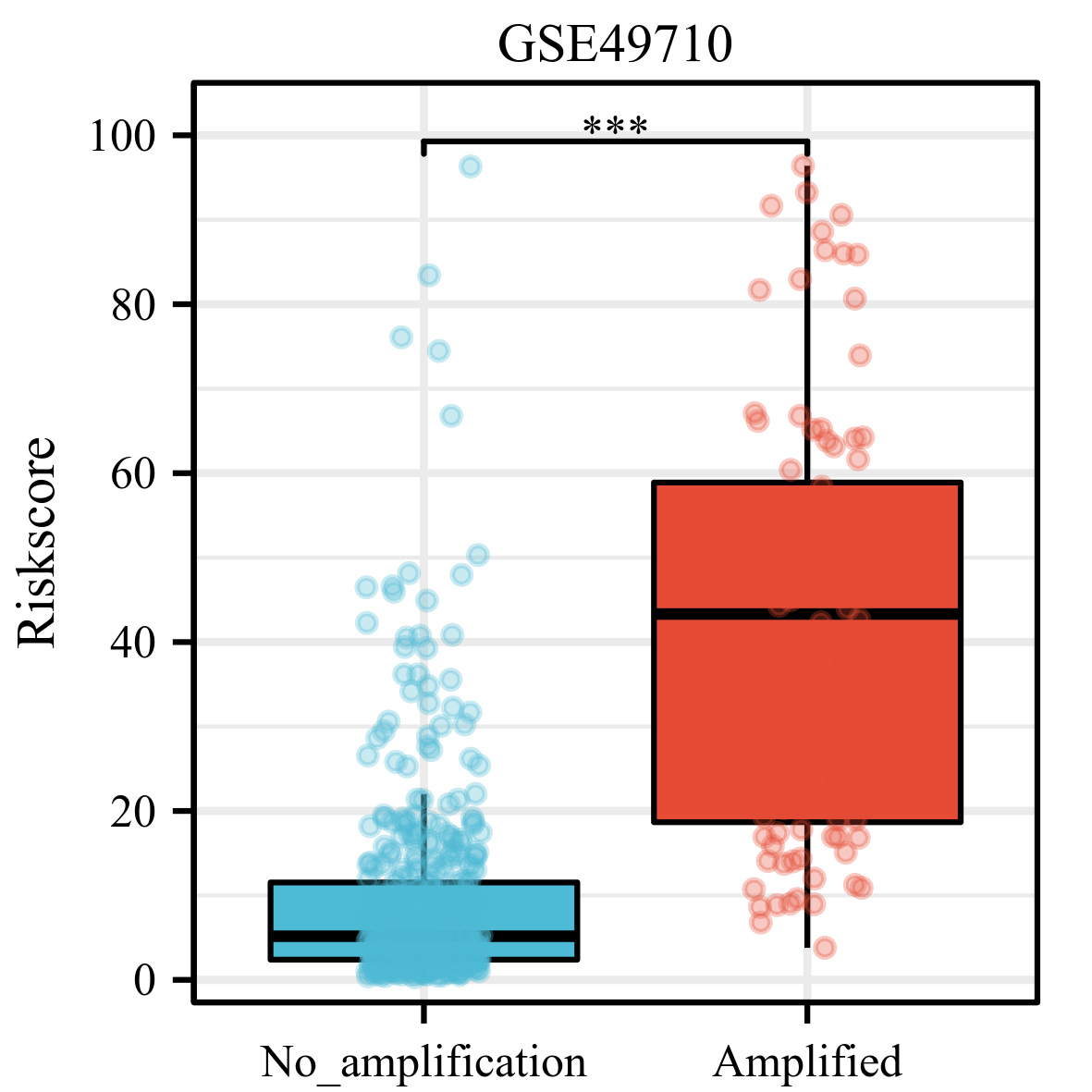

Supplement: Supplemental Information 6 [file peerj-13-19767-s006.zip › Raw Data/RNA-seq/15.Clinical-correlation/Mycn_status.tiff]

## GSE49710

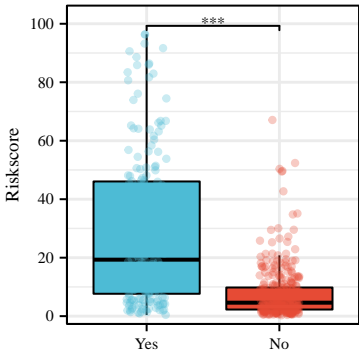

Supplement: Supplemental Information 6 [file peerj-13-19767-s006.zip › Raw Data/RNA-seq/15.Clinical-correlation/Progression.pdf]

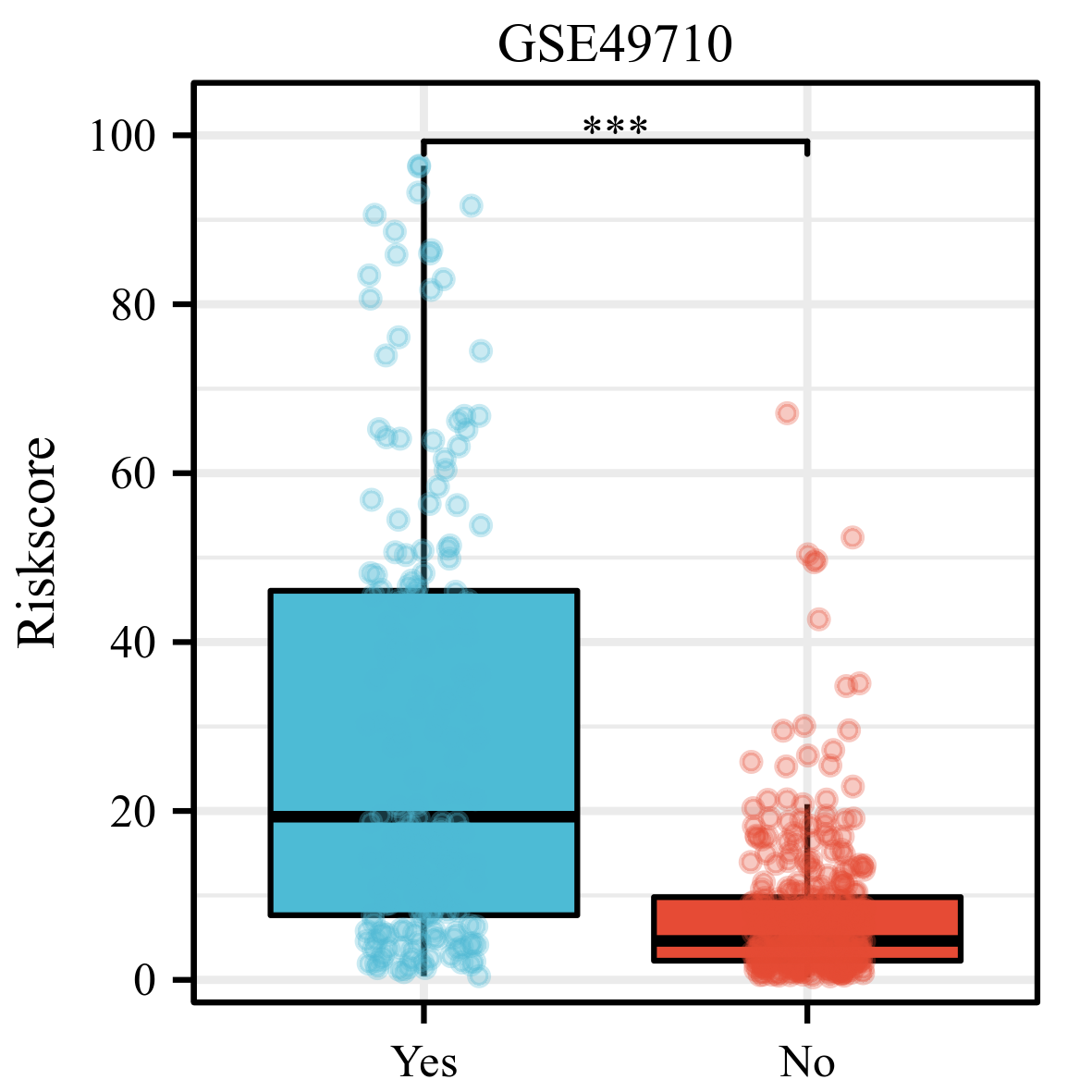

Supplement: Supplemental Information 6 [file peerj-13-19767-s006.zip › Raw Data/RNA-seq/15.Clinical-correlation/Progression.tiff]

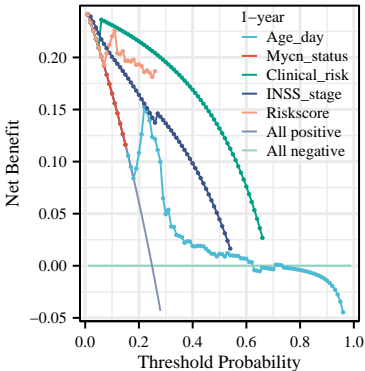

Supplement: Supplemental Information 6 [file peerj-13-19767-s006.zip › Raw Data/RNA-seq/17.nom/DCA/Prognosis DCA.pdf]

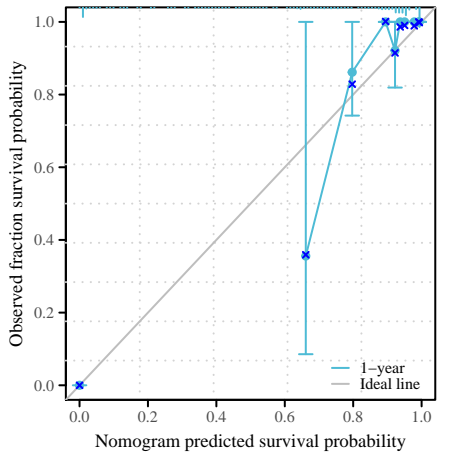

Supplement: Supplemental Information 6 [file peerj-13-19767-s006.zip › Raw Data/RNA-seq/17.nom/Prognostic calibration curve/output/Prognostic calibration curve.pdf]

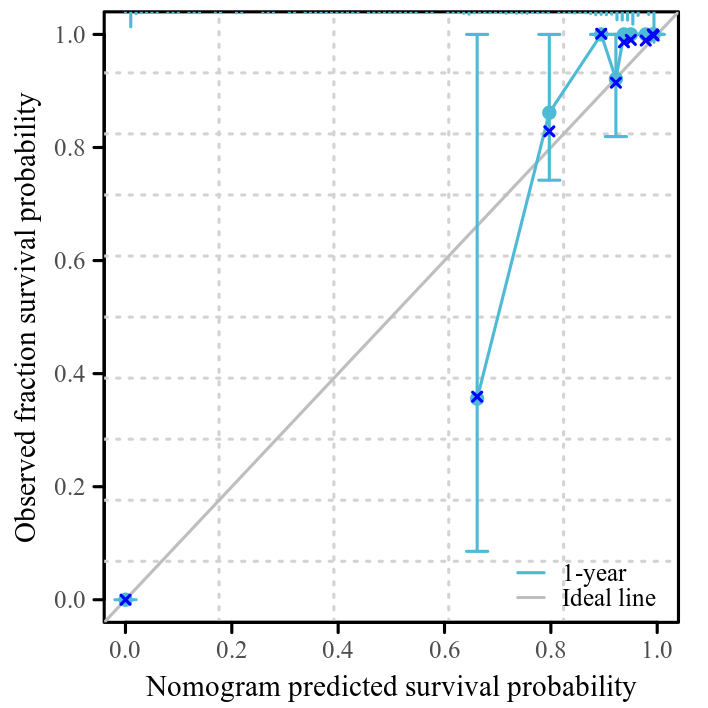

Supplement: Supplemental Information 6 [file peerj-13-19767-s006.zip › Raw Data/RNA-seq/17.nom/Prognostic calibration curve/output/Prognostic calibration curve.png]

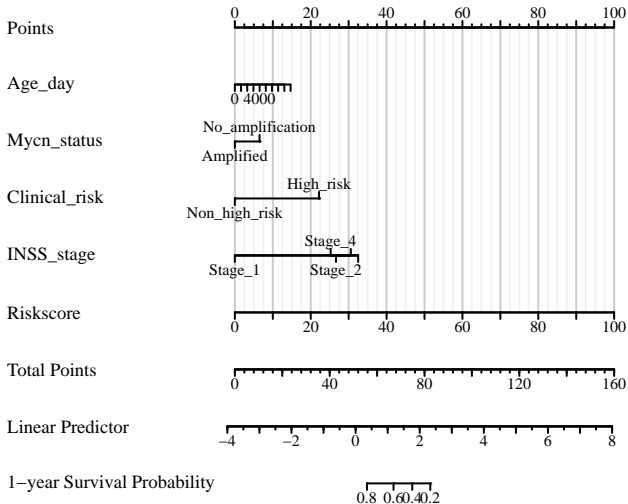

Supplement: Supplemental Information 6 [file peerj-13-19767-s006.zip › Raw Data/RNA-seq/17.nom/Prognostic nomogram/Prognostic nomogram.pdf]

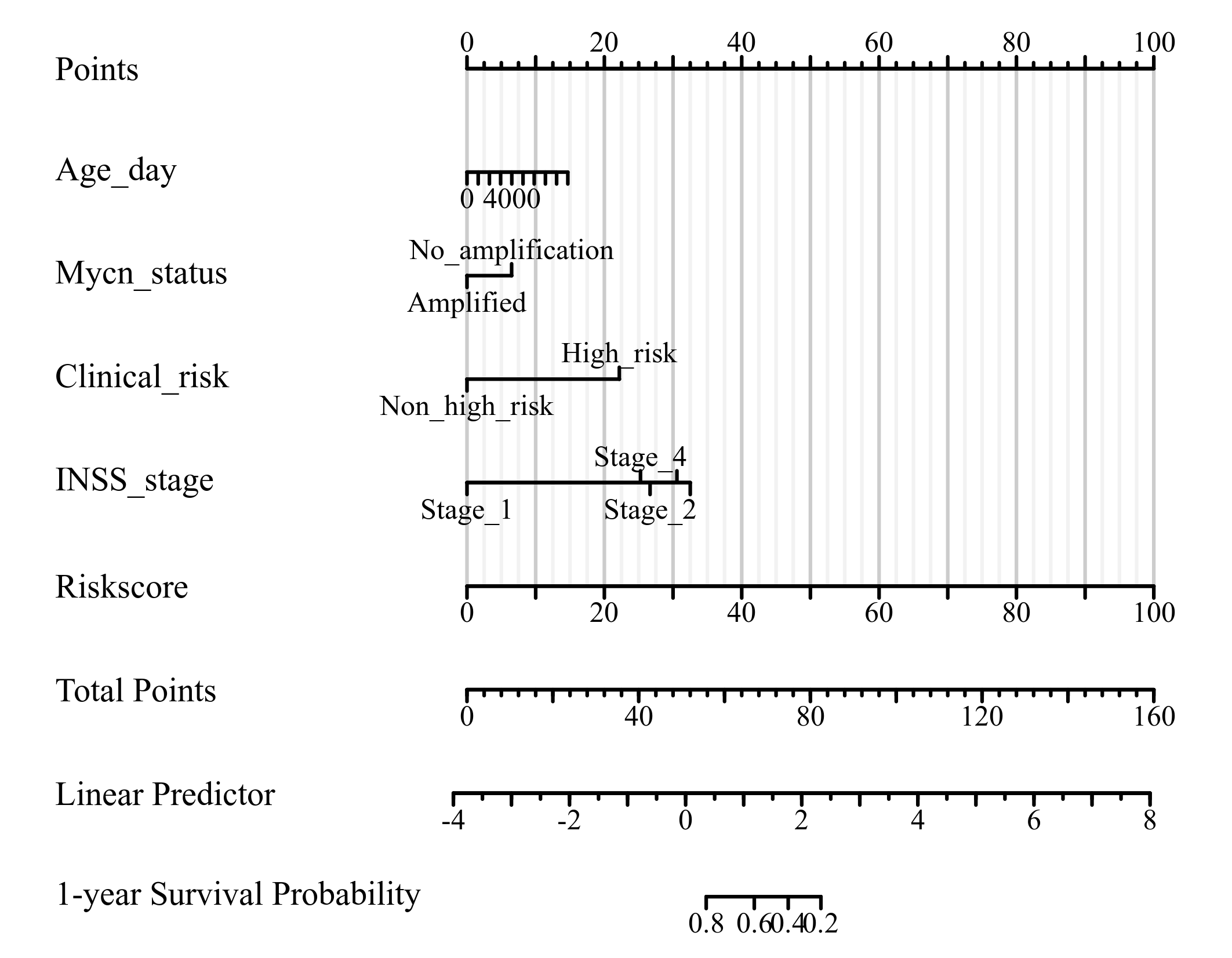

Supplement: Supplemental Information 6 [file peerj-13-19767-s006.zip › Raw Data/RNA-seq/17.nom/Prognostic nomogram/Prognostic nomogram.tiff]

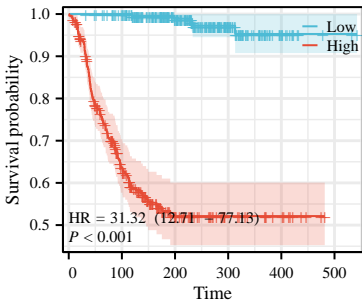

|      |     |     |     |    |    |   |
|------|-----|-----|-----|----|----|---|
| Low  | 246 | 232 | 150 | 61 | 14 | 2 |
| High | 247 | 122 | 41  | 17 | 9  | 0 |

Supplement: Supplemental Information 6 [file peerj-13-19767-s006.zip › Raw Data/RNA-seq/17.nom/Survival analysis/Survival curve graph.pdf]

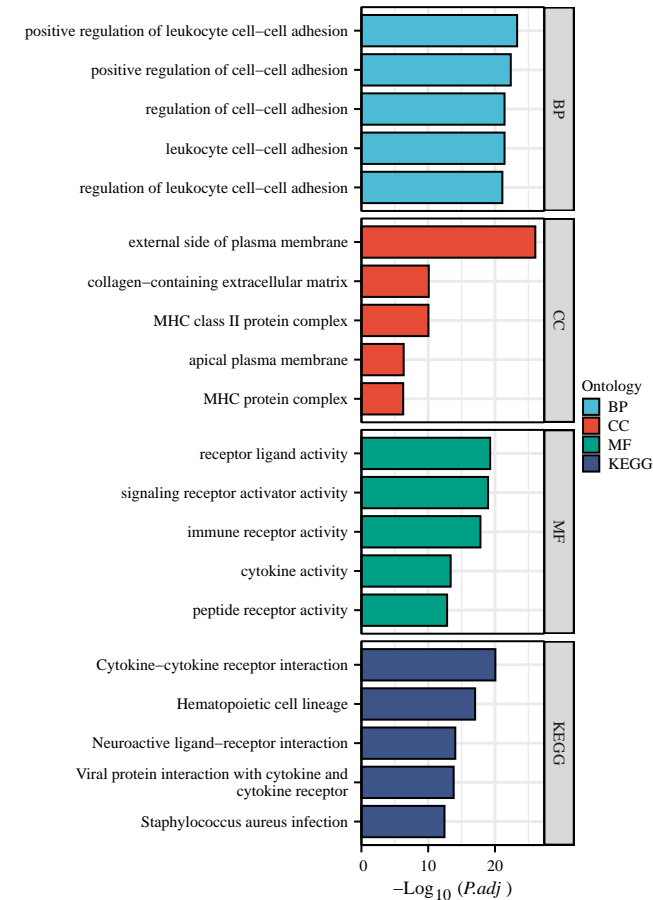

Supplement: Supplemental Information 6 [file peerj-13-19767-s006.zip › Raw Data/RNA-seq/18.rish-group-KEGG/GOKEGG.pdf]

risk High Low

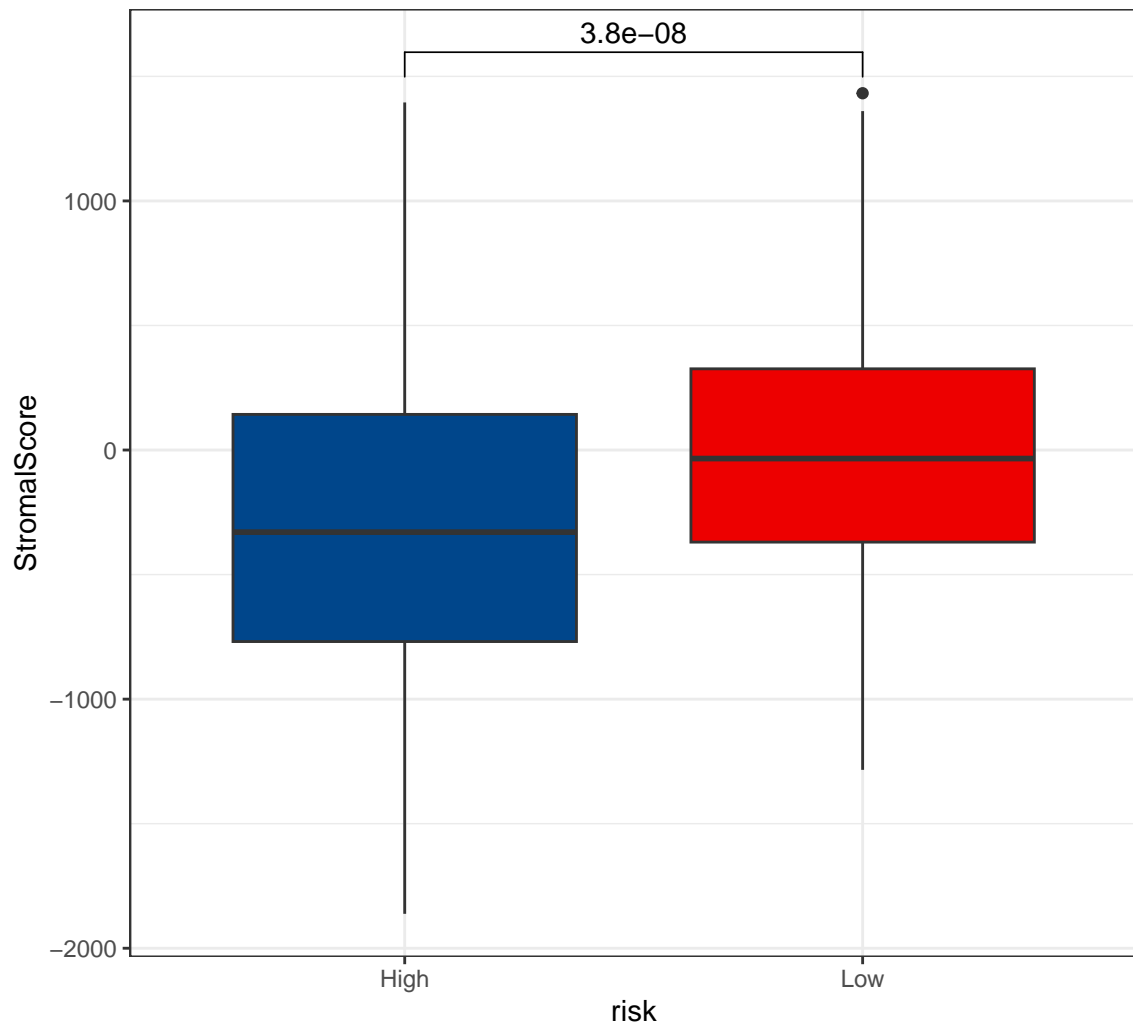

Supplement: Supplemental Information 6 [file peerj-13-19767-s006.zip › Raw Data/RNA-seq/19.TIP/results/fig9a1.pdf]

risk High Low

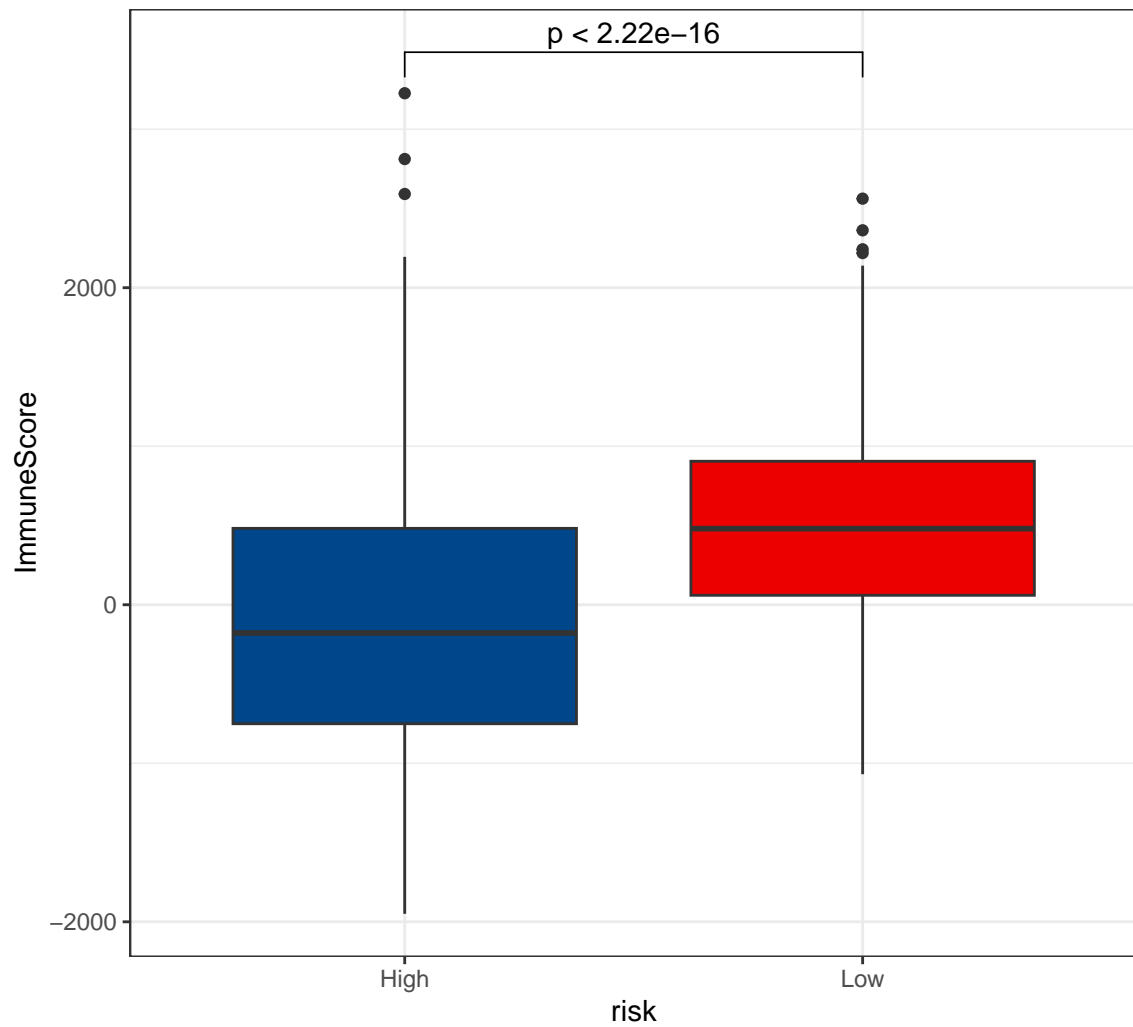

Supplement: Supplemental Information 6 [file peerj-13-19767-s006.zip › Raw Data/RNA-seq/19.TIP/results/fig9a2.pdf]

risk High Low

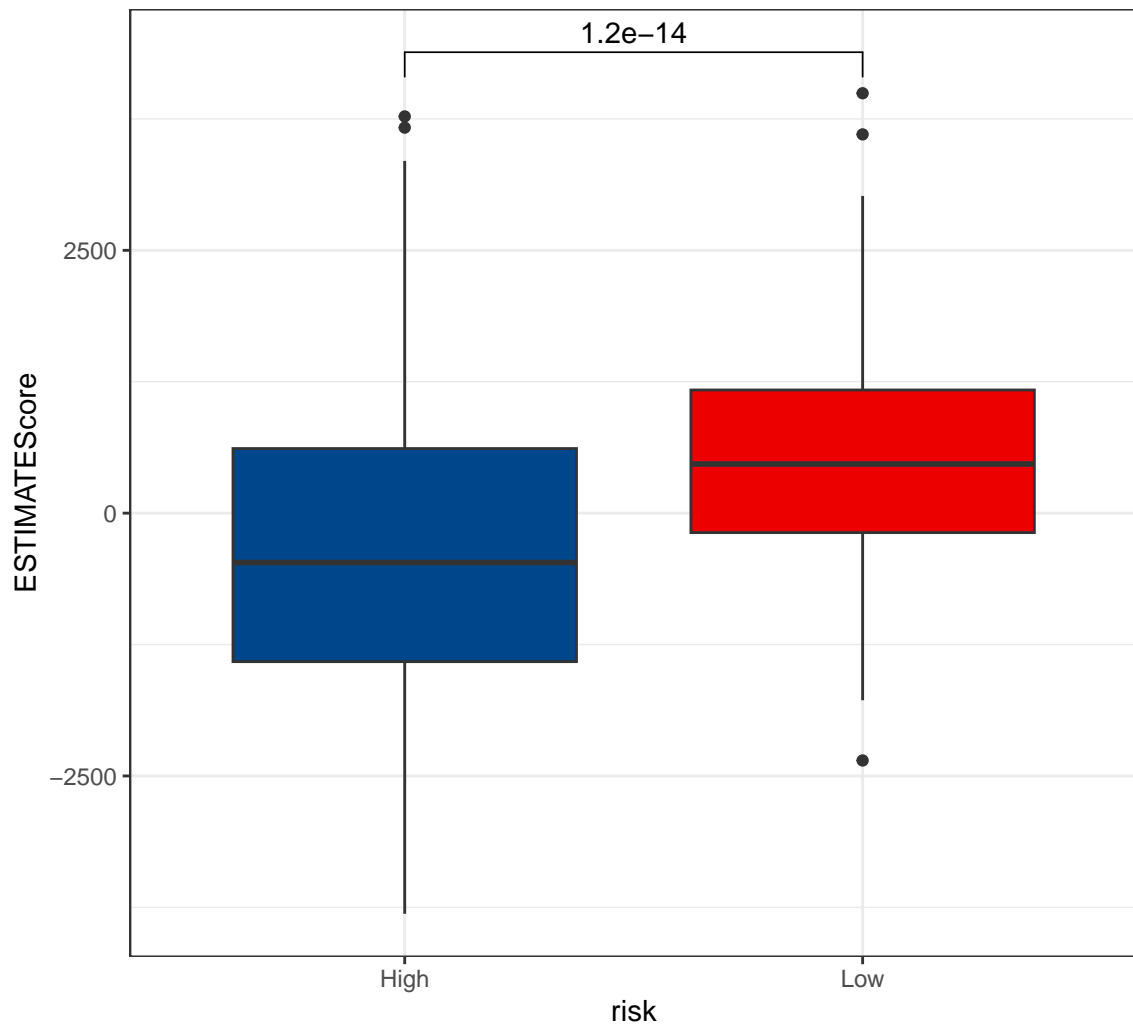

Supplement: Supplemental Information 6 [file peerj-13-19767-s006.zip › Raw Data/RNA-seq/19.TIP/results/fig9a3.pdf]

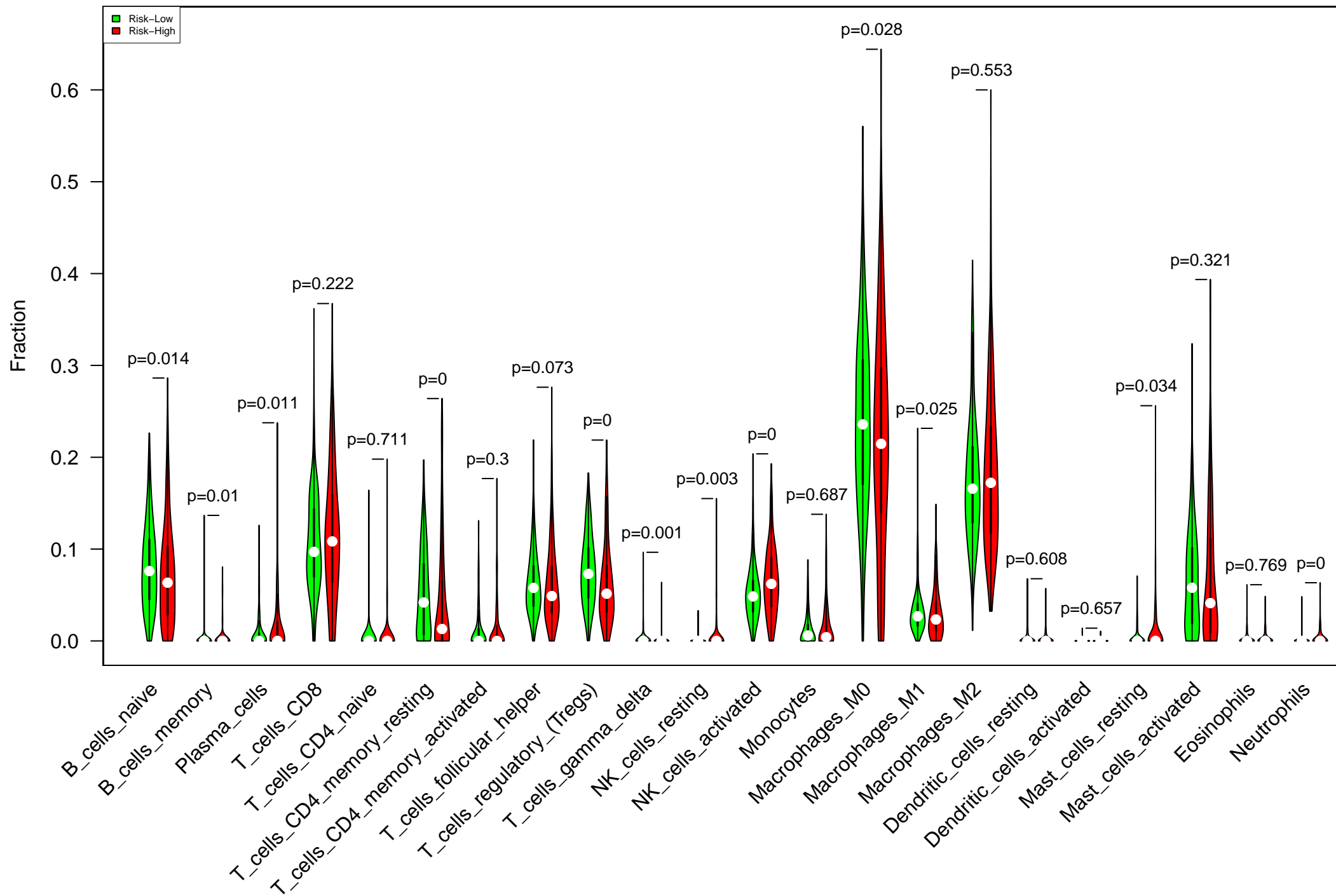

Supplement: Supplemental Information 6 [file peerj-13-19767-s006.zip › Raw Data/RNA-seq/19.TIP/results/fig9c.pdf]

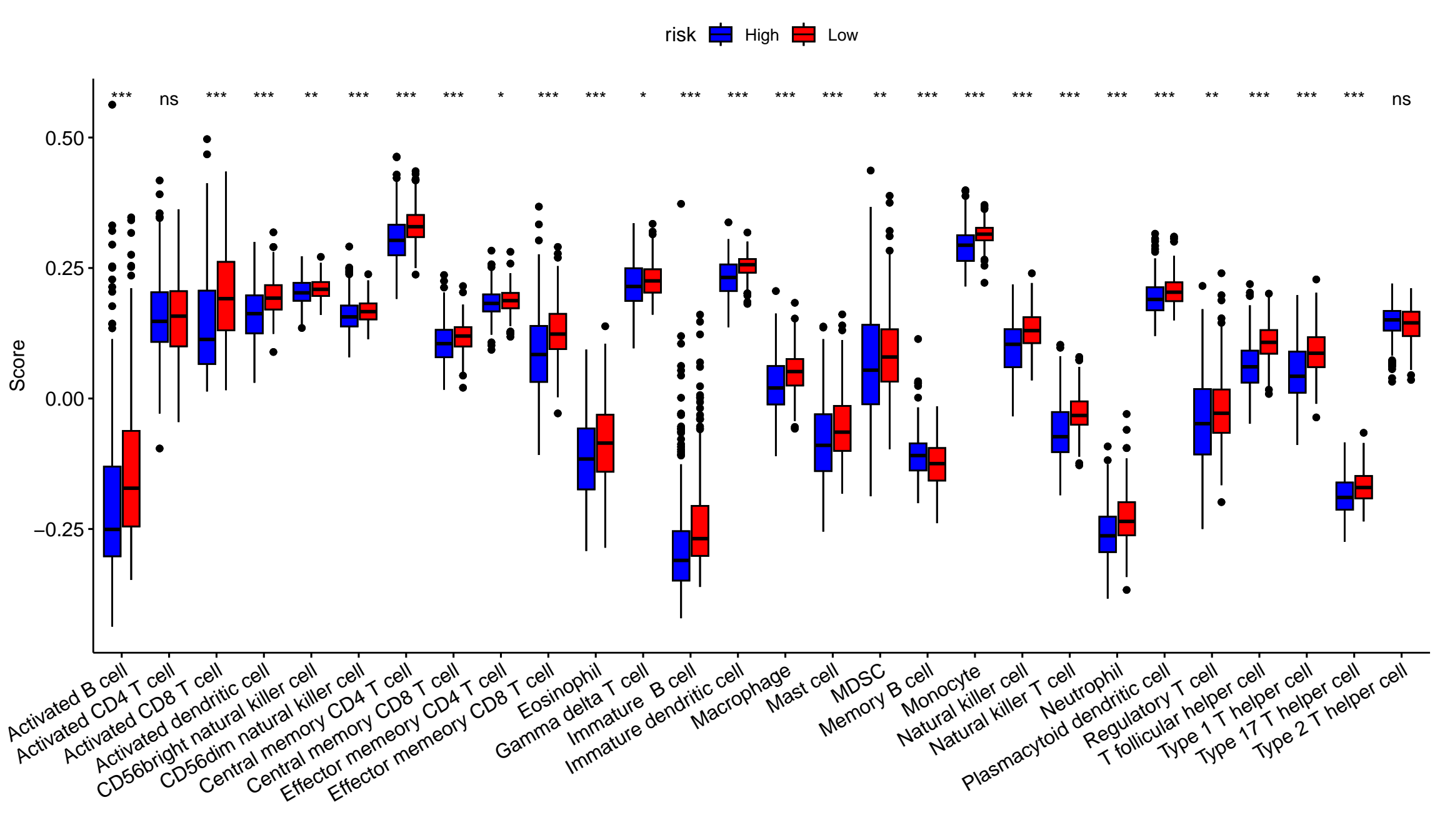

Supplement: Supplemental Information 6 [file peerj-13-19767-s006.zip › Raw Data/RNA-seq/19.TIP/results/fig9d.pdf]

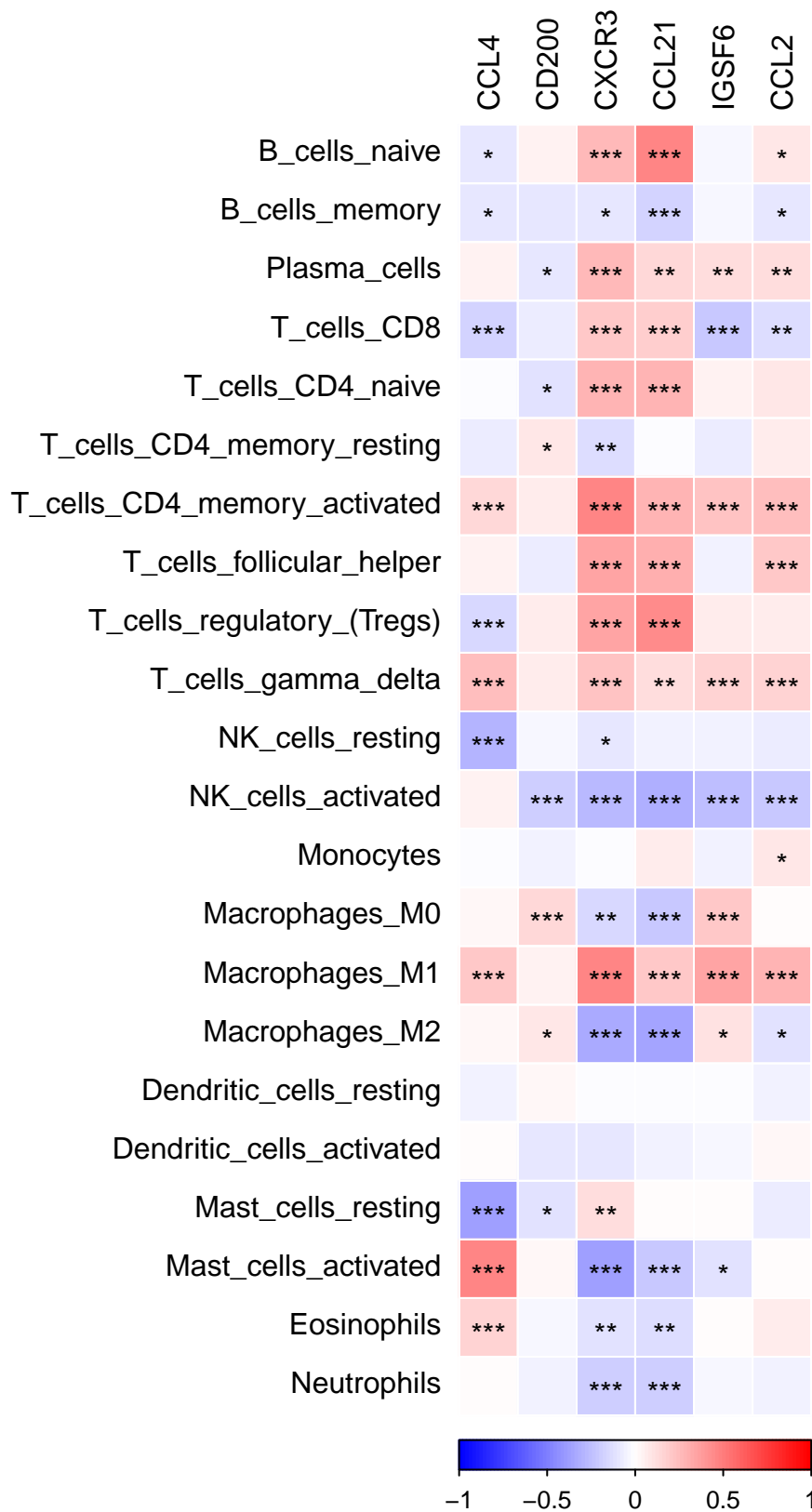

Supplement: Supplemental Information 6 [file peerj-13-19767-s006.zip › Raw Data/RNA-seq/19.TIP/results/fig9e.pdf]

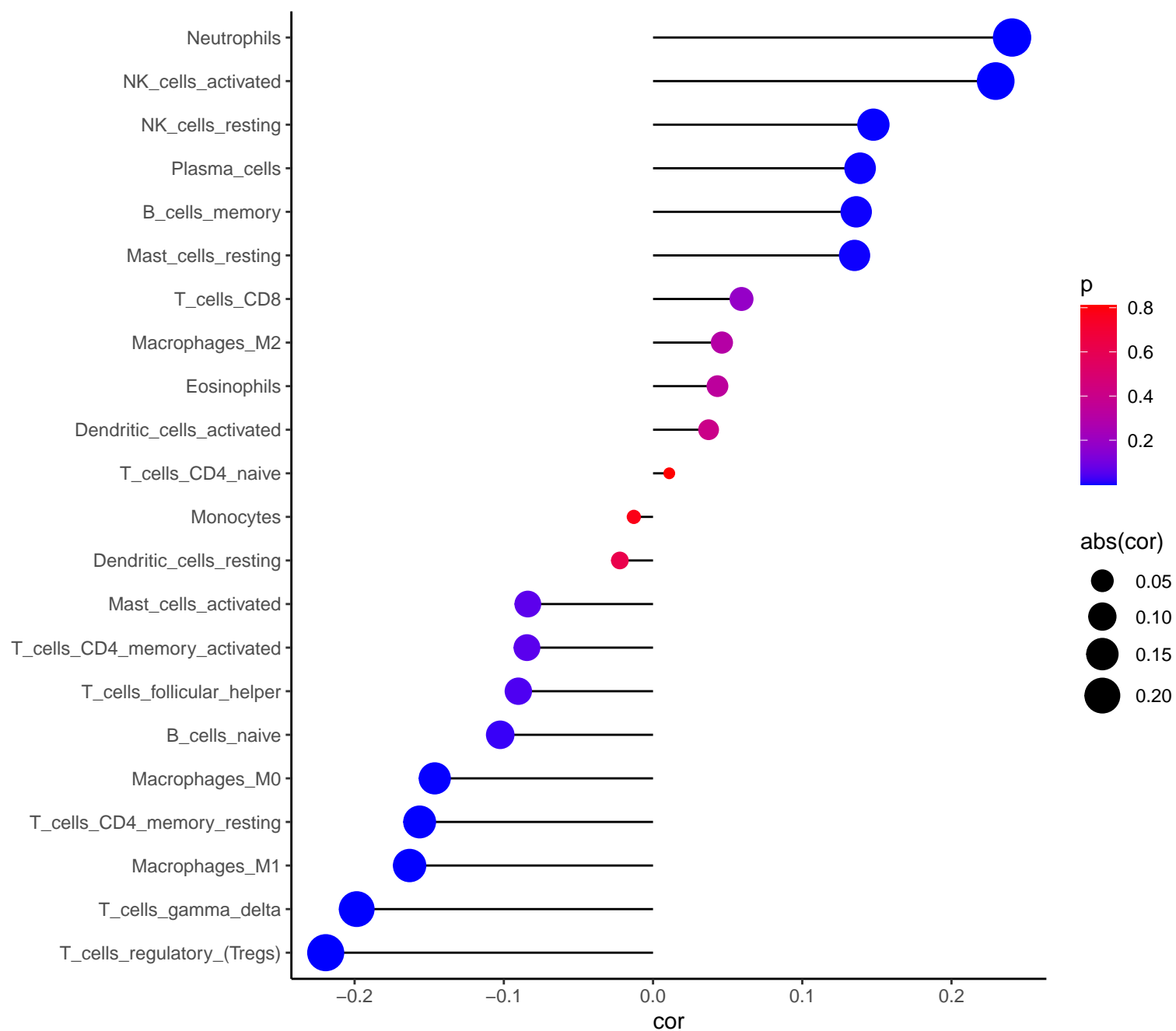

Supplement: Supplemental Information 6 [file peerj-13-19767-s006.zip › Raw Data/RNA-seq/19.TIP/results/fig9f.pdf]

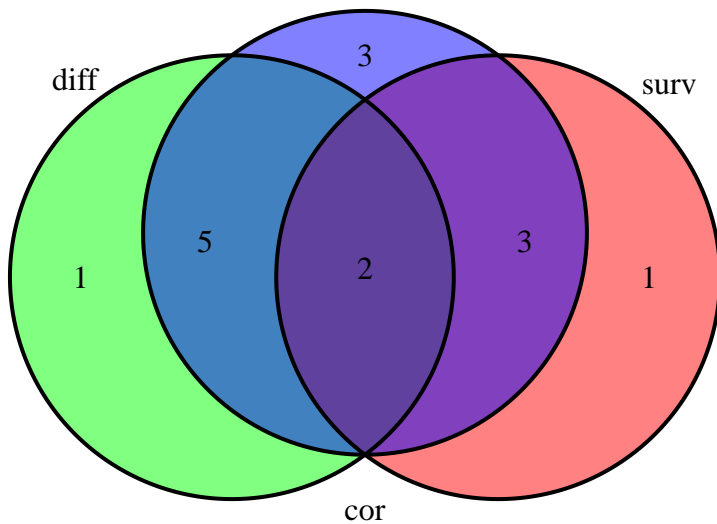

Supplement: Supplemental Information 6 [file peerj-13-19767-s006.zip › Raw Data/RNA-seq/19.TIP/results/fig9g.pdf]

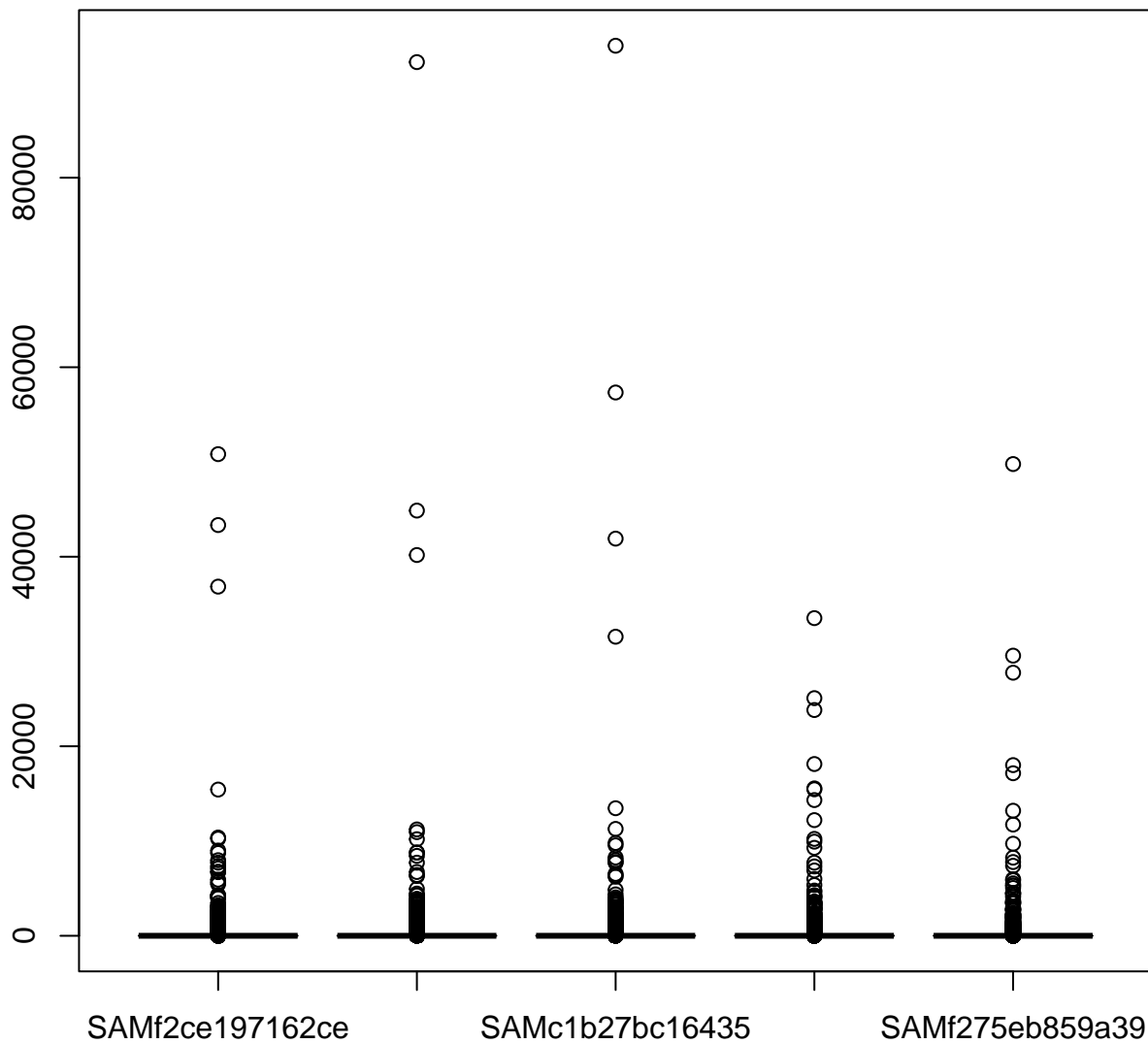

Supplement: Supplemental Information 6 [file peerj-13-19767-s006.zip › Raw Data/RNA-seq/19.TIP/results/KM/B_cells_memory.pdf]

B\_cells\_naive    High    Low

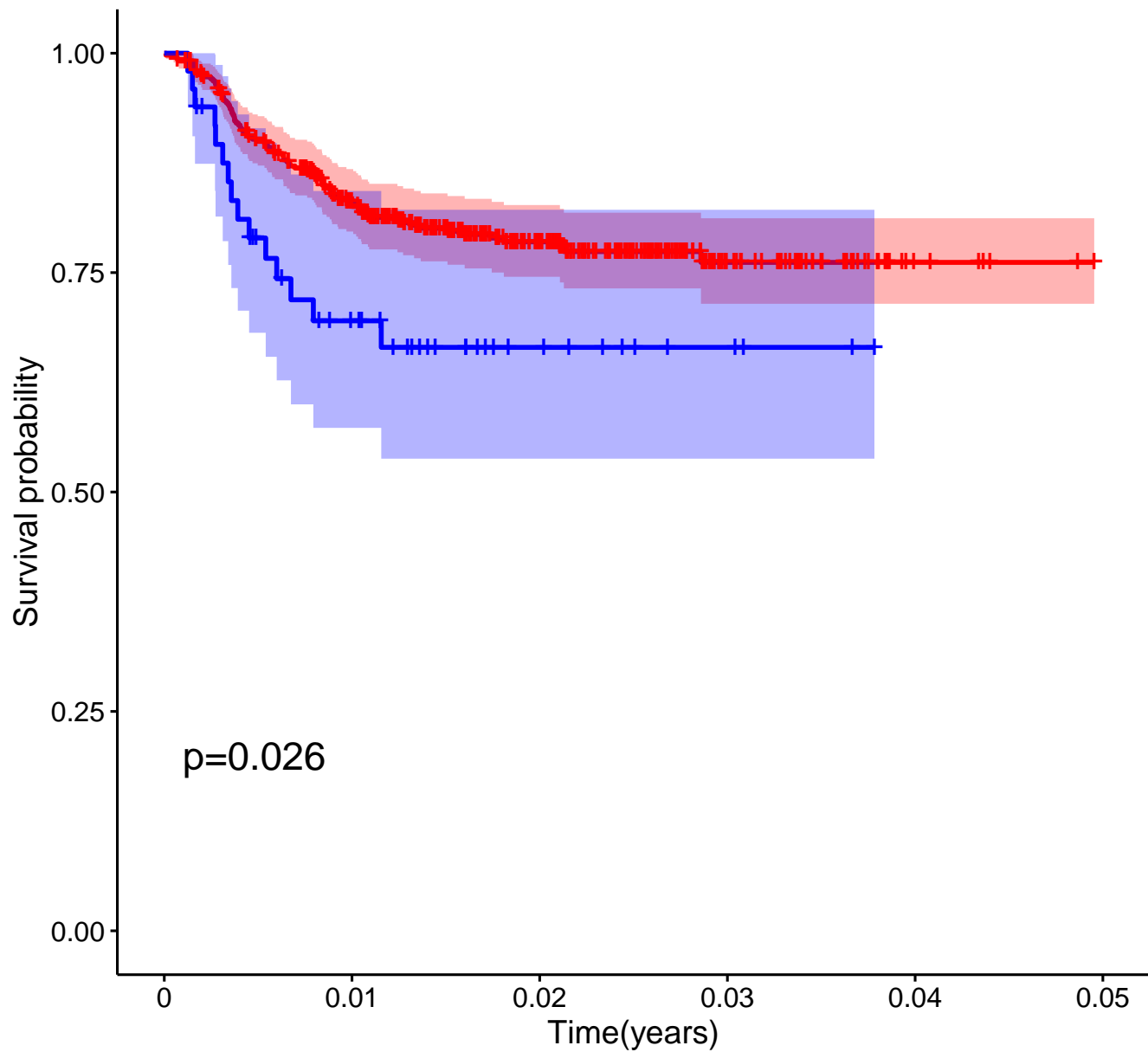

Supplement: Supplemental Information 6 [file peerj-13-19767-s006.zip › Raw Data/RNA-seq/19.TIP/results/KM/B_cells_naive.pdf]
