# Supplementary material for: Tertiary lymphoid structures-driven immune infiltration patterns and their association with survival in neuroblastoma
Source: PeerJ. 2025 Jul 22;13:e19767. doi: 10.7717/peerj.19767 (PMC12292307; doi:10.7717/peerj.19767)
Supplement: Supplemental Information 2 — A-B. Relationship between mRNA expression levels of 6 TLS genes and sensitivity to 30 common chemotherapy drugs. Blue is negatively correlated and orange is positively correlated. [file peerj-13-19767-s002.pdf]

A

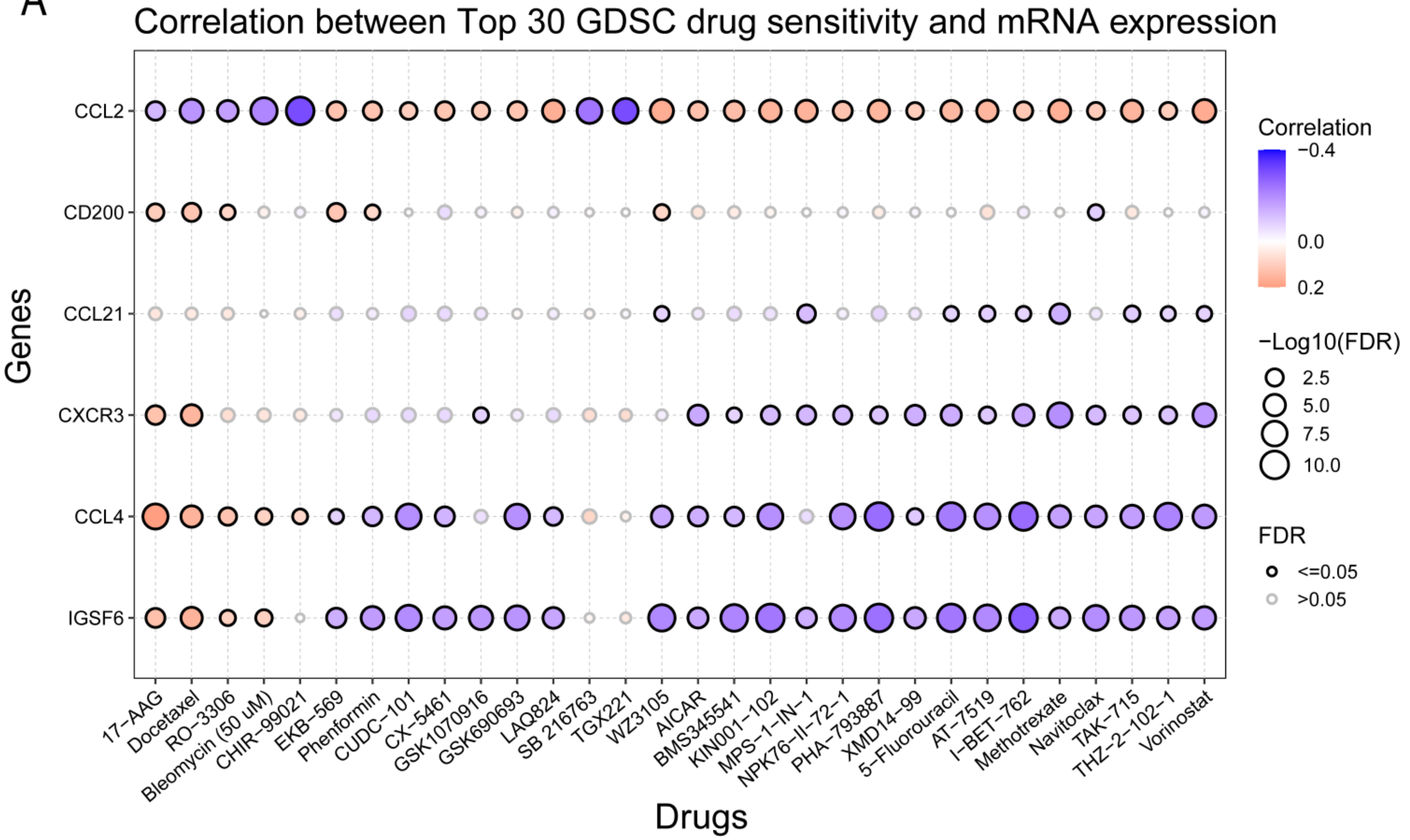
Correlation between Top 30 GDSC drug sensitivity and mRNA expression
Genes
CCL2
CD200
CCL21
CXCR3
CCL4
IGSF6
Drugs
17−AAG
Docetaxel
RO−3306
Bleomycin (50 uM)
CHIR−99021
EKB−569
Phenformin
CUDC−101
CX−5461
GSK1070916
GSK690693
LAQ824
SB 216763
TGX221
WZ3105
AICAR
BMS345541
KIN001−102
MPS−1−IN−1
NPK76−II−72−1
PHA−793887
XMD14−99
5−Fluorouracil
AT−7519
I−BET−762
Methotrexate
Navitoclax
TAK−715
THZ−2−102−1
Vorinostat
Correlation
−0.4
0.0
0.2
−Log10(FDR)
2.5
5.0
7.5
10.0
FDR
<=0.05
>0.05

B

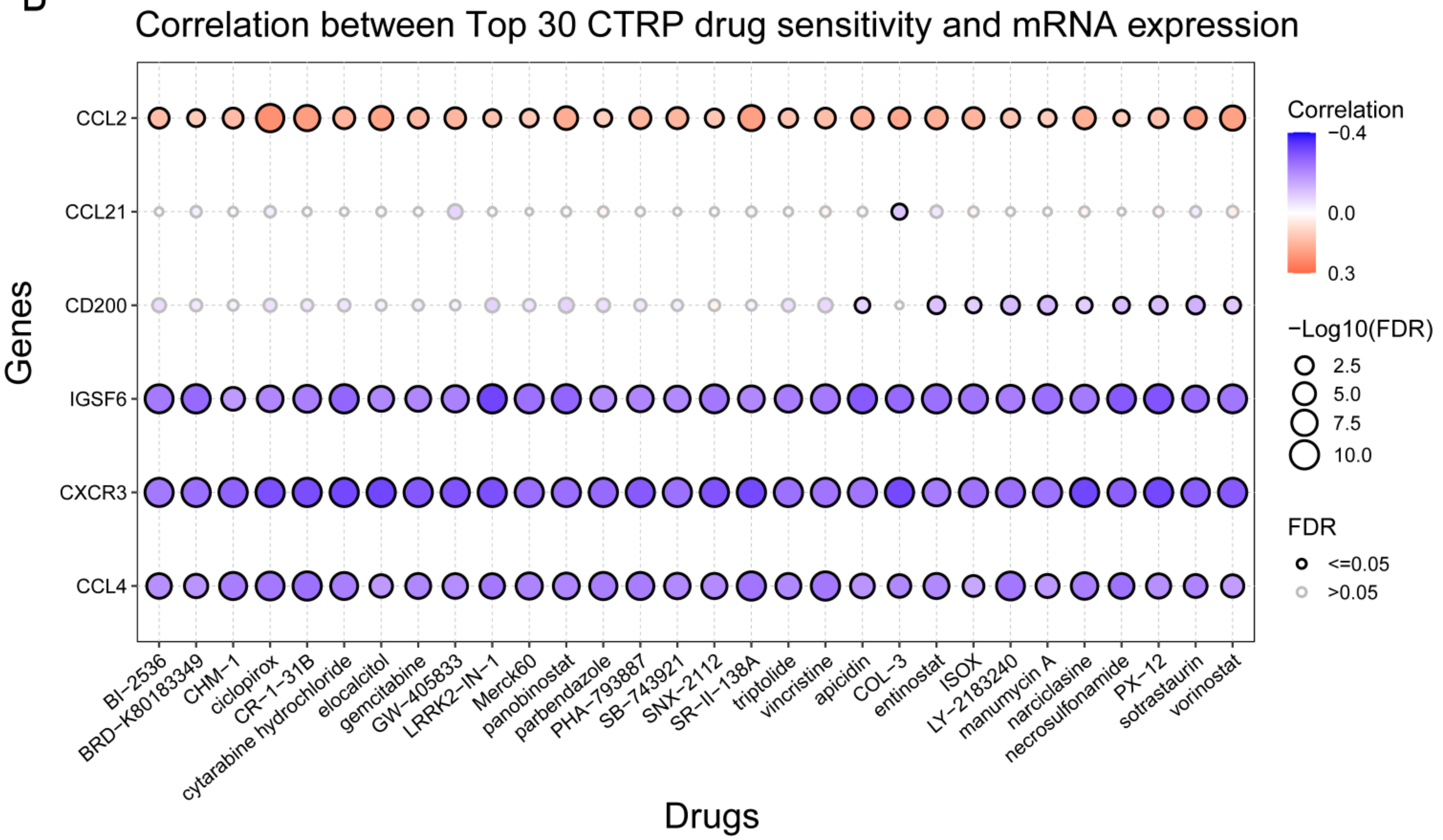
Correlation between Top 30 CTRP drug sensitivity and mRNA expression
Genes
CCL2
CCL21
CD200
IGSF6
CXCR3
CCL4
Drugs
BI−2536
BRD−K80183349
CHM−1
ciclopirox
CR−1−31B
cytarabine hydrochloride
elocalcitol
gemcitabine
GW−405833
LRRK2−IN−1
Merck60
panobinostat
parbendazole
PHA−793887
SB−743921
SNX−2112
SR−II−138A
triptolide
vincristine
apicidin
COL−3
entinostat
ISOX
LY−2183240
manumycin A
narciclasine
necrosulfonamide
PX−12
sotrastaurin
vorinostat
Correlation
−0.4
0.0
0.3
−Log10(FDR)
2.5
5.0
7.5
10.0
FDR
<=0.05
>0.05
