# Supplementary material for: Tertiary lymphoid structures-driven immune infiltration patterns and their association with survival in neuroblastoma
Source: PeerJ. 2025 Jul 22;13:e19767. doi: 10.7717/peerj.19767 (PMC12292307; doi:10.7717/peerj.19767)
Supplement: Supplemental Information 6 [file peerj-13-19767-s006.zip › Raw Data/RNA-seq/17.nom/Cox regression results.docx]

| Characteristics | Total(N) | Univariate analysis | |  | Multivariate analysis | |
| --- | --- | --- | --- | --- | --- | --- |
|  |  | Hazard ratio (95% CI) | P value |  | Hazard ratio (95% CI) | P value |
| Gender | 493 |  |  |  |  |  |
| Male | 284 | Reference |  |  |  |  |
| Female | 209 | 1.222 (0.831 - 1.797) | 0.308 |  |  |  |
| Age_day | 493 | 1.000 (1.000 - 1.000) | **< 0.001** |  | 1.000 (1.000 - 1.000) | 0.245 |
| Mycn_status | 493 |  |  |  |  |  |
| No_amplification | 401 | Reference |  |  | Reference |  |
| Amplified | 92 | 7.797 (5.265 - 11.548) | **< 0.001** |  | 1.026 (0.602 - 1.750) | 0.925 |
| Clinical_risk | 493 |  |  |  |  |  |
| High_risk | 175 | Reference |  |  | Reference |  |
| Non_high_risk | 318 | 0.048 (0.026 - 0.085) | **< 0.001** |  | 0.310 (0.119 - 0.805) | **0.016** |
| INSS_stage | 493 |  |  |  |  |  |
| Stage_4 | 181 | Reference |  |  | Reference |  |
| Stage_2 | 78 | 0.081 (0.030 - 0.220) | **< 0.001** |  | 1.232 (0.375 - 4.046) | 0.731 |
| Stage_4S | 52 | 0.122 (0.045 - 0.333) | **< 0.001** |  | 2.135 (0.574 - 7.935) | 0.258 |
| Stage_3 | 62 | 0.387 (0.219 - 0.684) | **0.001** |  | 1.357 (0.701 - 2.628) | 0.364 |
| Stage_1 | 120 | 0.013 (0.002 - 0.092) | **< 0.001** |  | 0.199 (0.025 - 1.591) | 0.128 |
| Progression | 493 |  |  |  |  |  |
| Yes | 180 | Reference |  |  | Reference |  |
| No | 313 | 0.000 (0.000 - Inf) | 0.994 |  | 0.000 (0.000 - Inf) | 0.993 |
| Riskscore | 493 | 1.090 (1.079 - 1.100) | **< 0.001** |  | 1.070 (1.057 - 1.083) | **< 0.001** |
