# Supplementary material for: Tertiary lymphoid structures-driven immune infiltration patterns and their association with survival in neuroblastoma
Source: PeerJ. 2025 Jul 22;13:e19767. doi: 10.7717/peerj.19767 (PMC12292307; doi:10.7717/peerj.19767)
Supplement: Supplemental Information 6 [file peerj-13-19767-s006.zip › Raw Data/RNA-seq/17.nom/Prognostic calibration curve/reports.html]

仙桃-预后Calibration-在线分析报告


预后Calibration-在线分析报告

导出时间: 2024-04-12 08:46:52

目录

- 预后Calibration

- 变量情况

- 中位生存时间

- 单因素Cox

- 多因素Cox

- 比例风险假设(PH)

- 方差膨胀因子(VIF)

- 方法学

预后Calibration

预后Calibration

**预后Calibration**: 描绘模型在不同时间点对应的预测概率与实际概率之间的差异情况

下载-预后校准曲线.pdf

· 横坐标为模型预测的生存概率；纵坐标为实际观测到的生存概率

· 每条线代表对应各时间点的生存情况与实际情况的对比、以及最理想的线（对角线：灰色）；越贴近对角线说明拟合情况越好

· 每条线的点代表模型预测的生存概率和实际观测到的生存概率情况（类似 nomogram中的最下部分中不同得分对应的概率）

· 每条线的点对应的竖线代表该位置的置信区间

· 每条线上蓝色的叉代表每个点经过分层 Kaplan-Meier 校正后的结果

· 顶部的竖线代表具体样本对应的生存概率（生存率的分布情况），越密集说明越多样本的生存概率在这个概率

变量情况

各个变量识别出来的类型 以及 是否纳入 进行分析

| 变量 | 类型 | 分类数量 | 缺失数量 | 是否纳入分析 | 补充说明 |
| --- | --- | --- | --- | --- | --- |
| event | 数值变量 | - | 0 | 纳入 |  |
| time | 数值变量 | - | 0 | 纳入 |  |
| Age\_day | 数值变量 | - | 0 | 纳入 |  |
| Mycn\_status | 分类变量 | 2 | 0 | 纳入 |  |
| Clinical\_risk | 分类变量 | 2 | 0 | 纳入 |  |
| INSS\_stage | 分类变量 | 5 | 0 | 纳入 |  |
| Riskscore | 数值变量 | - | 0 | 纳入 |  |

总样本数: 493

· 如果某个分类变量的分类>10，将无法识别为分类变量/等级变量

· 如果变量的分组是以 0 1 2此类进行编码，如果分类数量<5, 会被识别为分类变量；如果>5, 会被识别为数值变量

· 如果数据中含有无穷值，无穷值会被当做缺失处理

补充说明: 单因素分析前，会先去掉 结局和时间列 中的缺失的样本(时间或者结局缺失的样本是无法纳入进行分析的)

缺失处理策略: 单因素后多因素前处理变量缺失

中位生存时间

中位生存时间只针对分类变量进行，数值变量无法统计中位生存时间

Mycn\_status

| 分组 | 数目 | 总事件数 | 总删失数 | 总删失比例 | 中位生存时间 | 中位生存时间置信区间 |
| --- | --- | --- | --- | --- | --- | --- |
| No\_amplification | 401 | 53 | 348 | 86.8% | 无法计算 | ?-? |
| Amplified | 92 | 51 | 41 | 44.6% | 73.068 | 48.74-139.32 |

Clinical\_risk

| 分组 | 数目 | 总事件数 | 总删失数 | 总删失比例 | 中位生存时间 | 中位生存时间置信区间 |
| --- | --- | --- | --- | --- | --- | --- |
| High\_risk | 175 | 91 | 84 | 48.0% | 111.95 | 91.89-230.79 |
| Non\_high\_risk | 318 | 13 | 305 | 95.9% | 无法计算 | ?-? |

INSS\_stage

| 分组 | 数目 | 总事件数 | 总删失数 | 总删失比例 | 中位生存时间 | 中位生存时间置信区间 |
| --- | --- | --- | --- | --- | --- | --- |
| Stage\_4 | 181 | 81 | 100 | 55.2% | 230.79 | 113.01-? |
| Stage\_2 | 78 | 4 | 74 | 94.9% | 无法计算 | ?-? |
| Stage\_4S | 52 | 4 | 48 | 92.3% | 无法计算 | ?-? |
| Stage\_3 | 62 | 14 | 48 | 77.4% | 无法计算 | ?-? |
| Stage\_1 | 120 | 1 | 119 | 99.2% | 无法计算 | ?-? |

备注: 中位生存时间的置信区间如果有?，则代表 分组中样本较少 或者是 随访时间不足 或者是 预后相对较好无法计算出来对应的上限或者下限

单因素Cox

| 变量 | 类型 | 数量 | HR | 置信区间 | p值 |
| --- | --- | --- | --- | --- | --- |
| Age\_day | 数值变量 | 493 | 1.000 | 1.000 - 1.000 | 3.27e-15 |
| Mycn\_status | 等级变量 | 493 |  |  |  |
| No\_amplification |  | 401 | Reference |  |  |
| Amplified |  | 92 | 7.797 | 5.265 - 11.548 | 1.19e-24 |
| Clinical\_risk | 等级变量 | 493 |  |  |  |
| High\_risk |  | 175 | Reference |  |  |
| Non\_high\_risk |  | 318 | 0.048 | 0.026 - 0.085 | 2.2e-24 |
| INSS\_stage | 等级变量 | 493 |  |  |  |
| Stage\_4 |  | 181 | Reference |  |  |
| Stage\_2 |  | 78 | 0.081 | 0.030 - 0.220 | 9.13e-07 |
| Stage\_4S |  | 52 | 0.122 | 0.045 - 0.333 | 4.08e-05 |
| Stage\_3 |  | 62 | 0.387 | 0.219 - 0.684 | 0.0011 |
| Stage\_1 |  | 120 | 0.013 | 0.002 - 0.092 | 1.46e-05 |
| Riskscore | 数值变量 | 493 | 1.090 | 1.079 - 1.100 | 2.64e-72 |

表中所有变量都会纳入到多因素中

多因素Cox

| 变量 | 系数β | HR | 置信区间 | p值 |
| --- | --- | --- | --- | --- |
| Age\_day | 0.0001206 | 1.000 | 1.000 - 1.000 | 0.1214 |
| Mycn\_status |  |  |  |  |
| No\_amplification |  | Reference |  |  |
| Amplified | -0.48045 | 0.619 | 0.372 - 1.029 | 0.0642 |
| Clinical\_risk |  |  |  |  |
| High\_risk |  | Reference |  |  |
| Non\_high\_risk | -1.6398 | 0.194 | 0.077 - 0.489 | 0.0005 |
| INSS\_stage |  |  |  |  |
| Stage\_4 |  | Reference |  |  |
| Stage\_2 | 0.10315 | 1.109 | 0.328 - 3.748 | 0.8682 |
| Stage\_4S | 0.39215 | 1.480 | 0.450 - 4.868 | 0.5185 |
| Stage\_3 | 0.53554 | 1.708 | 0.888 - 3.288 | 0.1089 |
| Stage\_1 | -1.8688 | 0.154 | 0.019 - 1.242 | 0.0790 |
| Riskscore | 0.073939 | 1.077 | 1.065 - 1.088 | 1.83e-41 |

模型常数/截距(Intercept): -1.2548

原始数据一共有493个, 变量信息缺失的样本有0个, 最终纳入的样本数: 493

备注: 如果出现纳入了多因素但是对应的统计量为空的情况，说明(1)这个变量在去除变量信息缺失后某个分类数目过少(只有1个或者0个)或者是(2)存在严重共线性导致这个变量导致没办法计算。

备注: 当如果多因素中出现HR异常大或者异常小时，说明这个变量的这个分类数量过少或者是存在共线性问题导致

·(分类/等级)变量(非分组)对应的单因素p值为对应变量单因素模型全局性检验的p值，该变量是否纳入取决于此p值

△ 模型全局性统计检验情况：

·· 一致性(Concordance, C-index): 0.958(0.952-0.963)

·· Likelihood ratio test= 394.56 on 8 df, p=<2e-16

·· Wald test = 295.65 on 8 df, p=<2e-16

·· Score (logrank) test = 802.3 on 8 df, p=<2e-16

比例风险假设(PH)

Cox回归应用的前提是要求自变量满足等比例风险假设(P > 0.05)，即自变量的风险不会随着时间改变而改变，若不满足，则不适合用Cox回归进行检验。

这里只对多因素模型以及纳入的变量进行ph假设检验

备注: (1)单个变量直接PH假设和在模型里面这个变量的PH假设的结果是不一样的; (2)同一份数据不同Cox模型中同一个变量的PH假设的结果也是不一样的

| 变量 | 统计量(卡方值) | 自由度(df) | p值 |
| --- | --- | --- | --- |
| Age\_day | 13.446 | 1 | 0.0002 |
| Mycn\_status | 5.1506 | 1 | 0.0232 |
| Clinical\_risk | 4.3608 | 1 | 0.0368 |
| INSS\_stage | 13.141 | 4 | 0.0106 |
| Riskscore | 0.1274 | 1 | 0.7211 |
| GLOBAL | 29.523 | 8 | 0.0003 |

如果全局(GLOBAL)满足p > 0.05，可以认为多因素模型满足比例风险假设

方差膨胀因子(VIF)

方差膨胀因子可用于分析模型中的变量是否存在多重共线性问题

| 变量 | 类型 | VIF |
| --- | --- | --- |
| Age\_day | 数值变量 | 1.242 |
| Mycn\_status | 等级变量 |  |
| No\_amplification |  | Reference |
| Amplified |  | 1.5841 |
| Clinical\_risk | 等级变量 |  |
| High\_risk |  | Reference |
| Non\_high\_risk |  | 2.2361 |
| INSS\_stage | 等级变量 |  |
| Stage\_4 |  | Reference |
| Stage\_2 |  | 1.4549 |
| Stage\_4S |  | 1.376 |
| Stage\_3 |  | 1.3231 |
| Stage\_1 |  | 1.1142 |
| Riskscore | 数值变量 | 1.2735 |

一般认为，当0 < VIF < 10，不存在多重共线性(补充: 也有认为VIF > 4就存在多重共线性); 当10 ≤ VIF < 100，存在较强的多重共线性; 当VIF >= 100或者是出现NaN，多重共线性非常严重

方法学

**软件**: R (4.2.1)版本

**R包**: survival[3.3.1], rms[6.3-0]

**处理过程:**

· 使用survival包进行比例风险假设检验 并 进行Cox回归分析, 使用rms包进行Calibration分析与可视化

**补充说明:**

· 每次重复抽样的样本量: 40

· 抽样次数:200
