# Supplementary material for: Tertiary lymphoid structures-driven immune infiltration patterns and their association with survival in neuroblastoma
Source: PeerJ. 2025 Jul 22;13:e19767. doi: 10.7717/peerj.19767 (PMC12292307; doi:10.7717/peerj.19767)
Supplement: Supplemental Information 6 [file peerj-13-19767-s006.zip › Raw Data/RNA-seq/17.nom/Prognostic nomogram/Prognostic nomogram.pptx]

## Slide 1
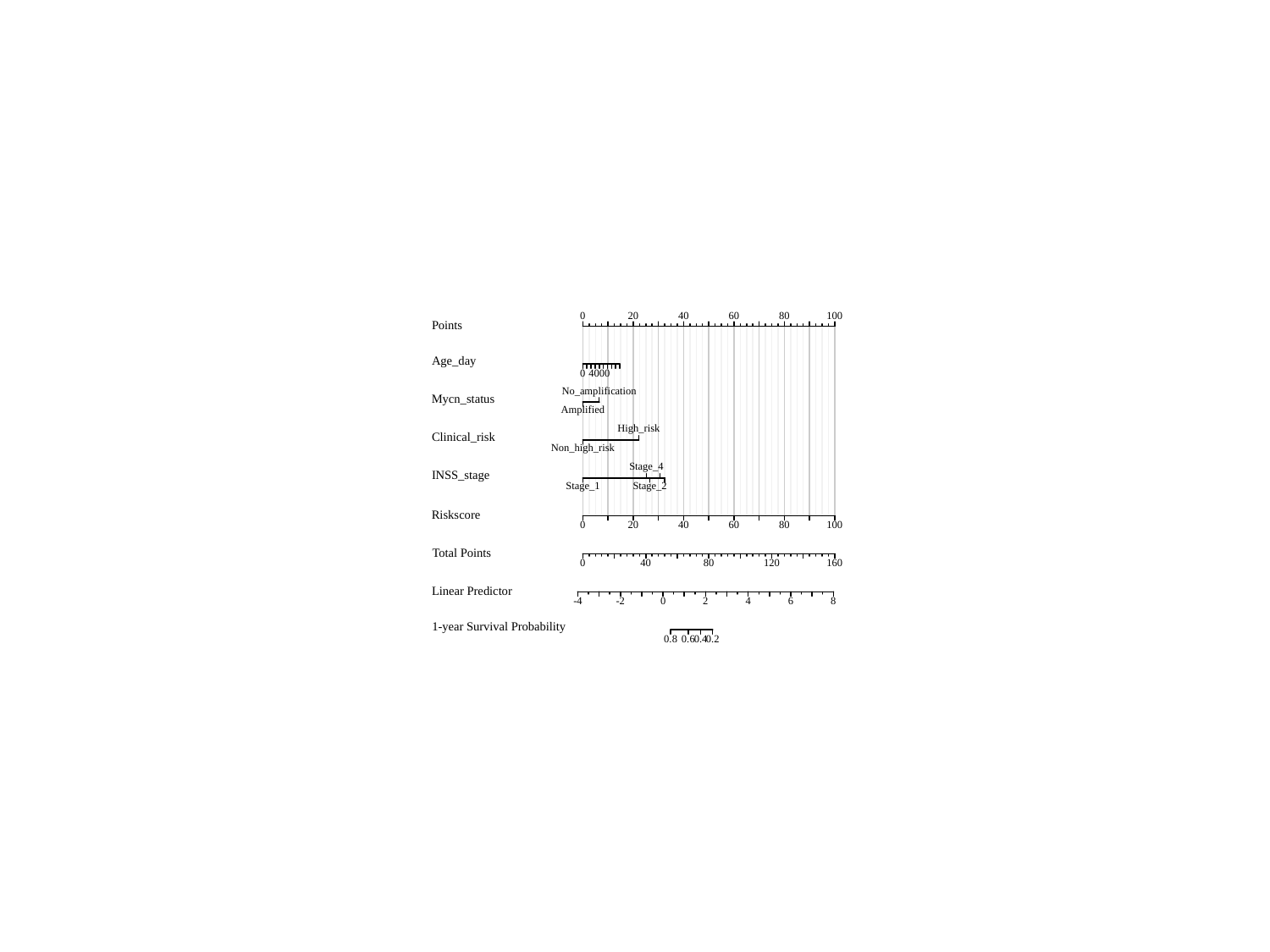

0
20
40
60
80
100
Points
Age_day
0
4000
No_amplification
Mycn_status
Amplified
High_risk
Clinical_risk
Non_high_risk
Stage_4
INSS_stage
Stage_1
Stage_2
Riskscore
0
20
40
60
80
100
Total Points
0
40
80
120
160
Linear Predictor
0
6
8
-4
-2
2
4
1-year Survival Probability
0.8
0.6
0.4
0.2
