# Supplementary material for: Tertiary lymphoid structures-driven immune infiltration patterns and their association with survival in neuroblastoma
Source: PeerJ. 2025 Jul 22;13:e19767. doi: 10.7717/peerj.19767 (PMC12292307; doi:10.7717/peerj.19767)

Characteristics	Total(N)	HR(95% CI)	P value
Gender	493		
Male	284	Reference	
Female	209	1.222 (0.831 − 1.797)	0.308
Age_day	493	1.000 (1.000 − 1.000)	< 0.001
Mycn_status	493		
No_amplification	401	Reference	
Amplified	92	7.797 (5.265 − 11.548)	< 0.001
Clinical_risk	493		
High_risk	175	Reference	
Non_high_risk	318	0.048 (0.026 − 0.085)	< 0.001
INSS_stage	493		
Stage_4	181	Reference	
Stage_2	78	0.081 (0.030 − 0.220)	< 0.001
Stage_4S	52	0.122 (0.045 − 0.333)	< 0.001
Stage_3	62	0.387 (0.219 − 0.684)	0.001
Stage_1	120	0.013 (0.002 − 0.092)	< 0.001
Progression	493		
Yes	180	Reference	
No	313	0.000 (0.000 − Inf)	0.994
Riskscore	493	1.090 (1.079 − 1.100)	< 0.001

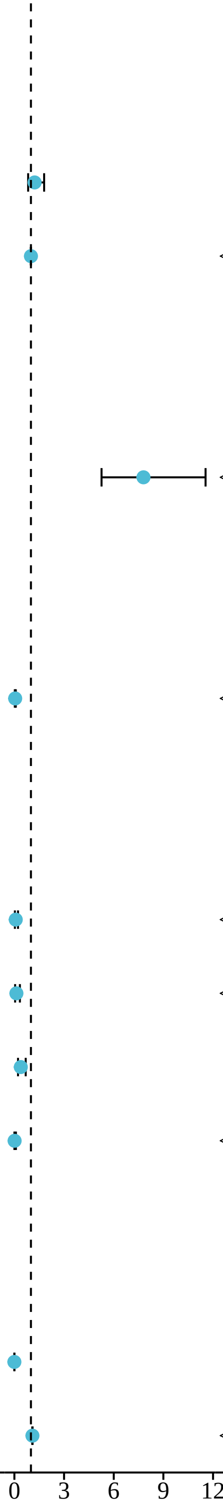

Supplement: Supplemental Information 6 [file peerj-13-19767-s006.zip › Raw Data/RNA-seq/17.nom/Single-factor forest plot/Forest map.pdf]
