# Supplementary material for: Tertiary lymphoid structures-driven immune infiltration patterns and their association with survival in neuroblastoma
Source: PeerJ. 2025 Jul 22;13:e19767. doi: 10.7717/peerj.19767 (PMC12292307; doi:10.7717/peerj.19767)
Supplement: Supplemental Information 6 [file peerj-13-19767-s006.zip › Raw Data/Table 1-gene primers.docx]

**Supplementary Table 1. gene primers and internal reference primers**

| **Gene** | **Forward primer** | **Reverse primer** |
| --- | --- | --- |
| **CCL2** | CTCGCTCAGCCAGATGCAAT | TTGGGTTTGCTTGTCCAGGT |
| **CCL4** | CCCAGCCAGCTGTGGTATTC | CCTGGACCCAGGATTCACT |
| **CCL21** | AAGGAAGATTCCCGCCAAGG | GTGACCGCTCAGTCCTCTTG |
| **CXCR3** | TGGTCCTTGAGGGGTCC | ATAGCAGTAGGCCATGACCAG |
| **CD200** | GGATGGAGAGGCTGACTCTGAC | GACCCAAACCAGGCTGTAGG |
| **IGSF6** | CTTGGACGGGTGCAAAAGTG | GCCGAGCACTCTTCTTCTTT |
| **GAPDH** | CAAGGTCATCCATGACAACTTTG | GTCCACCACCCTGTTGCTGTAG |
