# Supplementary material for: Tertiary lymphoid structures-driven immune infiltration patterns and their association with survival in neuroblastoma
Source: PeerJ. 2025 Jul 22;13:e19767. doi: 10.7717/peerj.19767 (PMC12292307; doi:10.7717/peerj.19767)
Supplement: Supplemental Information 6 [file peerj-13-19767-s006.zip › Raw Data/TLS-GENE LIST for refference.pdf]

# Tertiary lymphoid structures in the era of cancer immunotherapy

*Catherine Sautès-Fridman[1]*, Florent Petitprez[1,2], Julien Calderaro[1,3,4] and Wolf Herman Fridman[1]*

Abstract | Tertiary lymphoid structures (TLSs) are ectopic lymphoid organs that develop in non-lymphoid tissues at sites of chronic inflammation including tumours. Key common characteristics between secondary lymphoid organogenesis and TLS neogenesis have been identified. TLSs exist under different maturation states in tumours, culminating in germinal centre formation. The mechanisms that underlie the role of TLSs in the adaptive antitumour immune response are being deciphered. The description of the correlation between TLS presence and clinical benefit in patients with cancer, suggesting that TLSs could be a prognostic and predictive factor, has drawn strong interest into investigating the role of TLSs in tumours. A current major challenge is to exploit TLSs to promote lymphocyte infiltration, activation by tumour antigens and differentiation to increase the antitumour immune response. Several approaches are being developed using chemokines, cytokines, antibodies, antigen-presenting cells or synthetic scaffolds to induce TLS formation. Strategies aiming to induce TLS neogenesis in immune-low tumours and in immune-high tumours, in this case, in combination with therapeutic agents dampening the inflammatory environment and/or with immune checkpoint inhibitors, represent promising avenues for cancer treatment.

Cancer development is a multistep process involving accumulation of genetic modifications responsible for unregulated cell division, survival and death[1]. Accumulation of genetic mutations results in the expression of tumour antigens that trigger innate and adaptive antitumour immune responses to eliminate cancer cells[2]. The description of tumour-infiltrating lymphocytes (TILs) with effector and memory functions within primary tumours and their metastases and the discovery of the correlation between the density of CD8+ T effector memory cells at the site of the primary tumour and the survival of patients unambiguously demonstrated the importance of the tumour microenvironment (TME) in cancer control[3,4]. Classically, the generation of an efficient adaptive immune response against cancer occurs in secondary lymphoid organs (SLOs), wherein major histocompatibility complex (MHC) molecule–peptide complexes are presented to $CD4^+$ T and $CD8^+$ T cells by mature dendritic cells (DCs), and requires the migration of DCs from the tumour site to the SLOs[5]. B cells are activated in the SLOs upon antigen binding in primary follicles and receive help from the $CD4^+$ T cells to proliferate and form a secondary follicle that progressively becomes a germinal centre. These steps allow lymphocyte proliferation and differentiation into effector T ($T_{eff}$) cells and B memory cells that migrate into the tumour and lead to the destruction of tumour cells[5]. However, studies on the TME revealed further insight into the generation and regulation of the antitumour defences by showing that they occur not only in SLOs but also directly at the tumour site within organized cellular aggregates resembling SLOs[6] called tertiary lymphoid structures (TLSs)[7].

TLSs reflect lymphoid neogenesis occurring in peripheral tissues upon long-lasting exposure to inflammatory signals mediated by chemokines and cytokines. They develop in a variety of pathophysiological situations including autoimmune and infectious diseases, transplanted organs, inflammatory disorders and tumours, driving a range of context-dependent effects. In autoimmune syndromes, TLSs enable the local generation of autoreactive T and B cells and autoantibodies that sustain the pathogenic process[8–11]. In tissue transplants, TLS presence is associated with chronic rejection through the local production of a memory B cell alloreactive response and the production of antibodies that promote the destruction of the allograft[12,13]. During infections, studies in mouse models have shown that adaptive immune memory responses favouring antibody-mediated pathogen clearance can be generated in TLSs located within the acutely infected organs[14,15]. Persistence of infection can lead

[1]Centre de Recherche des Cordeliers, INSERM, Sorbonne Université, USPC, Université de Paris, Equipe Inflammation, complément et cancer, F-75006, Paris, France.

[2]Programme Cartes d'Identité des Tumeurs, Ligue Nationale Contre le Cancer, Paris, France.

[3]Département de Pathologie, Assistance Publique Hôpitaux de Paris, Groupe Hospitalier Henri Mondor, Créteil, France; Université Paris-Est, Créteil, France.

[4]INSERM U955, Equipe 18, Institut Mondor de Recherche Biomédicale, Créteil, France.

*e-mail: catherine.fridman@crc.jussieu.fr

https://doi.org/10.1038/s41568-019-0144-6

CD8+ T effector memory cells
A subset of CD8+ T cells with cytotoxic or interferon-γ (IFNγ)-producing functions that provide a long-lasting immunity against previously encountered antigens.

Secondary lymphoid organs
(SLOs). Organs such as the spleen, tonsils or lymph nodes that support antigen presentation to lymphoid cells to initiate and regulate the adaptive immune response.

Germinal centre
A specialized compartment within lymphoid structures that promotes B cell proliferation, differentiation, affinity maturation through somatic hypermutation and class switch recombination.

Somatic hypermutation
A process occurring during the maturation of B cells in germinal centres. Mutations occur in the variable regions of immunoglobulin heavy and light chains, enhancing affinity of the antibody for the antigen.

Class switch recombination
Otherwise known as isotype switching; a mechanism that changes the isotype of immunoglobulin produced by B cells. The constant region of the heavy chain is modified, allowing different effector functions for antibodies, such as complement activation (immunoglobulin M (IgM) and IgG) and Fcγ receptor-dependent activation (IgG), to adapt the humoral response to the antigen stimulus.

Follicular dendritic cells
(FDCs). Reticular cells found in the B cell area of lymphoid follicles. FDCs participate in the organization of the lymphoid structures and express Fcγ receptor and complement receptors. Binding of immunoglobulin G (IgG) antigen–antibody complexes on FDCs enables antigen presentation to B cells and selection of B cells expressing high-affinity antibodies.

to a progressive breach of self-tolerance resulting in autoreactive B cell activation, which can be associated with lymphoproliferative disorders such as B cell lymphoma driven by chronic hepatitis C virus infection and *Helicobacter pylori*-associated gastritis[16,17].

TLSs are also found in the stroma, invasive margin and/or core of a fraction of different tumour types[7,18]. They are composed of a T cell-rich zone with mature DCs juxtaposing a B cell follicle with germinal centre characteristics and are surrounded by plasma cells. High endothelial venules (HEVs) similar to those that allow entry of lymphocytes into SLOs can be detected at their vicinity[19]. TLSs represent privileged sites for local presentation of neighbouring tumour antigens to T cells by DCs and activation, proliferation and differentiation of T and B cells, resulting in the generation of effector memory T helper ($T_H$) cells and effector memory cytotoxic cells, memory B cells and antibody-producing plasma cells[19–23]. TLS density correlates with that of CD8+ T cells and CD4+ T cells in tumours[22]. Their presence is associated with favourable prognosis in most solid malignancies studied so far, illustrating their capacity to induce a systemic long-lasting antitumour response. In hepatocellular carcinoma (HCC), TLSs located in the adjacent parenchymal tissue correlate with an increased risk of late relapse, and it has been suggested that they could serve as niches that sustain the survival of transformed hepatic cells[4,24].

Immune checkpoint blockade to unleash antitumour immune reactions results in unprecedented rates of durable responses in a large set of cancers[25]. However, not all patients respond, and determining accurate biomarkers of response is an urgent medical need. In patients with melanoma and non-small-cell lung cancer (NSCLC) treated with anti-programmed cell death 1 (PD1), an important premise for clinical responses is the presence of pre-existing antitumour T cells and PD1 ligand 1 (PDL1) expression at the tumour site[26–28]. The CD8+ T cells are held in check by tumour and myeloid cell-expressed PDL1 and can be reinvigorated into effector cells that kill malignant cells in patients under immune checkpoint blockade. Measurement of the number of mutations carried by tumour cells (known as the tumour mutational burden) is another predictive marker associated with response[29]. However, none of these markers are fully predictive of responses to immunotherapies. Being sites for the generation of circulating effector memory cells that control tumour relapse, TLSs provide unique opportunities to guide the next generation of clinical trials in the expanding field of immuno-oncology. Notably, in two studies of NSCLC, post-treatment tumours from patients responding to neoadjuvant anti-PD1 were shown to exhibit enrichment in TLSs[30], and the presence of tumour-infiltrating PD1hiCD8+ T cells in TLSs before treatment was predictive of response to anti-PD1 (REF.[31]). Furthermore, clinical responses of patients with cervical intraepithelial neoplasia (CIN) to vaccination against human papilloma virus (HPV), the primary aetiological agent of cervical cancer, are associated with the induction of TLSs in regressing lesions[32].

In addition to being markers of therapeutic immune responses in cancers, TLSs could serve to fuel antitumour immune reactions. Indeed, one could speculate that the induction of TLSs in tumours may facilitate lymphocyte recruitment and tumour control. Thus, TLS induction could be used to open up the field of immuno-oncology treatments to immune-low tumours or immune-high tumours when used in combination with immune checkpoint inhibitors or anti-inflammatory agents. To this end, a series of approaches are being developed using chemokines, cytokines, antibodies, antigen-presenting cells or synthetic agents to promote TLS development in tumours.

In this Review, we provide a comparative analysis of TLS composition, organization and location in human cancers. We focus on the influence of TLSs on the composition of the TME and on antitumour immune responses in primary and metastatic tumours. We leverage this knowledge to discuss how TLSs could be used to predict response to therapies, track with the efficacy of treatments and be targets to manipulate antitumour immune responses. Because spontaneously occurring TLSs have been reported in only a few mouse preclinical models[33,34], most of the studies we describe here come from human cohorts, and interpretations of the data derive from correlations.

## TLS presence and composition in cancers

***Analyses in situ.*** Histopathological records of unspecific lymphocytic infiltration on haematoxylin and eosin (H&E) slides date back to 1967 (REF.[35]), when an inflammatory reaction was first reported as a prognostic feature of colorectal cancer. However, these reports were not informative of specific subpopulations of immune cells, and it took some time to acknowledge TLSs as key players in the TME. In early descriptions in melanoma[36] and NSCLC[37], TLSs were found to resemble SLOs, containing a T cell zone with clusters of CD3+ T cells and DC-lysosome-associated membrane glycoprotein (DC-LAMP; also known as LAMP3)+ mature DCs and a follicular CD20+ B cell zone (BOX 1; FIG. 1).

Recent characterization of the B cell follicles in NSCLC and ovarian cancers showed the presence of bona fide germinal centre B cells positive for the proliferation marker Ki67; some of these B cells expressed activation-induced deaminase (AID), the critical enzyme for somatic hypermutation and class switch recombination of immunoglobulin genes, as well as BCL-6, the transcription factor involved in the late stage of B cell maturation[20,38]. A network of follicular dendritic cells (FDCs) was detected in the germinal centre using CD21 or CD23 labelling. Indeed, the presence of plasma cells (CD38+CD138+) surrounding the follicle and in dense cellular aggregates is highly suggestive of production of antibodies in situ[21]. In breast cancer, CD4+ T follicular helper ($T_{FH}$) cells present in close vicinity to germinal centre B cells were found to co-express PD1 and inducible T cell co-stimulator (ICOS), with BCL-6 being the distinguishing transcription factor and interleukin-21 (IL-21) the characteristic cytokine[39]. In addition to CD4+ $T_{FH}$ cells, PD1hiCD8+ T cells that secrete CXC-chemokine ligand 13 (CXCL13) were detected close to the B cell area of TLSs in late-stage NSCLC tumours[31].

HEVs located at the periphery of TLSs[19,40] are characterized by the markers peripheral node addressin

**Box 1 | The composition and detection of tertiary lymphoid structures in cancer**

**Major cell types present in tertiary lymphoid structures (TLSs) and their markers**

- B cells are identified by surface expression of CD20 or CD19; some germinal centre B cells intracellularly express activation-induced deaminase (AID) and the proliferation marker Ki67.
- Follicular dendritic cells (FDCs) located inside the germinal centre express CD21 (also known as CR2), CD35 (also known as CR1) and CD23 (also known as FcεRII), all of which are also present on B cells.
- Plasma cells: CD138 (weak expression on late-stage germinal centre B cells) and CD269 (also present on germinal centre B cells).
- T cells: CD3, CD8 or CD4-expressing subpopulations; T helper 1 ($T_H1$) cells (intracellular T-bet$^+$), programmed cell death 1 (PD1)$^+$ T follicular helper ($T_{FH}$) cells and regulatory T ($T_{reg}$) cells (intracellular forkhead box P3 (FOXP3)$^+$).
- Dendritic cells (DCs): DC-lysosome-associated membrane glycoprotein (DC-LAMP; mature DCs), CD83 or CD86 (activated DCs).
- Neutrophils: CD66b or myeloperoxidase.
- Macrophages: CD68.
- High endothelial venules (HEVs): peripheral node addressin (PNAd) and MECA79.

**Detecting TLSs**

- Haematoxylin and eosin (H&E) staining is the simplest technique enabling the detection of TLSs in formalin-fixed paraffin-embedded tumour sections. Mature TLSs correspond to lymphoid follicles including a dense cellular aggregate resembling germinal centres found in secondary lymphoid structures (SLOs). Less differentiated structures such as lymphoid aggregates and lymphoid follicles without germinal centres can also be detected.
- Immunohistochemistry (IHC) on consecutive tumour sections or double or multiplex labelling techniques using the markers listed below followed by evaluation of TLS density, size and cellular content on scanned images using quantitative digital pathology software is commonly used. In some reports, IHC staining was found to be more accurate in the detection of TLSs than H&E staining[94].

**Markers of cell populations used for detection**

- DC-LAMP$^+$ mature DCs: a marker of TLSs in non-small-cell lung cancer (NSCLC) because they are almost exclusively found in these structures in this cancer type. However, DC-LAMP$^+$ DCs can be found outside TLSs in other cancers such as clear-cell renal cell cancer or hepatocellular carcinoma.
- CD20$^+$ B cell follicles, adjacent to a CD3$^+$ T cell zone, provide easy and convenient ways to localize TLSs.

**Location of TLSs**

- TLSs are more abundant in the invasive margin or the stroma than in the core of tumours. Therefore, whole sectioning of a tumour block with the most stromal infiltration is a reliable approach to detect TLSs. Tumour biopsy samples are disadvantageous given the small portion of tissue examined.

(PNAd) and the vascular addressin MECA79 (REF.[41]). They also express a set of mucin-like glycoproteins, ligands of L-selectin (also known as CD62L) expressed by lymphocytes, allowing their homing into tissue[42]. Using L-selectin as a marker to identify TLS T cells, the majority of T cells within TLSs were found to be of an effector memory type in lung tumours, with few central memory T cells and naive T cells; naive T cells, however, were enriched in the TLSs when compared with the rest of the tumour[22,43,44]. Cytotoxic granule-expressing CD8$^+$ T cells have been detected in TLSs, as have CD4$^+$ T cells orientated towards a $T_H1$ cell phenotype and CD4$^+$ regulatory T ($T_{reg}$) cells[21,22,45,46]. Lymphatic vessels expressing podoplanin and producing CC-chemokine ligand 21 (CCL21) that could favour emigration of educated lymphocytes to draining lymph nodes are found in the vicinity of TLSs[43,47].

Tingible-body macrophages
A type of macrophage found in germinal centres; they are able to phagocytose apoptotic lymphoid cells. Their cytoplasm contains condensed chromatin fragments.

Lymphoid tissue inducer cell
(LTi cell). A lymphoid cell that expresses and produces lymphotoxin-α and induces the formation of lymphoid tissue.

Among the innate immune cells, CD68$^+$ tingible-body macrophages have been detected in NSCLC[37], as have neutrophils in ovarian metastases[48] and prostate[49] tumours. The NCR$^+$ group 3 innate lymphoid cells (ILC3s) accumulating at the edge of TLSs in NSCLC and secreting pro-inflammatory cytokines and chemotactic factors may have lymphoid tissue inducer cell (LTi cell) functional capability[50]. In addition to FDCs, several subsets of stromal cells are also present that have an important role in TLS homeostasis[51]. Available evidence suggests that TLSs use a similar set of chemokines and adhesion molecules to SLOs for their formation: analyses of microdissected TLSs from NSCLC tumours identified the set of adhesion molecules (intercellular adhesion molecule 2 (ICAM2), ICAM3, vascular cell adhesion molecule 1 (VCAM1) and mucosal addressin cell adhesion molecule 1 (MADCAM1)) expressed by HEVs, integrins (αL, α4 and αD integrin) expressed by T cells and lymphoid chemokines (CCL19, CCL21, CXCL13, CCL17, CCL22 and IL-16) required for lymphocyte recruitment, which are similar to those found in SLOs, with the exception of IL-16 (REF.[43]). The major players and steps in TLS neogenesis are outlined and depicted in BOX 2.

***Transcriptomic analyses.*** Various gene signatures of TLSs have been proposed in the literature (TABLE 1). They are related to either chemokines or cell populations, tracing back to the neogenesis of TLSs (BOX 2). A 12-chemokine gene signature, notably containing *CCL19*, *CCL21* and *CXCL13*, was derived through correlation with a metagene related to inflammation and correlated with enhanced patient survival in colorectal cancer[52], melanoma[53] and breast cancer[54]. An eight-gene signature representing $T_{FH}$ cells, which in particular included *CXCL13*, was characterized in breast cancer[55]. A 19-gene signature related to $T_H1$ cells and B cells has also been used as a proxy for TLS presence[45]. In addition, the unique expression of CXCL13 enabled the identification of TLSs in colorectal cancer consensus molecular subtypes[56] and in soft tissue sarcoma (W.H.F., unpublished observations). In ovarian cancer, where tumour-infiltrating plasma cells were shown to be associated with TLSs, a plasma cell-specific signature comprising expression of the gene tumour necrosis factor (TNF) receptor superfamily member 17 (*TNFRSF17*; also known as *BCMA*) has been proposed[21].

Since the seminal work of Coppola et al.[52] looking at colorectal cancer, a limited number of studies have been conducted investigating what is the best TLS transcriptomic signature. Whereas TLS detection by immunohistochemistry in tissue sections is a robust and specific approach, in our experience, comparison of several signatures generated from mRNA extracted from TLS-positive cancer tissue reveals some heterogeneity of expression. Therefore, there is still a need for the development of robust TLS detection assays using data from mRNA sequencing or transcriptomic analyses of tumour biopsy samples, which would be useful for the follow-up of clinical studies.

To address the question of TLS heterogeneity among human cancers, we performed a pan-cancer analysis of

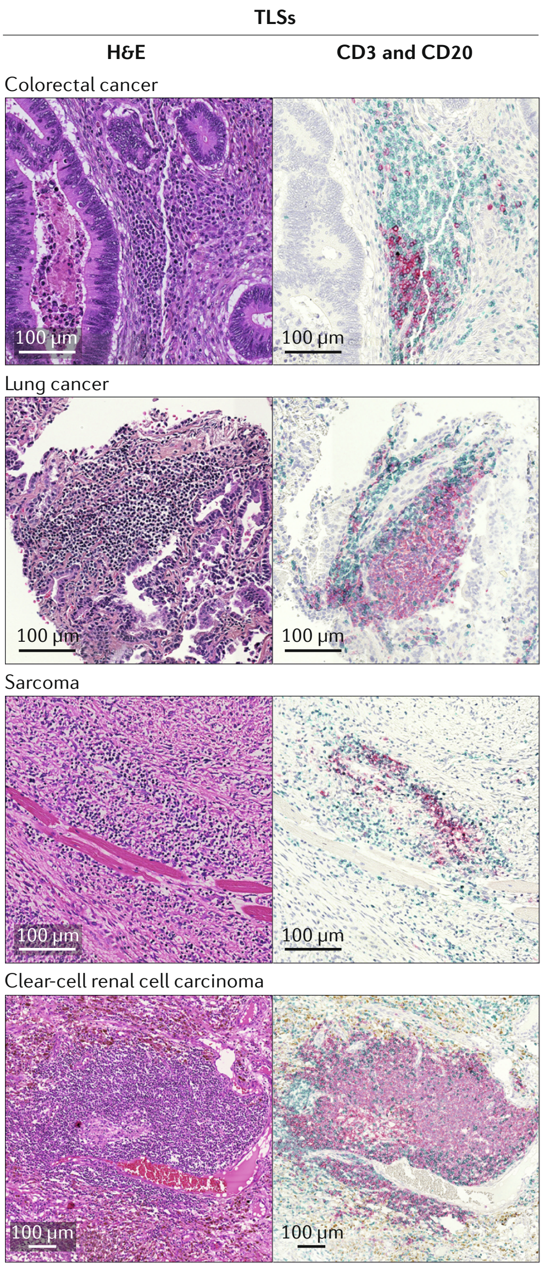


Fig. 1 | **Tertiary lymphoid structures in different cancer types.** Representative images of tertiary lymphoid structures (TLSs) detected in formalin-fixed paraffin-embedded tumour sections by haematoxylin and eosin (H&E) staining (left) or by immunohistochemistry double staining showing CD20$^{+}$ (brown) B cell zones and CD3$^{+}$ (blue) T cell zones. These histological images are original, unpublished images from the authors' examination of tumours.

the cellular composition of the TME from The Cancer Genome Atlas (TCGA) data using the deconvolution method MCP-counter[57]. The t-distributed stochastic neighbour embedding (tSNE) map (a means of visualizing high-dimensional data in a low-dimensional space of two or three dimensions) derived from this analysis enables the identification of a series of tumours strongly infiltrated by various TLS-associated populations: B lineage cells, T cells, monocytic cells (particularly myeloid DCs) and endothelial cells (FIG. 2a). Visualization of the expression of the main chemokine genes associated with TLS neogenesis on this map (*CCL19*, *CCL21*, *CXCL12* and *CXCL13*; BOX 2), as well as the 12-chemokine gene signature associated with TLS presence (TABLE 1), reveals similar profiles, with the TLS signature being strongly expressed in tumours with a higher abundance of B lineage cells and T cells, as well as myeloid DCs (FIG. 2b). Cancer type-specific expression of the 12-chemokine signature (FIG. 2c) revealed that, in most cancer types, the distribution was extremely heterogeneous, with some tumours expressing the signature at a very high level. For example, lung tumours (lung adenocarcinoma and lung squamous cell carcinoma) revealed a strong expression of the signature, hinting towards a frequent presence of TLSs. However, in some malignancies, the majority of tumours do not exhibit strong expression of the TLS signature. This is notably the case for tumours occurring in immunologically privileged sites, such as the brain (glioblastoma and lower-grade glioma) and the eye (uveal melanoma), although tumours occurring in testis (testicular germ cell tumours) exhibit a remarkably high expression of the TLS signature. In accordance with these findings, healthy brain tissues express low levels of CXCL12, CXCL13 and CCL21, and brain metastases from melanoma and breast cancer contain no or very few TLSs[58,59]. Other malignancies also presented very low expression of this signature: adrenocortical carcinoma, pheochromocytoma or paraganglioma, and most kidney carcinoma histotypes with the exception of clear-cell renal cell carcinoma. It is noteworthy that adrenocortical carcinomas derive from adrenal glands and secrete corticosteroids that were recently shown to inhibit TLS neogenesis[60]. Lastly, low expression levels of the TLS chemokine signature and a low proportion of TLSs are found in pancreatic cancers, possibly reflecting their fibrotic stroma, which might physically restrict the development of TLSs[61] (TABLE 2). Altogether, this transcriptomic analysis unveils the heterogeneity of TLS abundance both within and between human cancer types.

Adrenal glands
Endocrine glands producing hormones that participate in the regulation of the immune system, metabolism and stress. These glands secrete notably cortisol, aldosterone and androgenic steroids.

## TLS functions in cancer

***Favourable impact of TLSs on prognosis.*** Accumulating evidence indicates that TLSs play a major role in controlling tumour invasion and metastasis. A favourable impact of TLS density on overall survival and disease-free survival of patients has been observed irrespective of the detection method on sections of tumour tissue in lung cancer[20,22,37,60], colorectal cancer[23,62], pancreatic cancer[63,64], oral squamous cell carcinoma[65] and invasive breast cancer[19,40,55,66,67] (TABLE 2). This prognostic impact was independent of pathological tumour–node–metastasis (TNM) staging for lung[22], colorectal[62] and pancreatic[63] cancers. Despite a correlation between densities of TLSs and infiltrating T cells, the favourable prognostic value of both parameters was found to be independent in stage II colorectal cancer[23].

Some of the mechanisms underlying the impact of TLSs on the immune TME have been deciphered. The TLS$^{hi}$ tumours are characterized by increased proportions of CD38$^{+}$ and CD69$^{+}$ activated T cells and of

CD8+ T cells with effector memory phenotype and by overexpression of a set of genes characteristic of T cell activation, $T_H$1 cell skewing, T cell chemotaxis and T cell cytotoxicity when compared with TLS$^{low}$ tumours[19,22,45,55,63,68]. Analyses of microdissected TLSs in NSCLC revealed a strong correlation between the expression of the TLS marker DC-LAMP and this set of genes, demonstrating that TLSs orchestrate a $T_H$1 cell-polarized and cytotoxic CD8+ T cell intratumoural immune response[22]. Moreover, among patients with NSCLC who have CD8+ T cell$^{hi}$ disease, a DC-LAMP$^{low}$CD8+ T cell$^{hi}$ subgroup with a reduced likelihood of survival has been identified, suggesting that the presence of TLS DCs is needed to elicit an efficient antitumoural CD8+ T cell response[22]. Whether TLS DCs cross-present tumour antigens to CD8+ T cells directly or whether CD4+ $T_H$ cells are involved in the generation of CD8+ cytotoxic T cell responses occurring inside TLSs remains to be determined. A lower infiltration of immunosuppressive cells, as well as significantly higher expression of $T_H$17 cell-related genes, was also described in TLS$^{hi}$ versus TLS$^{low}$ pancreatic ductal adenocarcinoma (PDAC) tumours[63].

It is assumed that mature DCs present antigen to CD4+ T cells in the T cell zone of TLSs[5], but DC-LAMP+ DCs have also been detected in the germinal centres of ovarian cancer metastases, suggesting that they may be involved in antigen presentation to B cells[48]. One attractive hypothesis is that antitumour immunoglobulin G (IgG) produced locally increases the antigen presentation capacity of DCs via receptors for IgG, the Fcγ receptors (FcγRs). The ability of tumour-derived immune complexes to increase the expression of the co-stimulatory molecule CD86 on DCs in vitro and the presence of IgG deposits in TLS support this possibility[48]. The spatial organization of TLSs may favour DC priming activity for locally produced IgG.

The spatial organization of TLSs also supports the development of T cell-dependent B cell responses against protein antigens (FIGS 1,3). Expression of AID and BCL-6 necessary for the generation of somatic hypermutations and the activation of isotypic switch machineries is detected in TLS B cells, leading to the generation of effector B cells differentiating into plasma cells and of memory B cells sustaining a long-term immune response[20,38,60,62]. Analysis of the repertoire of rearranged immunoglobulin genes in the B cells of microdissected follicles, compared with peripheral B cells, revealed clonal amplification, indicating a local antigen-driven B cell response in cutaneous melanoma metastases[58], omental metastases of ovarian cancer[38,48], gastric-oesophageal adenocarcinoma[69] and invasive breast ductal carcinoma[70,71]. Chemoattractant CXCL13-producing PD1+ $T_{FH}$ cells known to help facilitate B cell responses were detected close to TLS B cells in breast cancer and in soft tissue sarcoma[39,72]. Plasma cells, notably present at the periphery of TLSs[20,39] and in dense aggregates in the tumour stroma[21], produce antitumour antibodies of the IgG isotype in vitro; IgA secretion

Box 2 | **Genesis of tertiary lymphoid structures**

The genesis of tertiary lymphoid structures (TLSs), which has mainly been elucidated using preclinical mouse models, has similarities with that of secondary lymphoid organ (SLO) formation[6,133]. Local production of the chemokine CXC-chemokine ligand 13 (CXCL13) and the cytokine interleukin-7 (IL-7) by lymphocytes or stromal cells recruits lymphoid tissue inducer (LTi) cells to the site of inflammation[134]. T helper 17 ($T_H$17) cells[135,136], B cells[137] or M1-polarized macrophages[138] have been shown to be able to substitute for LTi cells in the initiation of TLS genesis in various pathological contexts. These LTi cells interact with local stromal cells, comparable to lymphoid tissue organizer cells in SLOs, particularly through lymphotoxin-$\alpha_1\beta_2$ (LT$\alpha_1\beta_2$) binding to the LTβ receptor (LTβR)[139]. This signalling pathway leads to secretion of vascular endothelial growth factor C (VEGFC) by stromal cells[140], therefore promoting high endothelial venule (HEV) development, as well as the secretion of adhesion molecules, such as vascular cell adhesion molecule 1 (VCAM1), intercellular adhesion molecule 1 (ICAM1) or mucosal addressin cell adhesion molecule 1 (MADCAM1). The combined action of LTβ–LTβR signalling and IL-17 secretion by LTi cells leads to the production of various chemokines, notably CXCL12, CXCL13, CC-chemokine ligand 19 (CCL19) and CCL21. Together, these chemokines induce expression of LT$\alpha_1\beta_2$ on lymphocytes[141], recruit lymphocytes from nearby HEVs[142] and govern their organization into T cell and B cell zones. A proportion of the CD4+ $T_H$ cells become polarized into T follicular helper ($T_{FH}$) cells. Type I interferon and IL-1 also induce CXCL13 production[100,143]. IL-17R, IL-17 receptor.

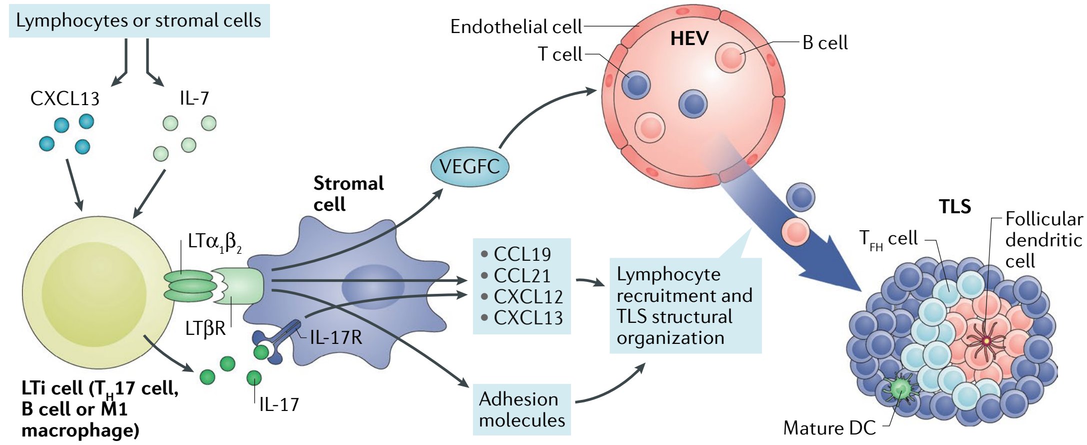

Table 1 | **Gene signatures for the detection of tertiary lymphoid structures identified from transcriptomic analyses of human cancers**

| Gene name | Protein aliases | TLS-related functions and cancer types in which the signature has been used |
|---|---|---|
| ***12-Chemokine signature[52] in colorectal cancer[52], melanoma[53], hepatocellular carcinoma[24,89] and breast cancer[54]*** | | |
| *CCL2* | MCP1 or MCAF | Monocyte, immature DC and T cell chemotaxis; cell adhesion; and cellular response to IFNγ, IL-1 and IL-6 |
| *CCL3* | MIP1α | Monocyte and T cell chemotaxis and cellular response to IFNγ, TNF and IL-1 |
| *CCL4* | MIP1β or LAG1 | Monocyte, neutrophil and T cell chemotaxis; cell adhesion; cellular response to IFNγ, TNF and IL-1; and inflammatory response |
| *CCL5* | RANTES | Monocyte, neutrophil and T cell chemotaxis; cell adhesion; cellular response to IFNγ, TNF and IL-1; and inflammatory response |
| *CCL8* | MCP2 or HC14 | Monocyte, neutrophil and T cell chemotaxis; cell adhesion; cellular response to IFNγ, TNF and IL-1; and chronic inflammatory response |
| *CCL18* | PARC, MIP4, AMAC1 or DCCK1 | Monocyte, neutrophil and T cell chemotaxis; cell adhesion; cellular response to IFNγ, TNF and IL-1; and inflammatory response |
| *CCL19* | MIP3β or ELC | Mature DC and T cell chemotaxis; T cell co-stimulation; cell maturation; cellular response to IFNγ, TNF and IL-1; inflammatory response; establishment of T cell polarity; immunological synapse formation; and positive regulation of IL-1β, IL-12 and TNF secretion |
| *CCL21* | SLC, 6Ckine or TCA4 | Mature DC and T cell chemotaxis; T cell co-stimulation; cell maturation; cellular response to IFNγ, TNF and IL-1; inflammatory response; establishment of T cell polarity; negative regulation of DC dendrite assembly; positive regulation of DC antigen presentation function; and cellular response to IL-1 and TNF |
| *CXCL9* | MIG or CMK | Neutrophil and T cell chemotaxis; $T_H$1 cell polarization; inflammatory response; and regulation of cell proliferation |
| *CXCL10* | IP10 | Neutrophil, monocyte and T cell chemotaxis; $T_H$1 cell polarization; inflammatory response; and positive regulation of cell proliferation |
| *CXCL11* | IP9 or ITAC | T cell chemotaxis; $T_H$1 cell polarization; inflammatory response; and positive regulation of cell proliferation |
| *CXCL13* | BLC, BCA1 or SCYB13 | B cell and $T_{FH}$ cell chemotaxis; germinal centre formation; lymph node development; regulation of humoral immunity; and regulation of cell proliferation |
| ***$T_{FH}$ cell signature[53] in breast cancer[55]*** | | |
| *CXCL13* | BLC, BCA1 or SCYB13 | B cell and $T_{FH}$ cell chemotaxis; germinal centre formation; lymph node development; regulation of humoral immunity; and regulation of cell proliferation |
| *CD200* | OX2 | Regulation of immune response; negative regulation of macrophage activation; and cell recognition |
| *FBLN7* | FLJ37440 or TM14 | Cell adhesion |
| *ICOS* | CD278 | T cell co-stimulation and T cell tolerance induction |
| *SGPP2* | SPP2 or SPPase2 | Regulation of immune response; positive regulation of signal transduction; and positive regulation of natural killer cell-mediated cytotoxicity |
| *SH2D1A* | NA | Signal transduction of T and B cell signal activation |
| *TIGIT* | VSTM3 or VSIG9 | T cell co-inhibitory receptor; negative regulation of IL-12 production; and positive regulation of IL-10 production |
| *PDCD1* | PD1 | T cell co-stimulation and positive regulator of T cell apoptotic process |
| ***$T_H$1 cell and B cell signature[45] in gastric cancer[45]*** | | |
| *CD4* | NA | T cell activation, differentiation and selection; and cytokine production |
| *CCR5* | CD195 | Receptor for CCL3, CCL4 and CCL5; myeloid and lymphocyte chemotaxis; inflammatory response; and positive regulation of IL-1, IL-6 and TNF production |
| *CXCR3* | CD182, CD183 or GPR9 | Receptor for CXCL9, CXCL10 and CXCL11; neutrophil and T cell chemotaxis; $T_H$1 cell polarization; inflammatory response; and cell adhesion |
| *CSF2* | GM-CSF | DC differentiation; macrophage activation; negative regulation of cytolysis; positive regulation of cell proliferation; and positive regulation of IL-23 secretion |
| *IGSF6* | DORA | Immune response |
| *IL2RA* | CD25, IL-2R or p55 | Positive regulation of activated T cell proliferation; positive regulation of T cell differentiation; inflammatory response; and regulation of T cell tolerance induction |
| *CD38* | ADPRC1 | T cell activation; positive regulation of B cell proliferation; B cell receptor signalling; cellular defence response; cell adhesion; and response to IL-1 |
| *CD40* | TNFRSF5 | B cell proliferation; inflammatory response; positive regulation of IL-12 production; isotype switching to IgG; and immunoglobulin secretion |
| *CD5* | LEU1 | T cell co-stimulation and cell proliferation |
| *MS4A1* | CD20 or LEU16 | Positive regulation of B cell lineage; B cell proliferation; and humoral immune response |

Table 1 (cont.) | **Gene signatures for the detection of tertiary lymphoid structures identified from transcriptomic analyses of human cancers**

| Gene name | Protein aliases | TLS-related functions and cancer types in which the signature has been used |
|---|---|---|
| ***$T_H$1 cell and B cell signature[45] in gastric cancer[45] (cont.)*** | | |
| *SDC1* | SDC, CD138 or syndecan | Cell migration and inflammatory response |
| *GFI1* | ZNF163 or SCN2 | Regulation of transcription |
| *IL1R1* | IL-1RA, IL-1R or CD121A | Cell surface receptor signalling; regulation of inflammatory response; and response to TGFβ |
| *IL1R2* | IL-1RB or CD121B | Inflammatory response |
| *IL10* | TGIF, GVHDS or CSIF | Positive regulation of B cell differentiation; inflammatory response; negative regulation of T and B cell proliferation; negative regulation of IFNγ, IL-1, IL-6, IL-8, IL-12, IL-18 and TNF production; negative regulation of myeloid DC activation; and regulation of isotype switching |
| *CCL20* | MIP3α, LARC or Exodus | Immature DC, monocyte, neutrophil and T cell chemotaxis; cellular response to IL-1, TNF and LPS; and inflammatory response |
| *IRF4* | MUM1 or LSIRF | T cell activation; $T_H$17 cell polarization; positive regulation of IL-2, IL-4, IL-10 and IL-13 production; and regulation of $T_H$ cell differentiation |
| *TRAF6* | RNF85 | T cell receptor signalling; $T_H$1 cell immune response; antigen processing and presentation; myeloid DC differentiation; positive regulation of T cell proliferation, activation and cytokine production; positive regulation of IL-12 production; and response to IL-1 |
| *STAT5A* | MGF | Regulation of transcription |
| ***Plasma cell signature[21] in ovarian cancer[21]*** | | |
| *TNFRSF17* | BCM, BCMA, CD269 or TNFRSF13A | Adaptive immune response; response to TNF; regulation of humoral immunity; and B cell survival |
| ***CXCL13 signature[56] in colorectal cancer[56] and soft tissue sarcoma (W.H.F., unpublished observations)*** | | |
| *CXCL13* | BLC, BCA1 or SCYB13 | B cell and $T_{FH}$ cell chemotaxis; germinal centre formation; lymph node development; regulation of humoral immunity; and regulation of cell proliferation |

CCL, CC-chemokine ligand; *CCR5*, CC-chemokine receptor 5; *CSF2*, colony-stimulating factor 2; CXCL, CXC-chemokine ligand; *CXCR3*, CXC-chemokine receptor 3; DC, dendritic cell; *FBLN7*, fibulin 7; GM-CSF, granulocyte–macrophage colony-stimulating factor; *ICOS*, inducible T cell co-stimulator; IFNγ, interferon-γ; IgG, immunoglobulin G; *IGSF6*, immunoglobulin superfamily member 6; IL, interleukin; IL-1R, interleukin-1 receptor *IRF4*, interferon regulatory factor 4; LAG1, lymphocyte activation gene 1; LPS, lipopolysaccharide; NA, not applicable; PD1, programmed cell death 1; *SDC1*, syndecan 1; *SGPP2*, sphingosine-1-phosphate phosphatase 2; *SH2D1A*, SH2 domain-containing protein 1 A; STAT5A, signal transducer and activator of transcription 5A; $T_{FH}$ cell, T follicular helper cell; TGFβ, transforming growth factor-β; $T_H$ cell, T helper cell; *TIGIT*, T cell immunoreceptor with Ig and ITIM domains; TLS, tertiary lymphoid structure; TNF, tumour necrosis factor; TNFRSF, tumour necrosis factor receptor superfamily member; *TRAF6*, tumour necrosis factor-receptor-associated factor 6.

has also been described in NSCLC, ovarian cancer and gastric-oesophageal adenocarcinoma[20,38,48,69]. Altogether, these studies demonstrate that TLSs enable the generation of effector and memory B cell responses against tumour antigens, leading to antibody production.

In addition, TLS B cells can also present antigens to T cells, including CD8$^+$ T cells. Colocalization of CD20$^+$ B cells with an abnormal memory phenotype (CD27$^-$) and expressing high levels of antigen presentation molecules with CD8$^+$ T cells was observed in ovarian cancer metastases, and the presence of both CD20$^+$ B cells and CD8$^+$ T cells correlates with increased patient survival as compared with CD8$^+$ T cells alone in this malignancy[38,73]. Cross presentation by B cells to CD8$^+$ T cells has been described, including for the cancer testis antigen NY-ESO-1 (REF.[74]), and engagement of the receptors B7 (also known as CD80) and CD40 on B cells replaces the need for CD4$^+$ T cells to transmit help to CD8$^+$ cytotoxic T cells in viral and antitumour responses[75–77]. The presence of plasma cells was also found to correlate with CD8$^+$ T cells and reinforces the positive prognostic impact of CD8$^+$ T cells[21]. Indeed, a recent meta-analysis in a large series of human cancers showed that the prognostic effect of T cells is generally stronger when tumour-infiltrating B cells or plasma cells are present, illustrating the importance of the coordination between the cellular and humoral arms of the adaptive immune system in the antitumour immune response[73].

It is striking that PD1$^{hi}$CD8$^+$ T cells, PD1$^{hi}$CD4$^+$ $T_{FH}$ cells and scarce DC-LAMP$^+$ DCs are present in the follicular B cell zone of TLSs[31,48], likely highlighting the crucial role of TLSs in facilitating T and B cell cooperation to generate and sustain effector and memory antitumour responses. Notably, the high density of TLS B cells is associated with increased CD4$^+$ T cell receptor (TCR) clonality in NSCLC[78], in accordance with the T cell-dependent B cell response occurring in TLSs. Deciphering the specificity of the immune response orchestrated by TLSs, such as through single-cell analyses coupled with TCR sequencing, would enable a deeper understanding of the intratumour immune response because only a small proportion of TILs have the capacity to recognize tumour cells[79]. Thus, in addition to CD8$^+$ TILs with antitumour specificity, bystander CD8$^+$ TILs able to recognize a wide range of epitopes unrelated to cancer, such as those from Epstein–Barr virus, human cytomegalovirus or influenza virus, have been detected in lung and colorectal cancers[80]. Importantly, an increased cytolytic activity was found in Epstein–Barr virus-infected stomach tumours and in HPV-infected

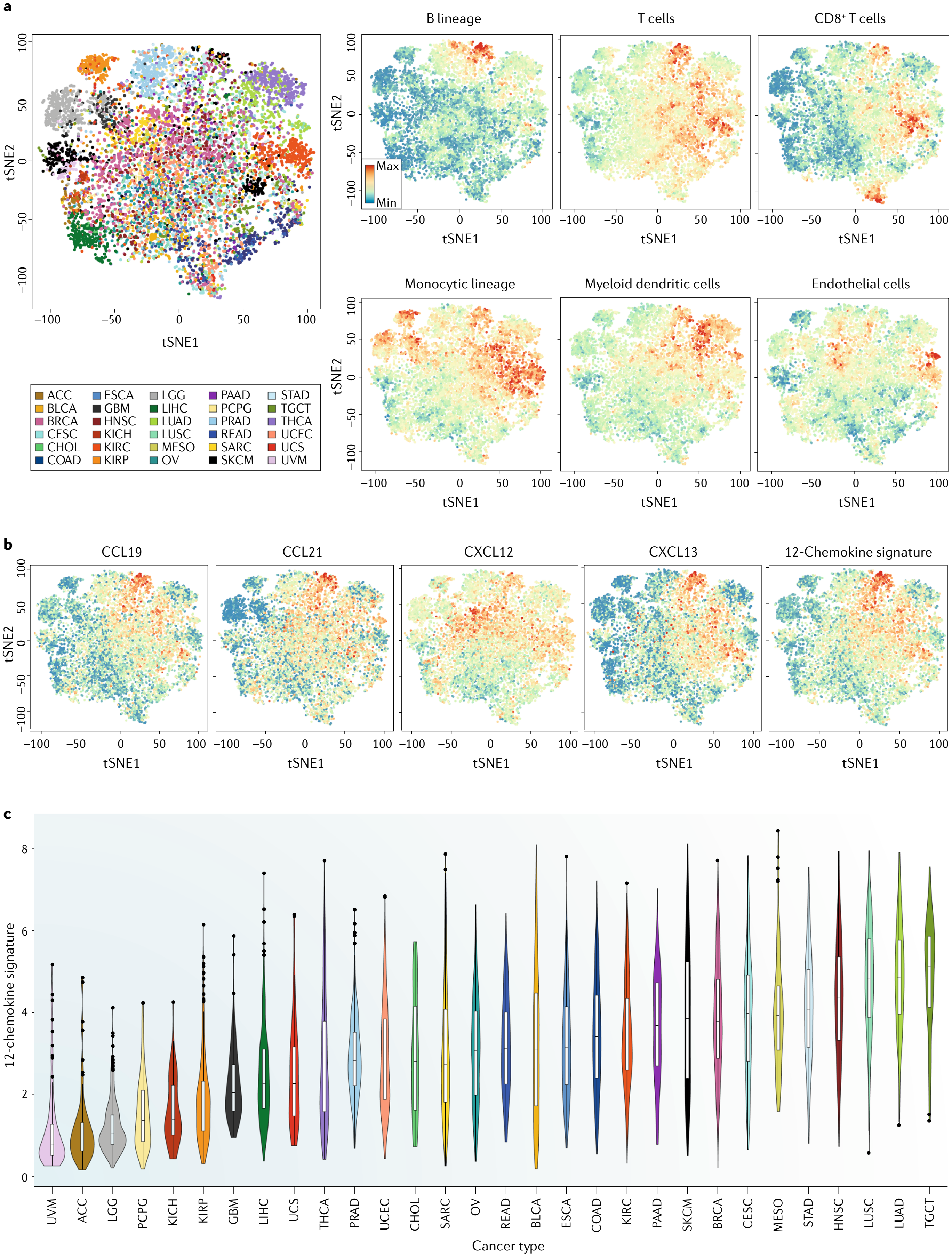
a
B lineage
T cells
CD8+ T cells
Monocytic lineage
Myeloid dendritic cells
Endothelial cells
Max
Min
tSNE1
tSNE2
ACC
BLCA
BRCA
CESC
CHOL
COAD
ESCA
GBM
HNSC
KICH
KIRC
KIRP
LGG
LIHC
LUAD
LUSC
MESO
OV
PAAD
PCPG
PRAD
READ
SARC
SKCM
STAD
TGCT
THCA
UCEC
UCS
UVM
b
CCL19
CCL21
CXCL12
CXCL13
12-Chemokine signature
c
12-chemokine signature
Cancer type
UVM
ACC
LGG
PCPG
KICH
KIRP
GBM
LIHC
UCS
THCA
PRAD
UCEC
CHOL
SARC
OV
READ
BLCA
ESCA
COAD
KIRC
PAAD
SKCM
BRCA
CESC
MESO
STAD
HNSC
LUSC
LUAD
TGCT

◀ Fig. 2 | **Pan-cancer gene expression analysis of tertiary lymphoid structure markers in 9,880 tumours.** This primary analysis was conducted by the authors for the present Review. **a** | t-distributed stochastic neighbour embedding (tSNE) map of 9,880 tumours from The Cancer Genome Atlas (TCGA) based on the MCP-counter software scores estimating the composition of the tumour microenvironment (TME). The left graph shows the cancer type of each of the samples. The six graphs on the right display the expression level of six MCP-counter cell signatures related to tertiary lymphoid structures (TLSs). To build this map, RNA-sequencing data from TCGA samples of indicated pathologies were obtained from the National Cancer Institute (NCI) Genomic Data Commons (GDC) and normalized as transcripts per million (TPM). MCP-counter[57] software was applied to all samples, and the tSNE map was constructed on the estimates with the R package tSNE. This part of the figure illustrates the strong heterogeneity of cancer types in terms of TME composition. **b** | Expression levels of CC-chemokine ligand 19 (CCL19), CCL21, CXC-chemokine ligand 12 (CXCL12), CXCL13 and the 12-chemokine signature on the tSNE map presented in part **a**. Several cancer types with a strong expression of signatures related to TLS populations can be observed. **c** | A violin plot showing the expression of the 12-chemokine TLS signature in the various cancer types as probability densities. The expression is displayed as the mean of the $\log_2$(TPM + 1) of the 12 genes of the signature. ACC, adrenocortical carcinoma; BLCA, bladder carcinoma; BRCA, breast carcinoma; CESC, cervical squamous carcinoma; CHOL, cholangiocarcinoma; COAD, colon adenocarcinoma; ESCA, oesophageal carcinoma; GBM, glioblastoma; HNSC, head and neck squamous cell carcinoma; KICH, kidney chromophobe; KIRC, kidney renal clear-cell carcinoma; KIRP, kidney renal papillary cell carcinoma; LGG, lower-grade glioma; LIHC, liver hepatocellular carcinoma; LUAD, lung adenocarcinoma; LUSC, lung squamous cell carcinoma; MESO, mesothelioma; OV, ovarian serous cystadenocarcinoma; PAAD, pancreatic adenocarcinoma; PCPG, pheochromocytoma and paraganglioma; PRAD, prostate adenocarcinoma; READ, rectum adenocarcinoma; SARC, sarcoma; SKCM, skin cutaneous melanoma; STAD, stomach adenocarcinoma; TGCT, testicular germ cell tumour; THCA, thyroid carcinoma; UCEC, uterine corpus endometrial carcinoma; UCS, uterine carcinosarcoma; UVM, uveal melanoma.

bladder and head and neck cancers[81]. Because the aetiologies of these cancers are most likely independent of this set of viruses, these bystander TILs may have been generated earlier in response to viral infections.

***TLSs and chronic inflammation: dual role of TLSs in hepatocellular carcinoma.*** Chronic inflammation driven by chemicals such as asbestos and cigarette smoke or by infectious agents such as bacteria or viruses supports tumour progression, invasion and metastasis[82]. TLS development occurs at sites of chronic inflammation and is amplified by pro-inflammatory cytokines. HCC represents an exemplar of inflammation-driven cancer characterized by cirrhotic nodules with abundant TLSs in the non-tumoural tissue[83]. In one study, expression of a 12-gene signature of TLS and detection of TLSs in the liver tissue adjacent to the tumour were found to be linked to an increased risk of late recurrence in 82 patients with HCC treated by surgical resection[24]. Late recurrence of HCC, occurring 2 years after surgery, is considered to represent de novo carcinogenesis from the inflamed liver, while early recurrence, within 2 years of surgery, results from the dissemination of residual primary tumour cells. Using a mouse model of persistent nuclear factor-κB (NF-κB) activation in hepatocytes leading to hepatitis and the development of TLSs before the formation of HCC nodules, Finkin et al.[24] showed that TLSs can serve as niches for malignant hepatocyte progenitors. These HCC progenitor cells were also detected in human HCC specimens[24,84]. This finding demonstrated a role for TLSs in the pre-malignant phase of tumour growth, contrasting with their favourable impact on the clinical outcome of patients in a series of other cancer types[4,85] (TABLE 2). An inflammatory context that may sustain TLS formation in the peri-tumoural stroma of HCC tumours has also been detected[86–88]. However, the presence of bona fide TLSs in the tumour zone of HCC, but in lower densities than those occurring in the adjacent parenchyma, was recently described by us in 2 different cohorts (490 patients in total)[89]. In contrast to the results obtained in the non-tumour liver tissue, TLSs in the tumour core were predictive of a decreased risk of early relapse by univariate and multivariate analyses[89]. The risk of recurrence was also negatively correlated with the degree of TLS maturation, with fully formed primary or secondary follicles providing a better protection than mere lymphocytic aggregates, findings that were further validated by gene expression profiling using a public data set (221 patients). By contrast, we found that TLSs in liver tissue adjacent to the tumour had no prognostic value for early and late recurrence (217 patients analysed)[89]. Altogether, these data highlight the impact of TLS location on prognosis, with TLSs located in the non-tumour liver contributing to a pro-tumoural, inflammatory state, whereas intratumoural TLSs truly reflect ongoing, effective antitumour immunity (FIG. 3).

***TLSs during tumour progression.*** The link between TLSs and histopathological parameters has been investigated and reveals discordant observations. In primary tumours from 225 patients with melanoma, the presence of HEVs correlates with good clinical characteristics, including a thin Breslow thickness, a low Clark level of invasion and tumour regression[90]. In a study of 80 patients with oral squamous cell carcinoma, TLSs were more abundant in T1 and T2 stages than in T3 and T4 stages[65]. By contrast, TLS presence correlates with higher tumour grade, stage and TIL densities and does not significantly impact on survival in other cancers (TABLE 2). For example, the abundance of TLSs increases in advanced stages (II–IV) compared with stage I gastric cancer (171 patients analysed)[33]. A pilot study (28 patients) indicated that TLSs were more common in high-grade muscle-invasive bladder cancer than in the non-muscle-invasive cancers[91]. In breast cancer, high-grade tumours were found to be twice more prone to exhibit TLSs than low-grade tumours, and the most aggressive tumour grade, grade 3, is an independent predictor for TLS formation in patients with human epidermal growth factor receptor 2 (HER2; also known as ERBB2)$^-$ tumours[66,92,93]. Nevertheless, the link between the presence of TLS and tumour grade and the decrease in prognostic value of TLSs during tumour progression do not occur in all cancers, with TLSs being found to retain their prognostic value with high prognostic impact in advanced NSCLC[22], pancreatic cancer[63] and colorectal cancer[62] in large cohorts (TABLE 2).

Several mechanisms can operate to blunt the beneficial impact of TLSs in high-grade or high-stage tumours. In a cohort of 248 patients with invasive breast cancer, 37.5% presented with TLSs, inside which tumour cells were detected in 42% of patients[92]. The presence of tumour cell-infiltrated TLSs was associated with

Breslow thickness
A measure in millimetres of the distance between the upper layer of epidermis and the deepest point of a tumour. This staging system is used as a prognostic factor for melanoma.

Clark level of invasion
A staging method used conjointly with the Breslow thickness to describe the depth of melanoma tumour invasion into the skin.

Table 2 | **Detection, prognostic and predictive impact of tertiary lymphoid structures in human cancers**

| Tumour type | Number of patients investigated | Method of TLS detection | % of patients[a] | TLS location | Prognostic value | Correlation with tumour progression | Predictive impact of TLSs | Refs |
|---|---|---|---|---|---|---|---|---|
| Bladder | 28 | IHC | 39 | Tumour | NA | Association with higher tumour grade | NA | 91 |
| Breast | 290 | IHC | 38 (TLSs more frequent in tumours not expressing oestrogen and progesterone receptors) | Tumour core and invasive margin | NA | Association with higher tumour grade | NA | 93 |
| Breast | 794 | $T_{FH}$ cell signature associated with TLSs | NA | Tumour | Favourable | NA | Correlation with pCR in 996 patients treated with chemotherapy | 55 |
| Breast | 447 | H&E | 89 (TLSs more frequent in DCIS than in invasive carcinoma) | Peri-tumoural tissue | NA | Association with TILs, higher tumour grade and MHC class I expression | TIL density associated with TLSs, which in turn correlates with longer DFS in 117 patients with $HR^{-}HER2^{+}$ tumours treated with the adjuvant targeted therapy trastuzumab | 66 |
| Breast | 248 | IHC | 42 | Tumour core and peri-tumoural tissue | Favourable in $HER2^{+}$ tumours | Association with higher tumour grade and vascular invasion | NA | 92 |
| Breast | 146 | IHC (HEV or DC-LAMP markers) | 33 | Peri-tumoural tissue | Favourable | Decreases during tumour progression | NA | 12,90 |
| Triple-negative breast | 108 | H&E | 86 | HEVs | NA | NA | TLSs correlate with pCR to neoadjuvant chemotherapy | 116 |
| Triple-negative breast | 769 | H&E | 93 | Peri-tumoural tissue | Favourable; TLSs add prognostic value to T cells | NA | NA | 59 |
| Lung metastases of clear-cell renal cell | 57 | IHC (DC-LAMP) | 53 | Invasive margin | Adverse | NA | NA | 106 |
| Colorectal | 21 | 12-Chemokine signature | NA | Tumour core and peri-tumoural tissue | Favourable | NA | No influence on response to chemotherapy or radiotherapy | 52 |
| Colorectal | 351 | H&E | 79 | Invasive margin | Favourable for patients without lymph node metastasis | NA | NA | 23 |
| Colorectal | 109 | IHC | 97 | Tumour periphery | Favourable | NA | Combined TIL–TLS score predictive of pCR in patients with stage III cancer treated with neoadjuvant chemotherapy | 54,63 |

Table 2 (cont.) | **Detection, prognostic and predictive impact of tertiary lymphoid structures in human cancers**

| Tumour type | Number of patients investigated | Method of TLS detection | % of patients[a] | TLS location | Prognostic value | Correlation with tumour progression | Predictive impact of TLSs | Refs |
|---|---|---|---|---|---|---|---|---|
| Lung metastases of colorectal | 57 | H&E | 79 | Tumour core and invasive margin | No impact | NA | No influence on response to neoadjuvant chemotherapy | 96 |
| Lung metastases of colorectal | 192 | IHC (DC-LAMP) | NA | Tumour | Favourable | NA | NA | 106 |
| Hepatocellular | 273 | H&E | 47 | Tumour | Favourable | NA | NA | 89 |
| Hepatocellular | 221 | 12-Chemokine signature | NA | Tumour | Favourable | NA | NA | 89 |
| Hepatocellular | 217 | Morphology and H&E | 50 | Non-tumoural liver tissue | No impact | NA | NA | 89 |
| Hepatocellular | 82 | 12-Chemokine signature | 15 | Non-tumoural liver tissue | Adverse | NA | NA | 24 |
| Liver metastases of colorectal | 65 | IHC (CD20) | 69 | Tumour-adjacent liver tissue interface | Favourable | NA | No influence on response to neoadjuvant chemotherapy | 107 |
| Lung | 74 | IHC (DC-LAMP) | NA | Tumour | Favourable | NA | NA | 37 |
| Lung | 74 | IHC (CD20) | NA | Tumour stroma and invasive margin | Favourable | NA | NA | 20 |
| Lung | 151 | IHC (DC-LAMP and CD8 markers) | NA | Tumour and invasive margin | Favourable | NA | After neoadjuvant chemotherapy | 120 |
| Lung | 362 | IHC (DC-LAMP) | 54 | Tumour | Favourable | NA | NA | 22 |
| Lung squamous | 138 | H&E | >95 | Tumour periphery | Favourable | NA | Prognostic impact lost in patients treated by neoadjuvant chemotherapy; TLSs decrease upon corticosteroid treatment | 60 |
| Melanoma | 82 | IHC (DC-LAMP and CD3 markers) | NA | NA | Favourable | NA | NA | 36 |
| Melanoma | 225 | HEV | NA | NA | NA | HEV density decreases in higher tumour stages | NA | 90 |
| Metastases of melanoma | 10 | 12-Chemokine signature | 42 | Peri-tumoural tissue | Favourable | NA | NA | 53 |
| Oral (squamous) | 80 | IHC | 21 | Tumour periphery | Favourable | NA | NA | 65 |
| Metastases of ovarian | 172 | IHC | 20 | Tumour stroma | Favourable association of plasma cells with TLSs | NA | NA | 21 |
| Metastases of ovarian | 147 | IHC (DC-LAMP and CD20 markers) | 50 | Tumour stroma | Favourable | NA | NA | 68 |

Table 2 (cont.) | **Detection, prognostic and predictive impact of tertiary lymphoid structures in human cancers**

| Tumour type | Number of patients investigated | Method of TLS detection | % of patients[a] | TLS location | Prognostic value | Correlation with tumour progression | Predictive impact of TLSs | Refs |
|---|---|---|---|---|---|---|---|---|
| Pancreatic | 308 | H&E | 16 | Tumour | Favourable | NA | NA | 63 |
| Pancreatic | 104 | IHC (CD20) | NA | Tumour stroma | Favourable | NA | NA | 64 |
| Prostate | 17 | IHC | NA | Tumour invasive margin | NA | Association with tumour regression | NA | 49 |
| Stomach | 365 | $T_H1$ cell and B cell signature | NA | Tumour | Favourable | NA | No influence on response to chemotherapy | 45 |
| Stomach | 176 | IHC (CD20 and CD3 markers) | NA | Tumour | NA | Association with advanced clinical disease | NA | 33 |

DCIS, ductal carcinoma in situ; DC-LAMP, DC-lysosome-associated membrane glycoprotein; DFS, disease-free survival; H&E, haematoxylin and eosin; HER2, human epidermal growth factor receptor 2; HEV, high endothelial venule; HR, hormone receptor; IHC, immunohistochemistry; MHC, major histocompatibility complex; NA, not available; pCR; pathological complete response; $T_{FH}$ cell, T follicular helper cell; $T_H1$ cell, T helper 1 cell; TIL, tumour-infiltrating lymphocyte; TLS, tertiary lymphoid structure; [a]Refers to the % of patients with TLSs or high expression of TLS markers.

lymphatic invasion, increased pathological nodal stage and nodal involvement in the HER2− (particularly oestrogen receptor-positive) tumours[92]. One could speculate that tumour cells that penetrate TLSs may metastasize more readily to regional lymph nodes. This finding is reminiscent of the detection of tumour cell progenitors in TLSs located in liver tissue surrounding HCC[24]. Other non-mutually exclusive possibilities include the fact that TLS presence cannot subvert the immunosuppressive influence of the TME in highly inflamed tumours and that TLS composition varies during tumour progression. So far, few studies have investigated the correlation between TLS abundance and T cell exhaustion in the TME as well as the presence of immunosuppressive cells within TLSs. However, an increased expression of exhaustion molecules occurring in tumours with abundant TLSs has been described in triple-negative breast cancer and HER2+ subtypes of breast cancer, which are more frequently extensively immune infiltrated and exhibit higher tumour cell proliferation and hormone receptor negativity[94]. Furthermore, the expression of PDL1 mostly found on immune cells correlates with the presence of TLSs and the extent of TIL infiltration in these aggressive tumours[94,95]. Indeed, $T_{reg}$ cell and/or myeloid-derived suppressor cell (MDSC) presence within TLS could exert a negative influence on the capacity of TLSs to generate effector and memory lymphocytes. TLS $T_{reg}$ cells were detected in breast, prostate, lung and colorectal cancers[46,49,96]. High numbers of forkhead box P3 (FOXP3)+ $T_{reg}$ cells within lymphoid aggregates surrounding primary tumours was indicative of an increased risk of disease relapse and death in 191 patients with breast cancer[46], and a decreased number of TLS $T_{reg}$ cells correlated with regression in a pilot study of 17 patients with evanescent prostate cancer[49]. In mice, the recruitment of FOXP3+ $T_{reg}$ cells and MDSCs to lymphoid aggregates in B16 melanomas engineered to express CCL21 was found to correlate with the promotion of tumour growth[97], whereas depletion of TLS $T_{reg}$ cells enhanced the co-stimulatory capacity of DCs, T cell proliferation and protective antitumour immune responses, leading to tumour regression in a spontaneous lung adenocarcinoma mouse model[34]. In addition, depletion of $T_{reg}$ cells was also associated with HEV development, higher TIL densities and reduced tumour growth rates in methylcholanthrene-induced fibrosarcomas in mice[98,99].

As alluded to above, an enrichment of TLSs has been described in patients with gastric cancer with more advanced stages (II–IV) using TCGA data sets and a three-chemokine gene signature (CXCL13, CCL19 and CCL21)[33]. Yet, correlation of the expression of this TLS signature with patient survival lacked statistical significance. In a mouse model of gastric tumours driven by a mutated form of gp130 (also known as IL-6Rβ) that induces hyperactivation of the transcription factor signal transducer and activator of transcription 3 (STAT3) in response to the IL-6 family of cytokines, it was found that the appearance of adenomas peaks with the induction of expression of the TLS gene signature and correlates with the development of TLSs in the submucosal tissue, showing the key role of inflammation in TLS formation in this model[33]. TLSs were detected in the gastric submucosa in patients with adenocarcinomas but not in patients with precursor lesions associated with gastritis[33]. The transcription factor STAT3 favours induction, maintenance and effector function of $T_H17$ cells. Studies in mouse models suggest that the role of this cell subset and of IL-17 in infection-driven and autoimmunity-driven TLS neogenesis depends on the source of antigen or the inflammatory stimulus[100]. In gastric cancer, submucosal TLS development was found to be independent of IL-17 (REF. 101). Therefore, other cytokines linked to $T_H17$ cell polarization, such as IL-1, IL-22 and IL-23, may be involved in TLS formation[100,102].

Clear-cell renal cell carcinoma belongs to the few tumour types in which high densities of CD8+ T cells correlate with poor clinical outcome[4,103]. In contrast to NSCLC, most of the DC-LAMP+ DCs (80%) were found outside the TLSs (non-TLS DCs), did not express CD83, a marker of mature DCs, and had a

**Evanescent prostate cancer**
Prostate cancer with spontaneous regression.

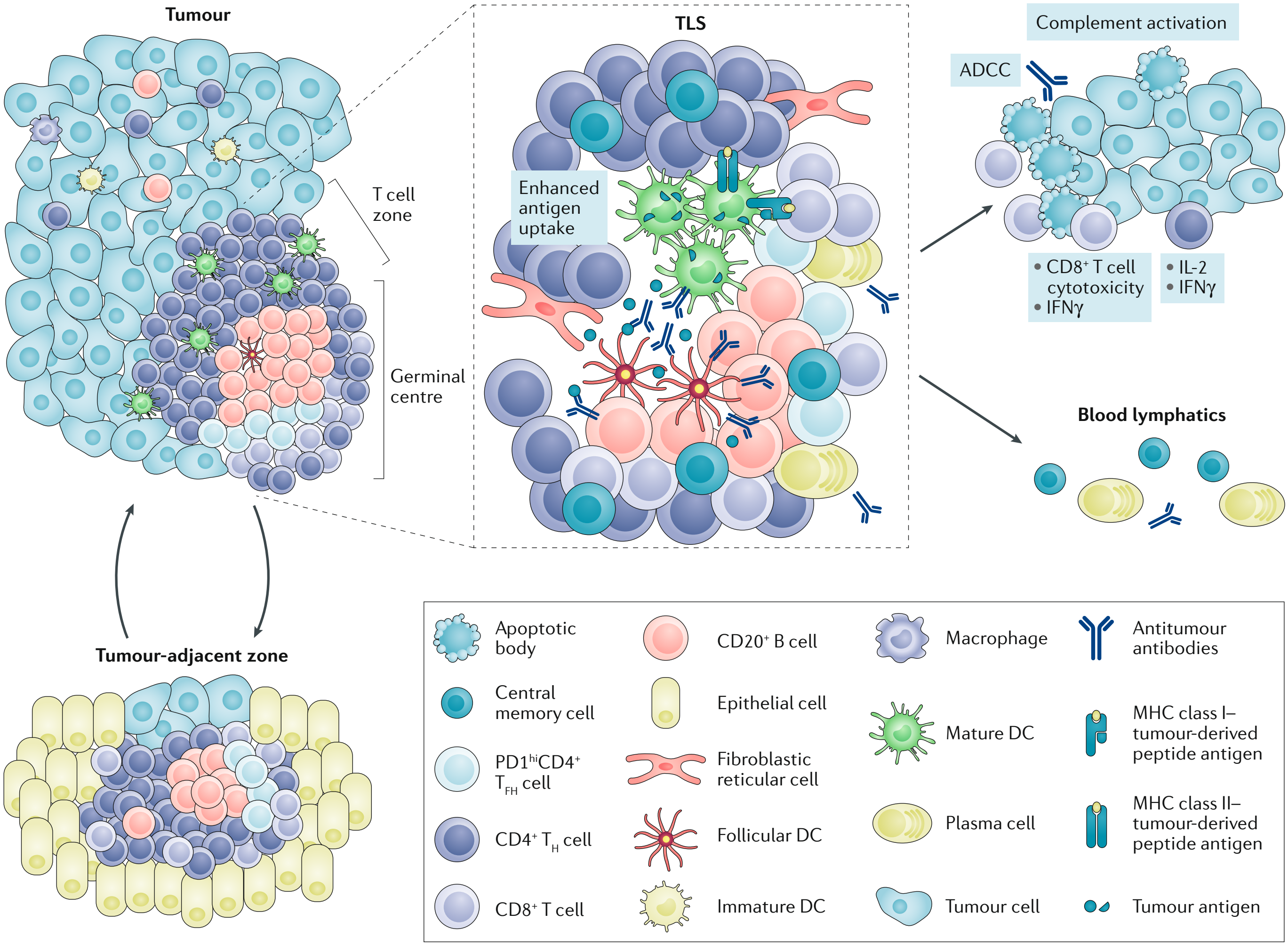


Fig. 3 | **The composition and function of tertiary lymphoid structures in cancer.** The schematic representation shows a tertiary lymphoid structure (TLS) located within a tumour with a CD3$^+$ T cell zone containing DC-lysosome-associated membrane glycoprotein (DC-LAMP)$^+$ dendritic cells (DCs) and fibroblastic reticular cells (FRCs) and a CD20$^+$ B cell zone with a germinal centre, plasma cells, antibodies forming immune complexes with tumour antigens and follicular DCs (FDCs); the coordinated actions of CD8$^+$ cytotoxic effector T cells and B cells generated in TLSs enable in situ tumour destruction via direct tumour cell killing, antibody-dependent cellular cytotoxicity (ADCC) mediated by macrophages and/or natural killer cells and local complement activation. Central memory T and B cells generated in TLSs circulate and protect against metastasis. In some cancers, TLSs located in inflamed regions adjacent to a tumour may serve as a niche for tumour cell progenitors or tumour cells that drive relapse. IFNγ, interferon-γ; IL-2, interleukin-2; MHC, major histocompatibility complex; PD1, programmed cell death 1.

low expression of MHC class II antigens, suggesting a lack of DC maturation in these tumours. Tumours with high densities of non-TLS DCs were characterized by advanced Union for International Cancer Control (UICC) TNM stages and Fuhrman grades, high densities of macrophages and PD1$^+$CD8$^+$ T cells, and higher PDL1 and PDL2 expression levels on tumour cells associated with poor patient clinical outcome[103–105]. Yet, increased densities of TLS DCs identified a small group of patients with high CD8$^+$ T cell densities and low risk of recurrence and death[103]. Although this finding needs to be confirmed in a larger set of patients, a negative correlation between the density of TLS DCs and infiltrating PD1$^+$ T cells was found, suggesting an opposite link between TLS abundance and T cell exhaustion in clear-cell renal cell carcinoma[103].

Fuhrman grades
Nuclear grades taking into account size and nuclear outline as well as nucleoli. These correlate with differentiation of tumour cells.

Antibody-dependent cellular cytotoxicity
(ADCC). A process in which target cells are identified by antibodies, which are then recognized by the Fc receptor of natural killer cells and eliminated by cytotoxic molecule release.

***TLSs in metastases.*** To date, data addressing the question of the role of TLSs in metastatic sites are scarce and discrete. However, the published analyses when gathered together present convergent information with emerging characteristics. First, TLSs are found in a proportion of metastatic sites of several cancer types, strengthening the concept that antitumour immunity is acting in advanced cancers at the metastatic level. The density of TLSs in metastases seems to depend on both the primary tumour type and the organ in which the metastatic site is located. Thus, TLS densities in lung metastases from different tumour types are highly variable and seem to mirror those of the primary sites, being at high densities in metastases from colon and prostate cancers, intermediate densities in metastases from thyroid, renal, liver and breast cancers and very low densities in metastases from

leiomyosarcoma and osteosarcoma[10,106]. In addition, the organ in which a metastasis seeds and grows also influences the density and maturation of TLSs. In melanoma, it has been reported that only skin metastases exhibit fully developed TLSs with proliferating B cells and AID expression; by contrast, FDCs were detected in some lung, muscle and gut metastases but not in brain metastases[58]. In breast cancer, analysing a large cohort of 355 metastases from 4 metastatic sites (lung, liver, brain and ovary), Lee et al.[59] reported that TLSs can be found in lung and liver metastases but not in brain and ovarian metastases.

When present at metastatic sites, TLSs have the same characteristics as in primary sites. Their density correlates with high T cell infiltration in the tumour, often with a $T_H1$ cell and cytotoxic T cell skewing[10,48,59,82,96,106,107]. The role of B cells is beginning to be addressed. In microdissected follicles from melanoma skin metastases, B lymphocytes showed a clonal amplification, rearranged immunoglobulin genes, somatic hypermutation and isotype switching, supporting the concept of a local antigen-driven B cell response[48]. Surprisingly, isotype switching occurred not only to IgG subclasses but often to IgA, despite the non-mucosal site of the metastasis. This frequent IgA production, also found in TLS-rich primary NSCLC tumours, may be due to high levels of transforming growth factor-β (TGFβ) produced in cancers[20], which acts as the switch factor for IgA[108]. In omental metastases from high-grade serous ovarian cancer, memory B lymphocytes essentially located within TLSs had a higher clonality and somatic hypermutation rate than non-tumour B cells, and they produced chemokines attracting DCs, T cells and natural killer cells; the density of B cells also correlated with that of mature DC-LAMP$^+$ DCs in the stroma of these tumours and with an immune cytolytic signature[48]. Furthermore, plasma cells were present, and IgG, IgM and IgA deposits were found on tumour cells. These antibodies, likely produced by germinal centre-generated plasma cells in TLSs, reacted with tumour-associated antigens[48], as reported in primary NSCLC[20].

Finally, as for their prognostic impact, TLSs in metastatic sites parallel that of primary tumours. Their density correlates with prolonged survival in patients with lung[106] and liver[96] metastases from colorectal cancer, lung metastases from breast cancer[59] and omental metastases from high-grade serous ovarian cancer[48], and DC-LAMP$^+$ DC density correlated with a shorter survival in lung metastases from clear-cell renal cell carcinoma[106] (TABLE 2). Altogether, data emerging from studies specifically looking at metastatic sites have illustrated the diversity of situations but support the general findings established with TLSs in primary tumours, showing that the tumour type, the organ, the degree of TLS maturation, their location with the TME and the inflammatory context of the cancerous site are essential parameters for their clinical impact.

## TLSs in cancer therapy

A large array of data supports the idea that cancer therapies that stimulate antitumour immune responses are most effective at producing long-term responses[4,109]. Although self-evident for immunotherapies[27,110], this concept has gained strength when also considering conventional chemotherapies[111], radiation therapy[112,113] and targeted therapies[114]. With TLSs being a paramount characteristic of the immune contexture of tumours[109], as they represent major sites where antitumour immune responses are generated[7,18], it has become necessary to assess their impact on response to treatment and their modulation by therapies. It is likely that the analysis of TLS densities, their location at the vicinity of or at a distance from tumour beds, their composition and degree of maturation, their impact on T and B cell receptor clonality within tumours and the antibodies produced by plasma cells educated in TLSs will become essential to predict therapeutic responses and to evaluate the efficacy of treatments. However, to date, there are only limited reports on these aspects.

***Correlation between TLSs and therapeutic response in patients.*** Several clinical trials have addressed the question of the impact of the presence of TLSs and/or of TLS-associated gene signatures on therapeutic responses to chemotherapy. They were mainly conducted within the framework of neoadjuvant clinical trials of patients with breast cancer. An eight-gene $T_{FH}$ cell signature predicted pathological complete response (pCR), as did the *CXCL13* gene alone and a $T_H1$ cell signature linked to the presence of TLSs in a cohort of 996 patients with breast cancer treated with preoperative chemotherapy[55]. In another cohort of 1,058 patients with breast cancer, the analysis of pretreatment core biopsy samples showed that high densities of both T and B lymphocytes, with the latter likely being located within TLSs, correlated with pCR induced by neoadjuvant therapy[115]. Similar findings were reported in a cohort of 108 patients with triple-negative breast cancer treated with neoadjuvant chemotherapy in which TLS density, CD8$^+$ and CD20$^+$ cell densities and *CXCL13* gene expression correlated with pCR[116]. In a cohort of 117 patients with hormone receptor-negative, HER2$^+$ breast cancer treated with adjuvant trastuzumab (anti-HER2 therapy), TIL density was associated with TLS-rich tumours and correlated with longer disease-free survival[66].

The clinical impact of TLSs on responses to therapies has very recently been extended to other cancers and to immunotherapies (W.H.F., unpublished observations). It is interesting to note that a high proportion of desmoplastic melanomas exhibit TLSs[117] and that this tumour type also has a high response rate to PD1 blockade[118].

***Therapeutic modulation of TLSs and its impact on clinical outcome.*** The effect of cancer therapies on TLSs and the resulting potential impact on patient clinical outcome have begun to be explored. In the *Kras*$^{LSL-G12D/+}$*Trp53*$^{-/-}$ (KP) genetically engineered mouse model of lung cancer, hypo-fractionated radiotherapy induces an early reduction in TLS densities followed by their restoration within 2 weeks[119]. Two independent studies of human NSCLC reported that TLSs present in lesions regressing after neoadjuvant anti-PD1 therapy[30] or chemotherapy[120] were associated with longer disease-free survival and overall survival[120]. By contrast, caution should be taken with using associated therapies, such as corticosteroids, often used to manage the side effects of chemotherapy,

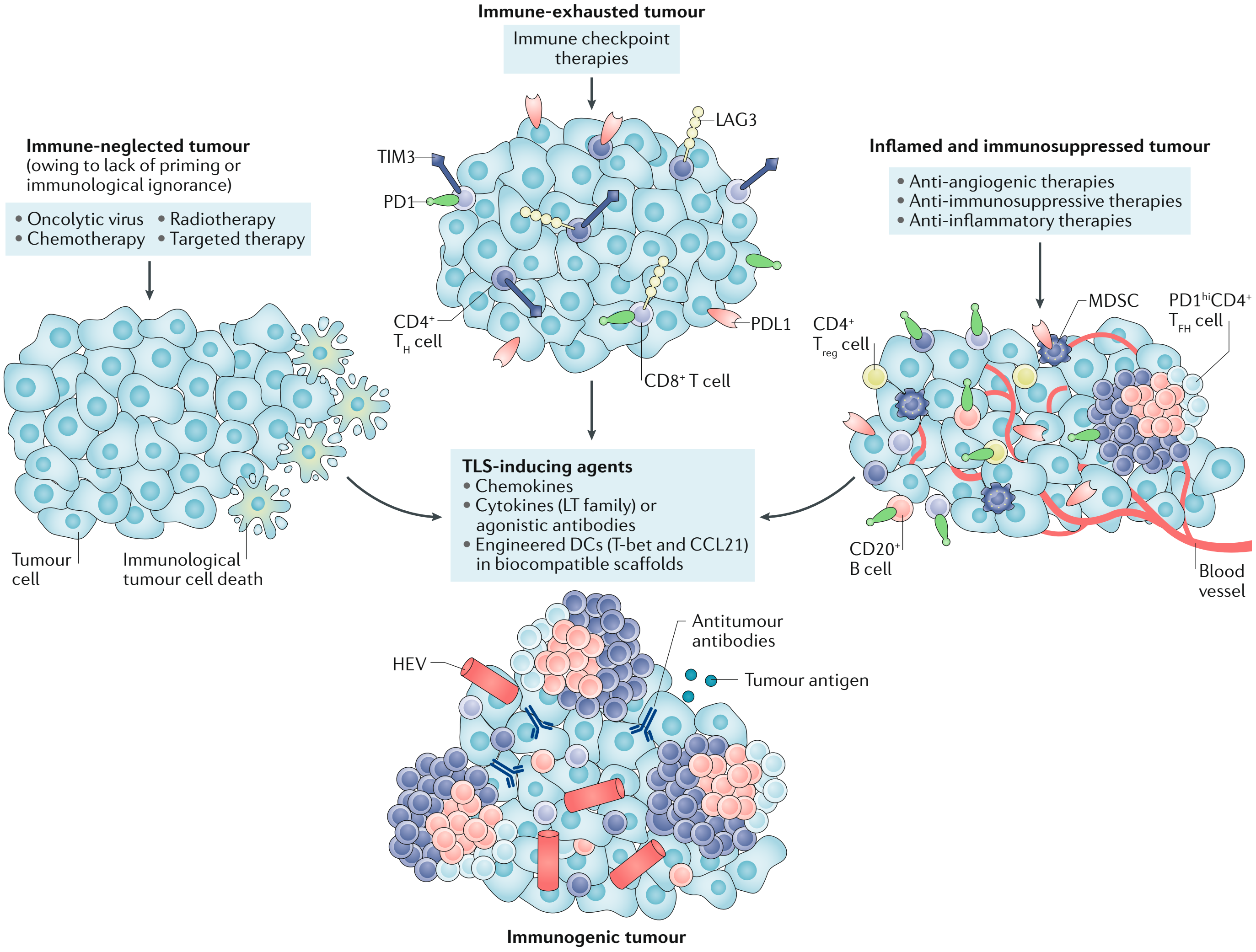


Fig. 4 | **Tertiary lymphoid structure manipulation in poor prognostic tumours.** Tertiary lymphoid structure (TLS)-inducing agents such as chemokines, cytokines, agonistic antibodies and engineered dendritic cells (DCs) expressing the T helper 1 ($T_H1$) cell transcription factor T-bet or CC-chemokine ligand 21 (CCL21) can turn tumours with an immune cold phenotype (owing to lack of priming or immunological ignorance) into immunogenic tumours (left) or enable inflamed and immunosuppressed tumours to become immunogenic when combined with anti-inflammatory and/or immunostimulatory agents (right). Immune checkpoint inhibition therapies (such as anti-programmed cell death 1 (PD1), anti-PD1 ligand 1 (PDL1), anti-T cell immunoglobulin mucin receptor 3 (TIM3) and anti-lymphocyte activation gene 3 (LAG3)) sustain TLS neogenesis to turn immune-exhausted tumours into immunogenic tumours. HEV, high endothelial venule; LT, lymphotoxin; MDSC, myeloid-derived suppressor cell; MSI, microsatellite instability; TMB, tumour mutational burden; $T_{reg}$ cell, regulatory T cell.

as they have been reported to decrease TLS density in lung squamous cell carcinoma and to impair the positive clinical impact[60]. The combination of antiangiogenic and anti-PDL1 therapies increased HEV formation and subsequent TLS formation in experimental models of breast cancer and neuroendocrine pancreatic tumours[121]. These data support the requirement for intratumoural TLSs to support the efficacy of reinvigorated antitumour responses by immune checkpoint antibodies.

The fact that TLSs may be a major component in the induction of antitumour immunity is illustrated by vaccine trials. Therapeutic vaccination of patients with high-grade CIN2–CIN3 against the E6 and E7 proteins of HPV16 and HPV18 induced TLS formation in the stroma subjacent to residual intraepithelial lesions, in which proliferating lymphocytes were found. Furthermore, clonally expanded T cells, educated in the tumour — possibly in TLSs — were identified within lesions but not in the blood. Such clonal expansions were not found in lesions of non-vaccinated patients[32]. In PDAC, which is considered to be a poorly immunogenic tumour, therapeutic vaccination with an irradiated, allogeneic granulocyte–macrophage colony-stimulating factor (GM-CSF)-secreting pancreatic tumour vaccine (GVAX) induced TLSs in 33 of 39 patients 2 weeks after treatment. Subsequent gene expression analysis of the microdissected TLSs identified pathways regulating immune cell activation and trafficking, a suppressed $T_{reg}$ cell induction pathway and an enhanced $T_H17$ cell-stimulating pathway correlating with improved survival, anti-mesothelin

Immunological ignorance
A phenomenon associated with immune cold tumour phenotypes in which tumour antigens are not immunogenic or are not presented to lymphocytes to raise an immune response.

Mesothelin
A protein that is physiologically present in mesothelial cells. This protein is immunogenic and overexpressed in several human cancers including pancreatic and lung adenocarcinoma and ovarian cancer and can be used as a tumour marker.

Fibroblastic reticular cells
(FRCs). Specialized stromal cells localized in the T cell zone of the lymph nodes and of tertiary lymphoid structures. FRCs interact with the microenvironment to regulate T cells.

T cell responses and an increased intratumoural $T_{eff}$ cell to $T_{reg}$ cell ratio[122]. These data demonstrate the potential role of TLSs in converting non-immunogenic tumours into immunogenic neoplasms (FIG. 4).

***TLS induction to increase the antitumour immune response.*** Given the data outlined in the previous subsection, it is tempting to consider the development of strategies aimed at inducing TLS neogenesis in tumours to enable the education of intratumoural T and B cells into effector and memory cells capable of recognizing the tumour. A series of recent reports using mouse models have investigated this possibility. Because TLSs develop in inflammatory sites, TNF family members are currently being used for this purpose. The lymphotoxin (LT), a member of the TNF family, system is a key regulator of lymphoid architecture (BOX 2), and the LT–LIGHT (also known as TNFSF14) signalling axis delivers critical signals for the differentiation of reticular networks into fibroblastic reticular cells (FRCs) and FDCs. In the RIP1-Tag5 mouse model of pancreatic cancer, in which tumour immunity is limited by the inability of T cells to infiltrate tumours in sufficient numbers to affect tumour growth, targeting LIGHT to tumour vessels by using a vascular targeting peptide (VTP) normalizes the vasculature, enables the influx of endogenous T cells and induces the de novo formation of intratumoural TLSs and protective immunity[123]. In addition, LIGHT–VTP enhances efficacy of anti-PD1 and anti-cytotoxic T lymphocyte antigen 4 (CTLA4) immune checkpoint blockade by inducing a large number of intratumoural effector and memory T cells with resultant survival benefit, while the addition of a tumour vaccine achieved maximal therapeutic efficacy in this model[123].

Delivery of DCs engineered to express the T cell-specific T box transcription factor T-bet into sarcoma tumour lesions (MCA205 xenografts) of mice promotes lymphocyte infiltration, $T_H1$ cell skewing and the development of TLSs and slows tumour growth[124,125]. This process is IL-36γ-dependent, as this therapeutic strategy fails when given to wild-type mice that are coadministered an IL-36 receptor antagonist or when given to IL-36-deficient mice[125]. Interestingly, IL-36γ expressed in endothelial cells also correlates with the maintenance of follicular B cells in human colorectal cancers[126]. Thus, T-bet-based and IL-36γ-based therapeutics may represent attractive tools to favour TLS formation. In addition, intratumoural delivery of *CCL21* gene-modified DCs was shown to reduce tumour burden in a transgenic mouse model of spontaneous bronchoalveolar cell carcinoma[127] and elicit systemic tumour-specific immune responses and intratumoural $CD8^+$ T cell infiltration in patients with lung cancer[128].

The HEVs surrounding the TLSs allow lymphocyte entry into tumours. Therefore, therapeutic strategies aimed at enhancing this feature would be advantageous to improve antitumour immune responses. In mouse models, depletion of $T_{reg}$ cells induces HEV formation[98] and T cell infiltration and activation, leading to tumour destruction[34,98], and it has been suggested that $T_{reg}$ cells have an important role in negatively regulating antitumour immune responses within TLSs[34,98]. In preclinical mouse models of breast and pancreatic cancers and of glioblastoma, the addition of anti-vascular endothelial growth factor receptor 2 (VEGFR2), anti-PDL1 and agonistic anti-LTβ receptor (LTβR) therapies was found to induce HEVs and enhanced cytolytic activity, resulting in tumour destruction and effectively transforming immune cold glioblastomas into immune-rich tumours[121].

Other strategies have utilized stromal cells derived from lymph nodes to induce TLSs, exploiting their architectural support, lymphogenesis and lymphocyte recruitment properties (BOX 2). The subcutaneous injection of a stromal cell line derived from lymph nodes induces TLSs that attract infiltration of host immune cell subsets and can improve the antitumour immune response against MC38 colon cancer xenografts in mice[129]. Several teams are also working on the development of synthetic scaffolds for cancer immunotherapy, aiming to induce immune responses locally, inside the tumour. In this regard, the 12-chemokine gene signature provides a good starting point for the potential construction of designer TLSs[130]. Various modified lymph node-derived cell lines are being combined with tumour antigen-pulsed DCs and then incorporated into biocompatible scaffold materials. When administered to tumour-bearing mice as injectable or implantable matrices, they can promote systemic antitumour immunity[131,132]. This nascent technology may be used in the future to induce TLSs in immune-low tumours and in immune-high tumours in combination with therapeutic agents dampening the inflammatory environment and/or with immune checkpoint inhibitors (FIG. 4).

## Conclusion and perspectives

It has become evident that TLSs are a major player in antitumour immune responses. Located in the vicinity of tumour nests, and sustained by tumour-associated inflammation, TLSs represent privileged sites for presentation of neighbouring tumour antigens by DCs and education of subsequent T and B cell responses, resulting in the generation of T effector memory cells, memory B cells and antibodies. The full repertoire of antitumour immune responses orchestrated by TLSs and SLOs remains to be deciphered, as does their interrelationship and respective dynamics during tumour progression. Usually correlating with favourable clinical outcome, TLS presence or their induction following cancer therapies predicts therapeutic responses; TLSs are also of paramount importance as a prognostic factor. Lastly, the ability to induce TLS formation through various pharmacological approaches represents a means of increasing the sensitivity of immune cold tumours to immunotherapies when used in conjunction with immune checkpoint blockade, vaccines, viruses, local intratumoural agents or intervention therapies. Conversely, in immune hot tumours with a disturbed TME with strong chronic inflammation, angiogenesis and a fibrotic stroma, the use of antiangiogenic and anti-immunosuppressive agents may help normalize the immune contexture, favouring TLS formation and therapeutic response to immune checkpoint blockade.

Published online: 15 May 2019

1. Vogelstein, B. et al. Genetic alterations during colorectal-tumor development. *N. Engl. J. Med.* **319**, 525–532 (1988).
2. Schumacher, T. N. & Schreiber, R. D. Neoantigens in cancer immunotherapy. *Science* **348**, 69–74 (2015).
3. Galon, J. et al. Type, density, and location of immune cells within human colorectal tumors predict clinical outcome. *Science* **313**, 1960–1964 (2006).
4. Fridman, W. H., Zitvogel, L., Sautès–Fridman, C. & Kroemer, G. The immune contexture in cancer prognosis and treatment. *Nat. Rev. Clin. Oncol.* **14**, 717–734 (2017).
5. Mellman, I., Coukos, G. & Dranoff, G. Cancer immunotherapy comes of age. *Nature* **480**, 480–489 (2011).
6. Drayton, D. L., Liao, S., Mounzer, R. H. & Ruddle, N. H. Lymphoid organ development: from ontogeny to neogenesis. *Nat. Immunol.* **7**, 344–353 (2006).
7. Dieu-Nosjean, M.-C. et al. Tertiary lymphoid structures, drivers of the anti-tumor responses in human cancers. *Immunol. Rev.* **271**, 260–275 (2016).
8. Lucchesi, D. & Bombardieri, M. The role of viruses in autoreactive B cell activation within tertiary lymphoid structures in autoimmune diseases. *J. Leukoc. Biol.* **94**, 1191–1199 (2013).
9. Pitzalis, C., Jones, G. W., Bombardieri, M. & Jones, S. A. Ectopic lymphoid-like structures in infection, cancer and autoimmunity. *Nat. Rev. Immunol.* **14**, 447–462 (2014).
10. Dieu-Nosjean, M.-C., Goc, J., Giraldo, N. A., Sautès-Fridman, C. & Fridman, W. H. Tertiary lymphoid structures in cancer and beyond. *Trends Immunol.* **35**, 571–580 (2014).
11. Aloisi, F. & Pujol-Borrell, R. Lymphoid neogenesis in chronic inflammatory diseases. *Nat. Rev. Immunol.* **6**, 205–217 (2006).
12. Thaunat, O. et al. Lymphoid neogenesis in chronic rejection: evidence for a local humoral alloimmune response. *Proc. Natl Acad. Sci. USA* **102**, 14723–14728 (2005).
13. Thaunat, O. et al. Chronic rejection triggers the development of an aggressive intragraft immune response through recapitulation of lymphoid organogenesis. *J. Immunol.* **185**, 717–728 (2010).
14. Moyron-Quiroz, J. E. et al. Role of inducible bronchus associated lymphoid tissue (iBALT) in respiratory immunity. *Nat. Med.* **10**, 927–934 (2004).
15. Moyron-Quiroz, J. E. et al. Persistence and responsiveness of immunologic memory in the absence of secondary lymphoid organs. *Immunity* **25**, 643–654 (2006).
16. Ramos-Casals, M., De Vita, S. & Tzioufas, A. G. Hepatitis C virus, Sjögren's syndrome and B cell lymphoma: linking infection, autoimmunity and cancer. *Autoimmun. Rev.* **4**, 8–15 (2005).
17. Mazzucchelli, L. et al. BCA-1 is highly expressed in *Helicobacter pylori*-induced mucosa-associated lymphoid tissue and gastric lymphoma. *J. Clin. Invest.* **104**, R49–R54 (1999).
18. Sautès-Fridman, C. et al. Tertiary lymphoid structures in cancers: prognostic value, regulation, and manipulation for therapeutic intervention. *Front. Immunol.* **7**, 407 (2016).
19. Martinet, L. et al. Human solid tumors contain high endothelial venules: association with T$^{-}$ and B-lymphocyte infiltration and favorable prognosis in breast cancer. *Cancer Res.* **71**, 5678–5687 (2011).
**This study shows that HEVs located around TLSs are gateways for lymphocyte entry into TLSs and have a positive prognostic impact**.
20. Germain, C. et al. Presence of B cells in tertiary lymphoid structures is associated with a protective immunity in patients with lung cancer. *Am. J. Respir. Crit. Care Med.* **189**, 832–844 (2014).
**This study describes the favourable prognostic impact of follicular B cells and the production of antitumour antibodies by intratumoural B cells**.
21. Kroeger, D. R., Milne, K. & Nelson, B. H. Tumor-infiltrating plasma cells are associated with tertiary lymphoid structures, cytolytic T-cell responses, and superior prognosis in ovarian cancer. *Clin. Cancer Res.* **22**, 3005–3015 (2016).
**This paper describes the favourable impact of tumour-infiltrating plasma cells on the antitumour immune response**.
22. Goc, J. et al. Dendritic cells in tumor-associated tertiary lymphoid structures signal a Th1 cytotoxic immune contexture and license the positive prognostic value of infiltrating CD8+T cells. *Cancer Res.* **74**, 705–715 (2014).
**This paper demonstrates the impact of mature DCs located in TLSs on the antitumour activity of CD8$^{+}$ T cells: DC-LAMP$^{+}$ DCs confer a prognostic value upon CD8$^{+}$ T cells in tumours**.
23. Di Caro, G. et al. Occurrence of tertiary lymphoid tissue is associated with T cell infiltration and predicts better prognosis in early-stage colorectal cancers. *Clin. Cancer Res.* **20**, 2147–2158 (2014).
24. Finkin, S. et al. Ectopic lymphoid structures function as microniches for tumor progenitor cells in hepatocellular carcinoma. *Nat. Immunol.* **16**, 1235–1244 (2015).
25. Ribas, A. & Wolchok, J. D. Cancer immunotherapy using checkpoint blockade. *Science* **359**, 1350–1355 (2018).
26. Taube, J. M. et al. Association of PD-1, PD-1 ligands, and other features of the tumor immune microenvironment with response to anti–PD-1 therapy. *Clin. Cancer Res.* **20**, 5064–5074 (2014).
27. Tumeh, P. C. et al. PD-1 blockade induces responses by inhibiting adaptive immune resistance. *Nature* **515**, 568–571 (2014).
28. McGranahan, N. et al. Clonal neoantigens elicit T cell immunoreactivity and sensitivity to immune checkpoint blockade. *Science* **351**, 1463–1469 (2016).
29. Samstein, R. M. et al. Tumor mutational load predicts survival after immunotherapy across multiple cancer types. *Nat. Genet.* **51**, 202–206 (2019).
30. Cottrell, T. R. et al. Pathologic features of response to neoadjuvant anti-PD-1 in resected non-small-cell lung carcinoma: a proposal for quantitative immune-related pathologic response criteria (irPRC). *Ann. Oncol.* **29**, 1853–1860 (2018).
**This paper shows that TLS presence is associated with pathological response to neoadjuvant anti-PD1**.
31. Thommen, D. S. et al. A transcriptionally and functionally distinct PD-1+ CD8 +T cell pool with predictive potential in non-small-cell lung cancer treated with PD-1 blockade. *Nat. Med.* **24**, 994–1004 (2018).
**This study demonstrates that PD1$^{hi}$CD8$^{+}$ T cells secreting CXCL13 are predominantly located in TLSs and predict response to anti-PD1 in patients**.
32. Maldonado, L. et al. Intramuscular therapeutic vaccination targeting HPV16 induces T cell responses that localize in mucosal lesions. *Sci. Transl Med.* **6**, 221ra13 (2014).
**This study shows induction of TLSs upon therapeutic vaccination**.
33. Hill, D. G. et al. Hyperactive gp130/STAT3-driven gastric tumourigenesis promotes submucosal tertiary lymphoid structure development. *Int. J. Cancer* **143**, 167–178 (2018).
34. Joshi, N. S. et al. Regulatory T cells in tumor-associated tertiary lymphoid structures suppress anti-tumor T cell responses. *Immunity* **43**, 579–590 (2015).
35. Spratt, J. S. & Spjut, H. J. Prevalence and prognosis of individual clinical and pathologic variables associated with colorectal carcinoma. *Cancer* **20**, 1976–1985 (1967).
36. Ladányi, A. et al. Density of DC-LAMP+mature dendritic cells in combination with activated T lymphocytes infiltrating primary cutaneous melanoma is a strong independent prognostic factor. *Cancer Immunol. Immunother.* **56**, 1459–1469 (2007).
37. Dieu-Nosjean, M.-C. et al. Long-term survival for patients with non-small-cell lung cancer with intratumoral lymphoid structures. *J. Clin. Oncol.* **26**, 4410–4417 (2008).
38. Nielsen, J. S. et al. CD20+tumor-infiltrating lymphocytes have an atypical CD27– memory phenotype and together with CD8+T cells promote favorable prognosis in ovarian cancer. *Clin. Cancer Res.* **18**, 3281–3292 (2012).
39. Gu-Trantien, C. et al. CXCL13-producing TFH cells link immune suppression and adaptive memory in human breast cancer. *JCI Insight* **2**, 91487 (2017).
40. Martinet, L. et al. High endothelial venule blood vessels for tumor-infiltrating lymphocytes are associated with lymphotoxin β–producing dendritic cells in human breast cancer. *J. Immunol.* **191**, 2001–2008 (2013).
41. Streeter, P. R., Rouse, B. T. & Butcher, E. C. Immunohistologic and functional characterization of a vascular addressin involved in lymphocyte homing into peripheral lymph nodes. *J. Cell Biol.* **107**, 1853–1862 (1988).
42. Girard, J.-P. & Springer, T. A. High endothelial venules (HEVs): specialized endothelium for lymphocyte migration. *Immunol. Today* **16**, 449–457 (1995).
43. de Chaisemartin, L. et al. Characterization of chemokines and adhesion molecules associated with T cell presence in tertiary lymphoid structures in human lung cancer. *Cancer Res.* **71**, 6391–6399 (2011).
44. Engelhard, V. H. et al. Immune cell infiltration and tertiary lymphoid structures as determinants of antitumor immunity. *J. Immunol.* **200**, 432–442 (2018).
45. Hennequin, A. et al. Tumor infiltration by Tbet+effector T cells and CD20+B cells is associated with survival in gastric cancer patients. *Oncoimmunology* **5**, e1054598 (2016).
46. Gobert, M. et al. Regulatory T cells recruited through CCL22/CCR4 are selectively activated in lymphoid infiltrates surrounding primary breast tumors and lead to an adverse clinical outcome. *Cancer Res.* **69**, 2000–2009 (2009).
47. Ruddle, N. H. High endothelial venules and lymphatic vessels in tertiary lymphoid organs: characteristics, functions, and regulation. *Front. Immunol.* **7**, 491 (2016).
48. Montfort, A. et al. A strong B cell response is part of the immune landscape in human high-grade serous ovarian metastases. *Clin. Cancer Res.* **23**, 250–262 (2017).
49. García-Hernández, M. L. et al. A unique cellular and molecular microenvironment is present in tertiary lymphoid organs of patients with spontaneous prostate cancer regression. *Front. Immunol.* **8**, 563 (2017).
50. Carrega, P. et al. NCR(+)ILC3 concentrate in human lung cancer and associate with intratumoral lymphoid structures. *Nat. Commun.* **6**, 8280 (2015).
51. Barone, F. et al. Stromal fibroblasts in tertiary lymphoid structures: a novel target in chronic inflammation. *Front. Immunol.* **7**, 477 (2016).
52. Coppola, D. et al. Unique ectopic lymph node-like structures present in human primary colorectal carcinoma are identified by immune gene array profiling. *Am. J. Pathol.* **179**, 37–45 (2011).
**This paper demonstrates the application of a 12-chemokine gene signature for TLS detection**.
53. Messina, J. L. et al. 12-Chemokine gene signature identifies lymph node-like structures in melanoma: potential for patient selection for immunotherapy? *Sci. Rep.* **2**, 765 (2012).
54. Prabhakaran, S. et al. Evaluation of invasive breast cancer samples using a 12-chemokine gene expression score: correlation with clinical outcomes. *Breast Cancer Res.* **19**, 71 (2017).
55. Gu-Trantien, C. et al. CD4+follicular helper T cell infiltration predicts breast cancer survival. *J. Clin. Invest.* **123**, 2873–2892 (2013).
**This study describes the favourable prognostic impact of PD1$^{hi}$CD4$^{+}$ T$_{FH}$ cells secreting CXCL13 located in TLSs**.
56. Becht, E. et al. Immune and stromal classification of colorectal cancer is associated with molecular subtypes and relevant for precision immunotherapy. *Clin. Cancer Res.* **22**, 4057–4066 (2016).
57. Becht, E. et al. Estimating the population abundance of tissue-infiltrating immune and stromal cell populations using gene expression. *Genome Biol.* **17**, 218 (2016).
58. Cipponi, A. et al. Neogenesis of lymphoid structures and antibody responses occur in human melanoma metastases. *Cancer Res.* **72**, 3997–4007 (2012).
59. Lee, M. et al. Presence of tertiary lymphoid structures determines the level of tumor-infiltrating lymphocytes in primary breast cancer and metastasis. *Mod. Pathol.* **32**, 70–80 (2018).
**This study demonstrates that the organs to which melanoma metastases seed also influence TLS densities**.
60. Silin¸a, K. et al. Germinal centers determine the prognostic relevance of tertiary lymphoid structures and are impaired by corticosteroids in lung squamous cell carcinoma. *Cancer Res.* **78**, 1308–1320 (2018).
**This paper shows that TLSs are impaired by corticosteroids**.
61. Neesse, A. et al. Stromal biology and therapy in pancreatic cancer. *Gut* **60**, 861–868 (2011).
62. Posch, F. et al. Maturation of tertiary lymphoid structures and recurrence of stage II and III colorectal cancer. *Oncoimmunology* **7**, e1378844 (2017).
63. Hiraoka, N. et al. Intratumoral tertiary lymphoid organ is a favourable prognosticator in patients with pancreatic cancer. *Br. J. Cancer* **112**, 1782–1790 (2015).
64. Castino, G. F. et al. Spatial distribution of B cells predicts prognosis in human pancreatic adenocarcinoma. *Oncoimmunology* **5**, e1085147 (2016).

65. Wirsing, A. M. et al. Presence of high-endothelial venules correlates with a favorable immune microenvironment in oral squamous cell carcinoma. *Mod. Pathol.* **31**, 910–922 (2018).
66. Lee, H. J. et al. Prognostic significance of tumor-infiltrating lymphocytes and the tertiary lymphoid structures in HER2-positive breast cancer treated with adjuvant trastuzumab. *Am. J. Clin. Pathol.* **144**, 278–288 (2015).
67. Savas, P. et al. Clinical relevance of host immunity in breast cancer: from TILs to the clinic. *Nat. Rev. Clin. Oncol.* **13**, 228–241 (2016).
68. Truxova, I. et al. Mature dendritic cells correlate with favorable immune infiltrate and improved prognosis in ovarian carcinoma patients. *J. Immunother. Cancer* **6**, 139 (2018).
69. Schlößer, H. A. et al. B cells in esophago-gastric adenocarcinoma are highly differentiated, organize in tertiary lymphoid structures and produce tumor-specific antibodies. *Oncoimmunology* **8**, e1512458 (2019).
70. Coronella, J. A. et al. Antigen-driven oligoclonal expansion of tumor-infiltrating B cells in infiltrating ductal carcinoma of the breast. *J. Immunol.* **169**, 1829–1836 (2002).
71. Nzula, S., Going, J. J. & Stott, D. I. Antigen-driven clonal proliferation, somatic hypermutation, and selection of B lymphocytes infiltrating human ductal breast carcinomas. *Cancer Res.* **63**, 3275–3280 (2003).
72. Petitprez, F. et al. A novel transcriptomic-based immune classification of soft tissue sarcoma (STS) and its association with molecular characteristics, clinical outcome and response to therapy [abstract]. *Cancer Res.* **78** (Suppl. 13), 4045 (2018).
73. Wouters, M. C. A. & Nelson, B. H. Prognostic significance of tumor-infiltrating B cells and plasma cells in human cancer. *Clin. Cancer Res.* **24**, 6125–6135 (2018).
74. Gnjatic, S. et al. Survey of naturally occurring CD4+ T cell responses against NY-ESO-1 in cancer patients: correlation with antibody responses. *Proc. Natl Acad. Sci. USA* **100**, 8862–8867 (2003).
75. Prilliman, K. R. et al. Cutting edge: a crucial role for B7-CD28 in transmitting T help from APC to CTL. *J. Immunol.* **169**, 4094–4097 (2002).
76. Schoenberger, S. P., Toes, R. E., van der Voort, E. I., Offringa, R. & Melief, C. J. T cell help for cytotoxic T lymphocytes is mediated by CD40-CD40L interactions. *Nature* **393**, 480–483 (1998).
77. Bennett, S. R. et al. Help for cytotoxic-T cell responses is mediated by CD40 signalling. *Nature* **393**, 478–480 (1998).
78. Zhu, W. et al. A high density of tertiary lymphoid structure B cells in lung tumors is associated with increased CD4(+) T cell receptor repertoire clonality. *Oncoimmunology* **4**, e1051922 (2015).
79. Scheper, W. et al. Low and variable tumor reactivity of the intratumoral TCR repertoire in human cancers. *Nat. Med.* **25**, 89–94 (2019).
80. Simoni, Y. et al. Bystander CD8+T cells are abundant and phenotypically distinct in human tumour infiltrates. *Nature* **557**, 575–579 (2018).
81. Rooney, M. S., Shukla, S. A., Wu, C. J., Getz, G. & Hacohen, N. Molecular and genetic properties of tumors associated with local immune cytolytic activity. *Cell* **160**, 48–61 (2015).
82. Becht, E. et al. Immune contexture, immunoscore, and malignant cell molecular subgroups for prognostic and theranostic classifications of cancers. *Adv. Immunol.* **130**, 95–190 (2016).
83. Calderaro, J. et al. Histological subtypes of hepatocellular carcinoma are related to gene mutations and molecular tumour classification. *J. Hepatol.* **67**, 727–738 (2017).
84. Pikarsky, E. & Heikenwalder, M. Focal and local: ectopic lymphoid structures and aggregates of myeloid and other immune cells in liver. *Gastroenterology* **151**, 780–783 (2016).
85. Sautès-Fridman, C. & Fridman, W. H. TLS in tumors: what lies within. *Trends Immunol.* **37**, 1–2 (2016).
86. Kuang, D.-M. et al. Activated monocytes in peritumoral stroma of hepatocellular carcinoma promote expansion of memory T helper 17 cells. *Hepatology* **51**, 154–164 (2010).
87. Chen, M.-M. et al. Polarization of tissue-resident TFH-like cells in human hepatoma bridges innate monocyte inflammation and M2b macrophage polarization. *Cancer Discov.* **6**, 1182–1195 (2016).
88. Xiao, X. et al. PD-1hi identifies a novel regulatory B cell population in human hepatoma that promotes disease progression. *Cancer Discov.* **6**, 546–559 (2016).
89. Calderaro, J. et al. Intra-tumoral tertiary lymphoid structures are associated with a low risk of early recurrence of hepatocellular carcinoma. *J. Hepatol.* **70**, 58–65 (2018).
**Together with Finkin et al., this paper shows that the location of TLSs is important for their antitumour function. TLSs located in inflamed tumour-adjacent zones have a pro-tumoural role and can serve as a niche for tumour cell progenitors in HCC, favouring late recurrence. Intratumoural TLSs have a favourable prognostic impact on early recurrence in HCC.**
90. Martinet, L. et al. High endothelial venules (HEVs) in human melanoma lesions: Major gateways for tumor-infiltrating lymphocytes. *Oncoimmunology* **1**, 829–839 (2012).
91. Koti, M. et al. Tertiary lymphoid structures associate with tumour stage in urothelial bladder cancer. *Bladder Cancer* **3**, 259–267 (2017).
92. Liu, X. et al. Distinct tertiary lymphoid structure associations and their prognostic relevance in HER2 positive and negative breast cancers. *Oncologist* **22**, 1316–1324 (2017).
93. Figenschau, S. L., Fismen, S., Fenton, K. A., Fenton, C. & Mortensen, E. S. Tertiary lymphoid structures are associated with higher tumor grade in primary operable breast cancer patients. *BMC Cancer* **15**, 101 (2015).
94. Buisseret, L. et al. Tumor-infiltrating lymphocyte composition, organization and PD-1/ PD-L1 expression are linked in breast cancer. *Oncoimmunology* **6**, e1257452 (2017).
95. Cimino-Mathews, A. et al. PD-L1 (B7-H1) expression and the immune tumor microenvironment in primary and metastatic breast carcinomas. *Hum. Pathol.* **47**, 52–63 (2016).
96. Schweiger, T. et al. Tumor-infiltrating lymphocyte subsets and tertiary lymphoid structures in pulmonary metastases from colorectal cancer. *Clin. Exp. Metastasis* **33**, 727–739 (2016).
97. Shields, J. D., Kourtis, I. C., Tomei, A. A., Roberts, J. M. & Swartz, M. A. Induction of lymphoidlike stroma and immune escape by tumors that express the chemokine CCL21. *Science* **328**, 749–752 (2010).
98. Colbeck, E. J. et al. Treg depletion licenses T cell–driven HEV neogenesis and promotes tumor destruction. *Cancer Immunol. Res.* **5**, 1005–1015 (2017).
99. Hindley, J. P. et al. T cell trafficking facilitated by high endothelial venules is required for tumor control after regulatory T cell depletion. *Cancer Res.* **72**, 5473–5482 (2012).
100. Neyt, K., GeurtsvanKessel, C. H., Deswarte, K., Hammad, H. & Lambrecht, B. N. Early IL-1 signaling promotes iBALT induction after influenza virus infection. *Front. Immunol.* **7**, 312 (2016).
101. Kennedy, C. L. et al. The molecular pathogenesis of STAT3-driven gastric tumourigenesis in mice is independent of IL-17. *J. Pathol.* **225**, 255–264 (2011).
102. Barone, F. et al. IL-22 regulates lymphoid chemokine production and assembly of tertiary lymphoid organs. *Proc. Natl Acad. Sci. USA* **112**, 11024–11029 (2015).
103. Giraldo, N. A. et al. Orchestration and prognostic significance of immune checkpoints in the microenvironment of primary and metastatic renal cell cancer. *Clin. Cancer Res.* **21**, 3031–3040 (2015).
104. Giraldo, N. A. et al. Tumor-infiltrating and peripheral blood T cell immunophenotypes predict early relapse in localized clear cell renal cell carcinoma. *Clin. Cancer Res.* **23**, 4416–4428 (2017).
105. Giraldo, N. A., Becht, E., Vano, Y., Sautès-Fridman, C. & Fridman, W. H. The immune response in cancer: from immunology to pathology to immunotherapy. *Virchows Arch.* **467**, 127–135 (2015).
106. Remark, R. et al. Characteristics and clinical impacts of the immune environments in colorectal and renal cell carcinoma lung metastases: influence of tumor origin. *Clin. Cancer Res.* **19**, 4079–4091 (2013).
**This study shows that the tumour origin dictates the prognostic impact of CD8$^{+}$ T cells in lung metastases.**
107. Meshcheryakova, A. et al. B cells and ectopic follicular structures: novel players in anti-tumor programming with prognostic power for patients with metastatic colorectal cancer. *PLOS ONE* **9**, e99008 (2014).
108. Finkelman, F. D. et al. Lymphokine control of in vivo immunoglobulin isotype selection. *Annu. Rev. Immunol.* **8**, 303–333 (1990).
109. Fridman, W. H., Pagès, F., Sautès-Fridman, C. & Galon, J. The immune contexture in human tumours: impact on clinical outcome. *Nat. Rev. Cancer* **12**, 298–306 (2012).
110. Sharma, P. & Allison, J. P. The future of immune checkpoint therapy. *Science* **348**, 56–61 (2015).
111. Galluzzi, L., Buqué, A., Kepp, O., Zitvogel, L. & Kroemer, G. Immunological effects of conventional chemotherapy and targeted anticancer agents. *Cancer Cell* **28**, 690–714 (2015).
112. Strom, T. et al. Tumour radiosensitivity is associated with immune activation in solid tumours. *Eur. J. Cancer* **84**, 304–314 (2017).
113. Sharabi, A. B., Lim, M., DeWeese, T. L. & Drake, C. G. Radiation and checkpoint blockade immunotherapy: radiosensitisation and potential mechanisms of synergy. *Lancet Oncol.* **16**, e498–e509 (2015).
114. Frederick, D. T. et al. BRAF inhibition is associated with enhanced melanoma antigen expression and a more favorable tumor microenvironment in patients with metastatic melanoma. *Clin. Cancer Res.* **19**, 1225–1231 (2013).
115. Denkert, C. et al. Tumour-infiltrating lymphocytes and prognosis in different subtypes of breast cancer: a pooled analysis of 3771 patients treated with neoadjuvant therapy. *Lancet Oncol.* **19**, 40–50 (2018).
116. Song, I. H. et al. Predictive value of tertiary lymphoid structures assessed by high endothelial venule counts in the neoadjuvant setting of triple-negative breast cancer. *Cancer Res. Treat.* **49**, 399–407 (2017).
117. Stowman, A. M. et al. Lymphoid aggregates in desmoplastic melanoma have features of tertiary lymphoid structures. *Melanoma Res.* **28**, 237 (2018).
118. Eroglu, Z. et al. High response rate to PD-1 blockade in desmoplastic melanomas. *Nature* **553**, 347–350 (2018).
119. Boivin, G. et al. Cellular composition and contribution of tertiary lymphoid structures to tumor immune infiltration and modulation by radiation therapy. *Front. Oncol.* **8**, 256 (2018).
120. Remark, R. et al. Immune contexture and histological response after neoadjuvant chemotherapy predict clinical outcome of lung cancer patients. *Oncoimmunology* **5**, e1255394 (2016).
121. Allen, E. et al. Combined antiangiogenic and anti-PD-L1 therapy stimulates tumor immunity through HEV formation. *Sci. Transl Med.* **9**, eaak9679 (2017).
122. Lutz, E. R. et al. Immunotherapy converts nonimmunogenic pancreatic tumors into immunogenic foci of immune regulation. *Cancer Immunol. Res.* **2**, 616–631 (2014).
123. Johansson-Percival, A. et al. De novo induction of intratumoral lymphoid structures and vessel normalization enhances immunotherapy in resistant tumors. *Nat. Immunol.* **18**, 1207–1217 (2017).
**This paper demonstrates that targeting LIGHT to tumour vessels via a VTP induces formation of intratumoural TLSs and prolongs survival in combination with immune checkpoint inhibitors in mice.**
124. Chen, L. et al. Extranodal induction of therapeutic immunity in the tumor microenvironment after intratumoral delivery of Tbet gene-modified dendritic cells. *Cancer Gene Ther.* **20**, 469–477 (2013).
125. Weinstein, A. M. et al. Tbet and IL-36γ cooperate in therapeutic DC-mediated promotion of ectopic lymphoid organogenesis in the tumor microenvironment. *OncoImmunology* **6**, e1322238 (2017).
126. Weinstein, A. M. et al. Association of IL-36γ with tertiary lymphoid structures and inflammatory immune infiltrates in human colorectal cancer. *Cancer Immunol. Immunother.* **68**, 109–120 (2018).
127. Yang, S.-C. et al. Intrapulmonary administration of CCL21 gene-modified dendritic cells reduces tumor burden in spontaneous murine bronchoalveolar cell carcinoma. *Cancer Res.* **66**, 3205–3213 (2006).
128. Lee, J. M. et al. Phase I trial of intratumoral injection of CCL21 gene-modified dendritic cells in lung cancer elicits tumor-specific immune responses and CD8+ T cell infiltration. *Clin. Cancer Res.* **23**, 4556–4568 (2017).
129. Zhu, G. et al. Induction of tertiary lymphoid structures with antitumor function by a lymph node-derived stromal cell line. *Front. Immunol.* **9**, 1609 (2018).
130. Yagawa, Y. et al. Systematic screening of chemokines to identify candidates to model and create ectopic lymph node structures for cancer immunotherapy. *Sci. Rep.* **7**, 15996 (2017).

131. Zhu, G. et al. Tumor-associated tertiary lymphoid structures: gene-expression profiling and their bioengineering. *Front. Immunol.* **8**, 767 (2017).
132. Weiden, J., Tel, J. & Figdor, C. G. Synthetic immune niches for cancer immunotherapy. *Nat. Rev. Immunol.* **18**, 212–219 (2017).
133. Jones, G. W., Hill, D. G. & Jones, S. A. Understanding immune cells in tertiary lymphoid organ development: it is all starting to come together. *Front. Immunol.* **7**, 401 (2016).
134. Meier, D. et al. Ectopic lymphoid-organ development occurs through interleukin 7-mediated enhanced survival of lymphoid-tissue-inducer cells. *Immunity* **26**, 643–654 (2007).
135. Deteix, C. et al. Intragraft Th17 infiltrate promotes lymphoid neogenesis and hastens clinical chronic rejection. *J. Immunol.* **184**, 5344–5351 (2010).
136. Peters, A. et al. Th17 cells induce ectopic lymphoid follicles in central nervous system tissue inflammation. *Immunity* **35**, 986–996 (2011).
137. Lochner, M. et al. Microbiota-induced tertiary lymphoid tissues aggravate inflammatory disease in the absence of RORgamma t and LTi cells. *J. Exp. Med.* **208**, 125–134 (2011).
138. Guedj, K. et al. M1 macrophages act as LTβR-independent lymphoid tissue inducer cells during atherosclerosis-related lymphoid neogenesis. *Cardiovasc. Res.* **101**, 434–443 (2014).
139. Colbeck, E. J., Ager, A., Gallimore, A. & Jones, G. W. Tertiary lymphoid structures in cancer: drivers of antitumor immunity, immunosuppression, or bystander sentinels in disease? *Front. Immunol.* **8**, 1830 (2017).
140. Furtado, G. C. et al. Lymphotoxin beta receptor signaling is required for inflammatory lymphangiogenesis in the thyroid. *Proc. Natl Acad. Sci. USA* **104**, 5026–5031 (2007).
141. Luther, S. A. et al. Differing activities of homeostatic chemokines CCL19, CCL21, and CXCL12 in lymphocyte and dendritic cell recruitment and lymphoid neogenesis. *J. Immunol.* **169**, 424–433 (2002).
142. Fleige, H. et al. IL-17-induced CXCL12 recruits B cells and induces follicle formation in BALT in the absence of differentiated FDCs. *J. Exp. Med.* **211**, 643–651 (2014).
143. Denton, A. E. et al. Type I interferon induces CXCL13 to support ectopic germinal center formation. *J. Exp. Med.* **216**, 621–637 (2019).

### Acknowledgements
The authors thank members of the Cancer and Immune Escape Team UMR_S1136 Cordeliers Research Centre for fruitful discussions and performing experiments, and L. Lacroix and A. Bougouin for help in preparing figure 1. This work was supported by Institut National Français de Recherche Médicale (INSERM), the University Paris Descartes, Sorbonne University, CARPEM T8, the Labex Immuno-Oncology Excellence Program, Institut du Cancer (INCa), HTE Plan Cancer (C1608DS to C.S.F.), PRTK G26 NIVOREN, Foncer contre le cancer, Bionik contracts and the Cartes d'Identité des Tumeurs (CIT) Program from the Ligue Nationale Contre le Cancer. F.P. was supported by a CARPEM doctorate fellowship, and J.C. was supported by an Association pour la Recherche sur le Cancer (ARC) postdoctoral fellowship.

### Author contributions
All authors reviewed and edited the manuscript before submission.

### Competing interests
C.S.-F. and W.H.F. are on the list of co-inventors on a patent application for the prognostic impact of tertiary lymphoid structures in patients with lung cancer (PCT-EP2016/070158). F.P. and J.C. declare no competing interests.

### Publisher's note
Springer Nature remains neutral with regard to jurisdictional claims in published maps and institutional affiliations.

### Reviewer information
*Nature Reviews Cancer* thanks J. Mule and the other anonymous reviewer(s) for their contribution to the peer review of this work.

### RELATED LINKS
**MCP-counter:** https://cit.ligue-cancer.net/mcp-counter/
**National Cancer Institute (NCI) Genomic Data Commons (GDC):** https://gdc.cancer.gov/
