# Supplementary material for: Tertiary lymphoid structures-driven immune infiltration patterns and their association with survival in neuroblastoma
Source: PeerJ. 2025 Jul 22;13:e19767. doi: 10.7717/peerj.19767 (PMC12292307; doi:10.7717/peerj.19767)
Supplement: Supplemental Information 7 [file peerj-13-19767-s007.docx]

The English translation corresponding to the Chinese text in the Supplementary-3

| Chinese | English |
| --- | --- |
| 基于ssGSEA的方法计算血管生成得分 | Calculate the angiogenesis score based on the ssGSEA method |
| 通过下载HALLMARK_EPITHELIAL_MESENCHYMAL_TRANSITION通路中的200个基因，通过ssGSEA的方法计算EMT的评分 | By downloading 200 genes from the HALLMARK_EPITHELIAL_MESENCHYMAL_TRANSITION pathway, the EMT score was calculated using the ssGSEA method. |
| 通过下载HALLMARK_HYPOXIA的基因，通过ssGSEA的方法计算缺氧的评分 | By downloading the genes from HALLMARK_HYPOXIA, the hypoxia score was calculated using the ssGSEA method. |
| 基于ssGSEA的方法计算KEGG官网信号通路相关基因集评分 | Calculate the score of gene sets related to KEGG signaling pathways using the ssGSEA method |
| 基于Progenitor Cell Biology Consortium (PCBC)(https://www.synapse.org) 提供的人类干细胞数据集使用OCLR（One Class Linear Regression）的方法，对肿瘤样本的干性进行量化 | The stemness of tumor samples was quantified using the OCLR (One-Class Linear Regression) method based on the human stem cell dataset provided by the Progenitor Cell Biology Consortium (PCBC) ([https://www.synapse.org](https://www.synapse.org/)). |
| 多因素模型变量 | Multifactor model variables |
| 系数 | Coefficient |
| 多因素Cox模型情况 | Multivariate Cox model situation |
