# Supplementary material for: Tertiary lymphoid structures-driven immune infiltration patterns and their association with survival in neuroblastoma
Source: PeerJ. 2025 Jul 22;13:e19767. doi: 10.7717/peerj.19767 (PMC12292307; doi:10.7717/peerj.19767)
Supplement: Supplemental Information 10 [file peerj-13-19767-s010.docx]

****Genotyping Results of STR Loci and Amelogenin Locus in Cells****

| ****Submitted Cell Name: SHSY5Y**** | | | | ****Cell Bank Cell Name: SHSY5Y**** | | | |
| --- | --- | --- | --- | --- | --- | --- | --- |
| **Locus** | **Allele 1** | **Allele 2** | **Allele 3** | **Locus** | **Allele 1** | **Allele 2** | **Allele 3** |
| D5S818 | 12 | 12 | - | D5S818 | 12 | 12 | - |
| D13S317 | 11 | 11 | - | D13S317 | 11 | 11 | - |
| D7S820 | 7 | 10 | - | D7S820 | 7 | 10 | - |
| D16S539 | 8 | 13 | - | D16S539 | 8 | 13 | - |
| VWA | 14 | 18 | - | VWA | 14 | 18 | - |
| TH01 | 7 | 10 | - | TH01 | 7 | 10 | - |
| AMEL | X | X | - | AMEL | X | X | - |
| TPOX | 8 | 11 | - | TPOX | 8 | 11 | - |
| CSF1PO | 11 | 11 | - | CSF1PO | 11 | 11 | - |
| D12S391 | 18 | 22 | - | D12S391 | - | - | - |
| FGA | 23 | 24 | - | FGA | - | - | - |
| D2S1338 | 17 | 19 | - | D2S1338 | - | - | - |
| D21S11 | 31 | 31 | - | D21S11 | - | - | - |
| D18S51 | 13 | 16 | - | D18S51 | - | - | - |
| D19S433 | 13 | 14 | - | D19S433 | - | - | - |
| D1S1656 | 12 | 12 | - | D1S1656 | - | - | - |
| D3S1358 | 15 | 16 | - | D3S1358 | - | - | - |
| D6S1043 | 12 | 18 | - | D6S1043 | - | - | - |
| PENTA E | 7 | 11 | - | PENTA E | - | - | - |
| PENTA D | 10 | 12 | - | PENTA D | - | - | - |
| D11S2367 | 15 | 15 | - | D11S2367 | - | - | - |
