# Supplementary material for: Tertiary lymphoid structures-driven immune infiltration patterns and their association with survival in neuroblastoma
Source: PeerJ. 2025 Jul 22;13:e19767. doi: 10.7717/peerj.19767 (PMC12292307; doi:10.7717/peerj.19767)
Supplement: Supplemental Information 6 [file fig9b.pdf › Raw Data/RNA-seq/19.TIP/results/fig9b.pdf]

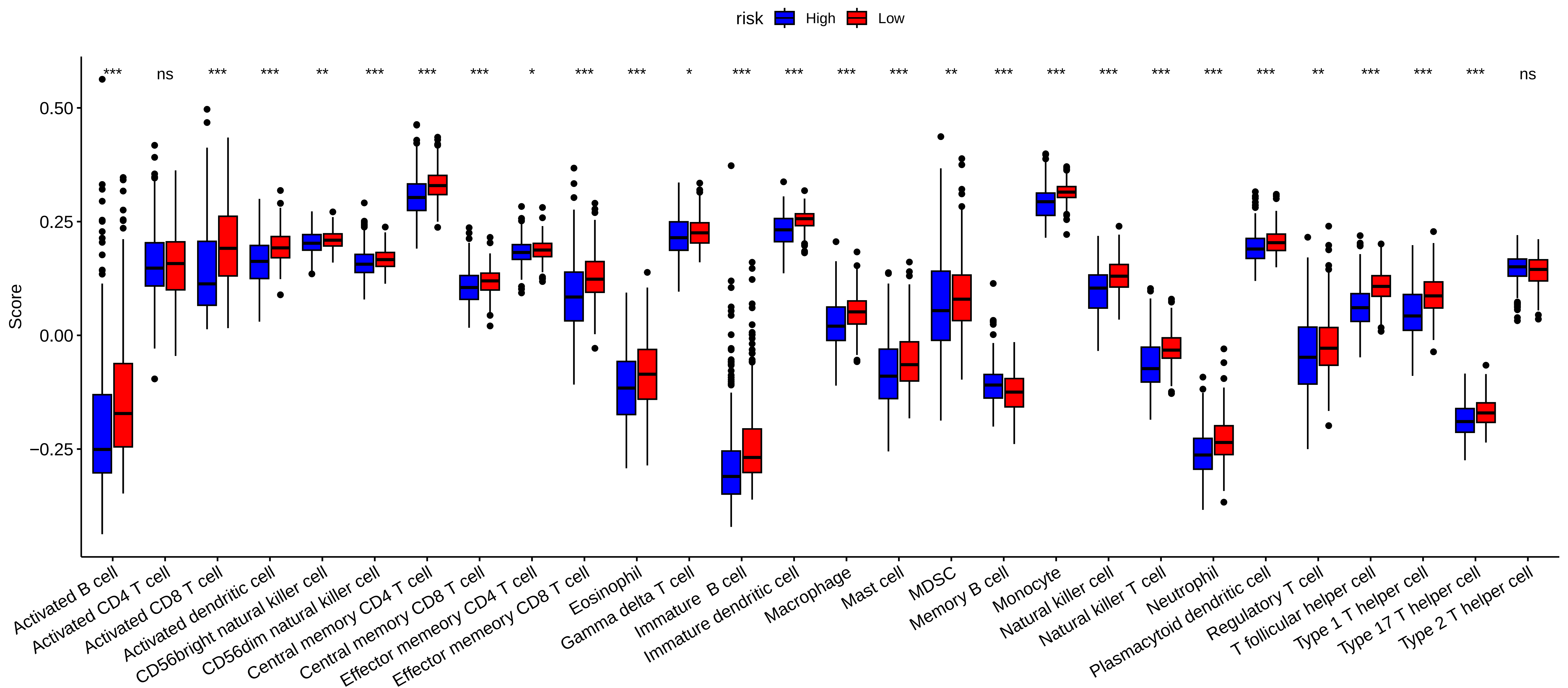

risk High Low
Score
0.50
0.25
0.00
−0.25
*** ns *** *** ** *** *** *** * *** *** * *** *** *** *** ** *** *** *** *** *** *** ** *** *** *** ns
Activated B cell
Activated CD4 T cell
Activated CD8 T cell
Activated dendritic cell
CD56bright natural killer cell
CD56dim natural killer cell
Central memory CD4 T cell
Central memory CD8 T cell
Effector memeory CD4 T cell
Effector memeory CD8 T cell
Eosinophil
Gamma delta T cell
Immature B cell
Immature dendritic cell
Macrophage
Mast cell
MDSC
Memory B cell
Monocyte
Natural killer cell
Natural killer T cell
Neutrophil
Plasmacytoid dendritic cell
Regulatory T cell
T follicular helper cell
Type 1 T helper cell
Type 17 T helper cell
Type 2 T helper cell

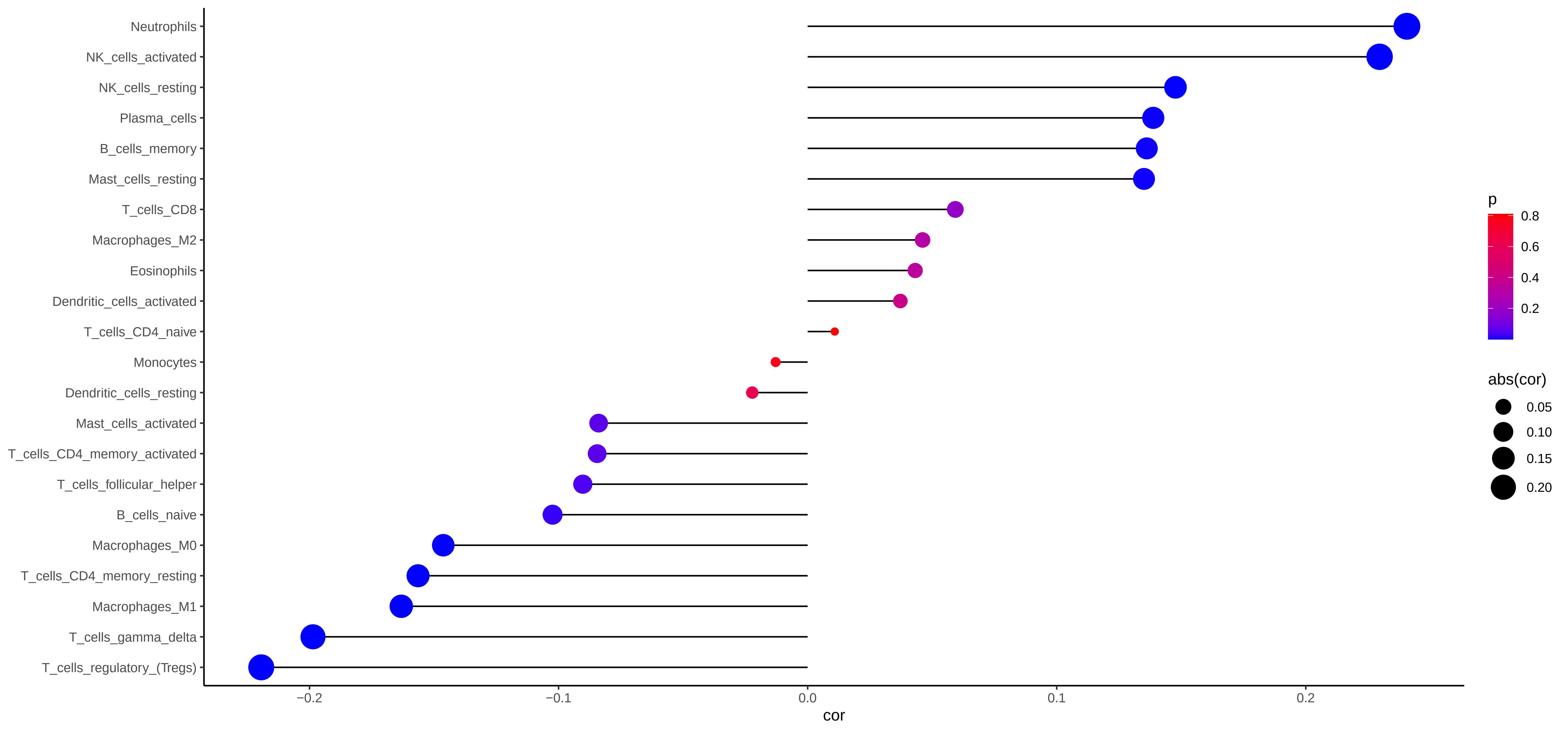

Neutrophils
NK_cells_activated
NK_cells_resting
Plasma_cells
B_cells_memory
Mast_cells_resting
T_cells_CD8
Macrophages_M2
Eosinophils
Dendritic_cells_activated
T_cells_CD4_naive
Monocytes
Dendritic_cells_resting
Mast_cells_activated
T_cells_CD4_memory_activated
T_cells_follicular_helper
B_cells_naive
Macrophages_M0
T_cells_CD4_memory_resting
Macrophages_M1
T_cells_gamma_delta
T_cells_regulatory_(Tregs)
−0.2
−0.1
0.0
0.1
0.2
cor
p
0.8
0.6
0.4
0.2
abs(cor)
0.05
0.10
0.15
0.20

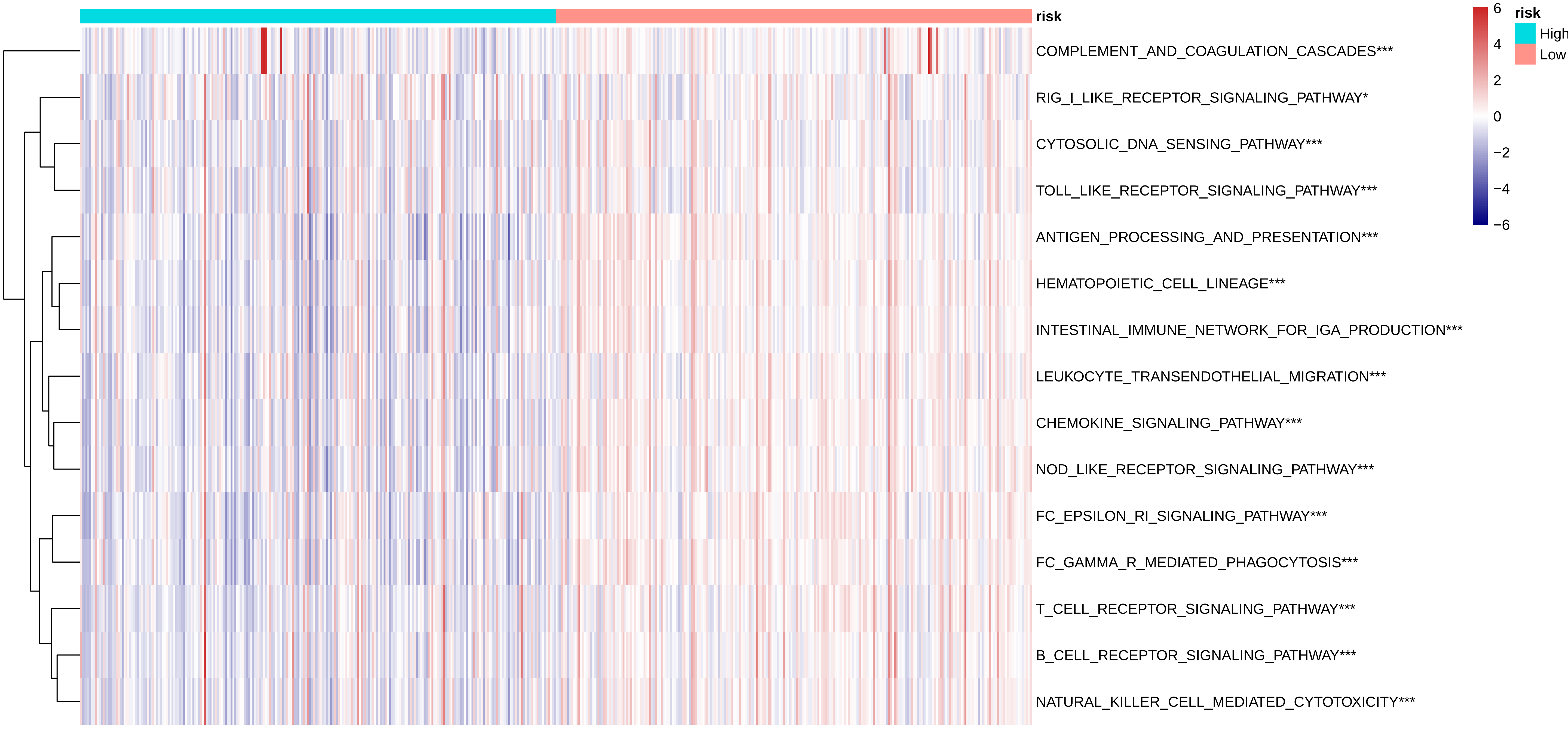

risk
COMPLEMENT_AND_COAGULATION_CASCADES***
RIG_I_LIKE_RECEPTOR_SIGNALING_PATHWAY*
CYTOSOLIC_DNA_SENSING_PATHWAY***
TOLL_LIKE_RECEPTOR_SIGNALING_PATHWAY***
ANTIGEN_PROCESSING_AND_PRESENTATION***
HEMATOPOIETIC_CELL_LINEAGE***
INTESTINAL_IMMUNE_NETWORK_FOR_IGA_PRODUCTION***
LEUKOCYTE_TRANSENDOTHELIAL_MIGRATION***
CHEMOKINE_SIGNALING_PATHWAY***
NOD_LIKE_RECEPTOR_SIGNALING_PATHWAY***
FC_EPSILON_RI_SIGNALING_PATHWAY***
FC_GAMMA_R_MEDIATED_PHAGOCYTOSIS***
T_CELL_RECEPTOR_SIGNALING_PATHWAY***
B_CELL_RECEPTOR_SIGNALING_PATHWAY***
NATURAL_KILLER_CELL_MEDIATED_CYTOTOXICITY***
6
4
2
0
−2
−4
−6
risk
High
Low
